# Design and Synthesis of New Derivatives of Alkaloid Lupinine: Structural Characterization, Acetylcholinesterase Inhibition Evaluation, and Molecular Modeling Study

**DOI:** 10.3390/molecules31213961

**Published:** 2026-11-03

**Authors:** Zhangeldy S. Nurmaganbetov, Igor D. Ivanov, Oralgazy A. Nurkenov, Victor A. Savelyev, Andrey I. Khlebnikov, Dastan M. Turdybekov, Aizhan M. Kozhanova, Serik D. Fazylov, Zainulla M. Muldakhmetov, Elvira E. Shults

**Affiliations:** 1 Laboratory of Synthesis of Biologically Active Substances, Institute of Organic Synthesis and Coal Chemistry, Karaganda 100008, Kazakhstan; nurkenov_oral@mail.ru (O.A.N.); nurs-djoni@mail.ru (A.M.K.); iosu8990@mail.ru (S.D.F.); iosu.rk@mail.ru (Z.M.M.); 2 Institute of Molecular Biology and Biophysics, Timakova Str 14, Novosibirsk 630090, Russia; diadoryh@mail.ru; 3 N.N. Vorozhtsov Novosibirsk Institute of Organic Chemistry, Siberian Branch of the Russian Academy of Sciences, Novosibirsk 630090, Russia; vicsav@nioch.nsc.ru; 4 Kizhner Research Center, Tomsk Polytechnic University, Tomsk 634050, Russia; aikhl@chem.org.ru; 5 Department of Physics, Abylkas Saginov Karaganda Technical University, Karaganda 100027, Kazakhstan; turdas@mail.ru

**Keywords:** alkaloids, lupinine, methylxanthines, colchicine, A^3^-coupling reaction, CuAAC reaction, acetylcholinesterase inhibitor, molecular docking study

## Abstract

Plant alkaloids have been of great interest in drug development research. Alkaloid lupinine is of great interest because of its availability and biological activity. While several lupinine derivatives have been identified with specific biological activities, the ongoing challenge lies in the design and synthesis of novel derivatives with high specificity for additional pharmacological targets. This paper describes the synthesis of lupinine derivatives as acetylcholinesterase (AChE) inhibitors. Two approaches were proposed and implemented for the synthesis of new compounds (yields 71–90%): the CuAAC reaction of lupinyl azide with a series of prop-2-ynyl- and ethynyl-substituted alkaloid derivatives, and a copper-catalyzed one-pot three-component reaction (A^3^-coupling) involving 1-((prop-2-ynyloxy)methyl)octahydro-1*H*-quinolizine, formaldehyde, and secondary amines. The biological activity of the new compounds was evaluated using the Ellman method; the results showed that most of the synthesized substances demonstrated good or moderate inhibitory activity against acetylcholinesterase in vitro. Hybrid compounds containing lupinine and xanthine fragments (theophylline, caffeine) **11**, **12**, or colchicine **15**, as well as the bis-lupinyl derivative **16**, exhibited the highest inhibitory activity against AChE with IC50 values of 0.47, 0.035, 1.3, and 1.4 μM, respectively. The binding conformation and simultaneous interaction modes were further clarified by molecular docking studies.

## 1. Introduction

Alkaloids represent a promising source for the discovery of new therapeutic agents, due to the diversity of their chemical structures and pharmacological activities. The neuroprotective properties of alkaloids in Alzheimer’s disease (AD) are attributed to their ability to regulate processes such as beta-amyloid aggregation, oxidative stress, synaptic dysfunction, tau protein hyperphosphorylation, neuroinflammation, and, also, cholinesterase inhibition. The antiacetylcholinesterase activity is a significant and medically relevant biological effect. This activity is particularly important because it has the potential to inhibit the degradation of acetylcholine, a neurotransmitter released in the synaptic clefts.

Previous studies have shown that quinolizidine (octahydro-*2H*-quinolizine) alkaloids, characterized by a structural feature of two fused six-membered rings that share nitrogen, possess the ability to inhibit acetylcholinesterase [1,2]. Within this class, the readily available bicyclic alkaloid (−)-*L*-lupinine ([(1*R*,9a*R*)-octahydro-2H-quinolizin- 1-yl]methanol) **1** is of particular interest, due to its low toxicity and genotoxicity [3,4,5]. Significant attention has been devoted to the isolation of this alkaloid from plants of the *Lupinus* and *Anabasis* genera, as well as to the study of its biological activity [2,5,6,7]. Lupinine **1** provides a skeletal structure that can be easily modified for further optimization. Alkaloid **1**, along with epilupinine and lupinylamine, has been described as a ligand for 5-HT3 and 5-HT4 serotonin receptors [8]. The ability of 2-(4R-phenoxy/phenylthio) alkanoic esters of *L*-lupinine to displace quinuclidinyl benzilate ([3H]QNB) from rat brain preparation was investigated, and almost all the studied esters showed good affinity to muscarinic receptors [9]. Isomeric derivatives of lupinine and epilupinine exhibited differential cholinesterase inhibition, and the variation in activity was attributed to substituent at C-10 position of the molecules, and also to the source of enzymes [10,11,12,13,14,15]. The aryltriazole derivatives of *L*-lupinine exhibited potent AChE inhibitory activity (IC_50_ = 7.18 μM), and kinetic analysis demonstrated that these compounds belong to the mixed-type AChE inhibitors [15].

**Figure 1:**
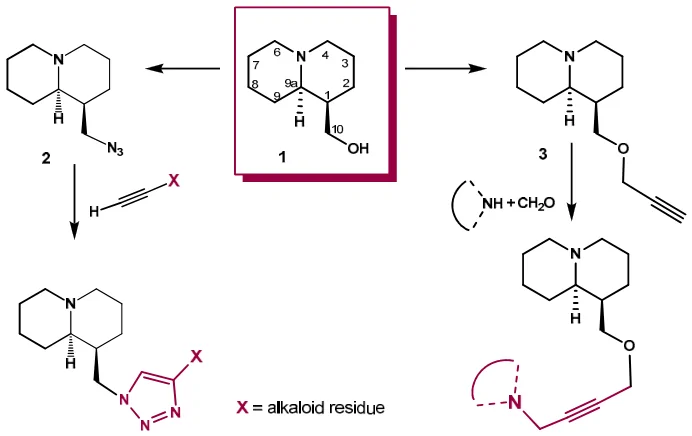
Design of novel AChE inhibitors based on quinolizidine scaffold.

Given the critical role of acetylcholinesterase inhibition in the development of agents for AD treatment, recent research has focused on the design of multifunctional cholinesterase inhibitors. These agents combine enzymatic inhibition with antioxidant, anti-inflammatory, and neuroprotective activities, thereby addressing the multifactorial nature of neurodegenerative processes [16]. We hypothesized that molecules with improved or synergistic biological properties could exert such an effect. This study aimed to design, synthesize, and characterize a novel series of lupinine-based hybrids incorporating well-known Plant alkaloid cores–anabisine, (−)-cytisine, xantines (theophyline, caffeine, and theobromine) or (−)-colchicine (Figure 1). Alkaloid anabazine (3-[(2*S*)-piperidin-2-yl]pyridine) was used as a chiral scaffold in various synthesis [17,18]. Anabasine and its derivatives have been characterized as cholinesterase inhibitors, as well as agonists of nicotinic acetylcholine receptors, which are currently considered as important targets in the drug discovery efforts aimed at treating neurological disorders [18,19]. Since the early pharmacological studies, cytisine derivatives were characterized as reversible inhibitors of cholinesterases. Of interest, nitrogen substitution modifies the activity of cytisine on neuronal nicotinic receptor subtypes, suggesting that the most relevant targets of these compounds are cholinergic nicotinic acetylcholine receptors (AChRs) [20,21]. The natural methylxanthines caffeine, theobromine, and theophylline are widely used in traditional medicines, and also in the field of biological and medicinal chemistry [22]. In the past years, the impact of methylxanthines in neurodegenerative diseases has been extensively studied, and several new aspects have been elucidated [23,24,25], which show that synthesis and pharmacological analysis of such xanthine derivatives could be a promising approach in therapies of neurodegenerative diseases. (−)-Colchicine, a toxic alkaloid derived from *Colchicum autumnale*, is being used in rheumatology, dermatology, cardiology, and neurology. Beyond being used for treatment of gout and FMF, colchicine is also routinely used in other rheumatic diseases The use of (−)-colchicine for the treatment of arrhythmia and ischemic heart disease is currently being investigated through clinical trials [26,27,28]. Synthetic transformation of (−)-colchicine have been employed to modify physicochemical and bioactivity profiles [29]. Recent studies on colchicines-aryl/alkyl amine hybrids have shown their dual action in inhibiting cholinesterase activity and suppressing NLRP3 inflammasome activation, making them promising candidates for addressing both cholinergic dysfunction and neuroinflammation in AD [30].

We employed a 1,2,3-triazole pharmacofore as a linker to connect two alkaloid moieties within the structure. The 1,2,3-triazole structural fragment has emerged as a favored choice because of its properties, and could play a major role in the common mechanisms associated with various disorders like cancer, inflammation and neurodegeneration [31,32,33]. We also considered propargylamine as a pharmacophore, given its successful use in developing CNS-targeted multi-target directed ligands for neurodegenerative disorders [33,34,35,36,37]. As represented by such modified preparation as ladostigil, donepezil-based hybrid ASS234, memantine-propargylamine, tacrine-selegiline, and chromene-propargylamine hybrids [38,39,40,41,42], the propargylamine fragment has been identified as being essential for the beneficial effects of these compounds. We hypothesized that the modification of the octahydro-1H-quinolizine core via conjugation with nitrogen-containing pharmacophoric fragments through a rigid acetylene linker could be a promising strategy for the development of potent cholinesterase inhibitors.

In this context, the present work has focused on the study of the copper-catalyzed 1,3-dipolar cycloaddition reaction of lupinyl azide **2** with a diversity of alkynyl-substituted plant alkaloids and the three-component one-pot copper-catalyzed Mannich reaction of 10-*O*-(prop-2-ynyl)lupinine **3**, formaldehyde, and secondary amines (dialkylamines, piperidine, azepane). We have synthesized six triazole-linked hybrid compounds containing methyloctahydro-1*H*-quinolizine and plant alkaloid fragments, one dimeric compound with two lupinine residues, and four N-substituted 4-(aminobut-2-ynyloxy)methyloctahydro-1*H*-quinolizine derivatives, and evaluated them for AChE inhibitory activity. This screening resulted in the identification of some novel AChE inhibitors, with the most potent being compound **12**. To better understand the key structural requirements for effective receptor–ligand interactions, in parallel with the biological evaluation, docking studies have also been performed.

## 2. Results

### 2.1. Chemical Synthesis

**Scheme 1:**
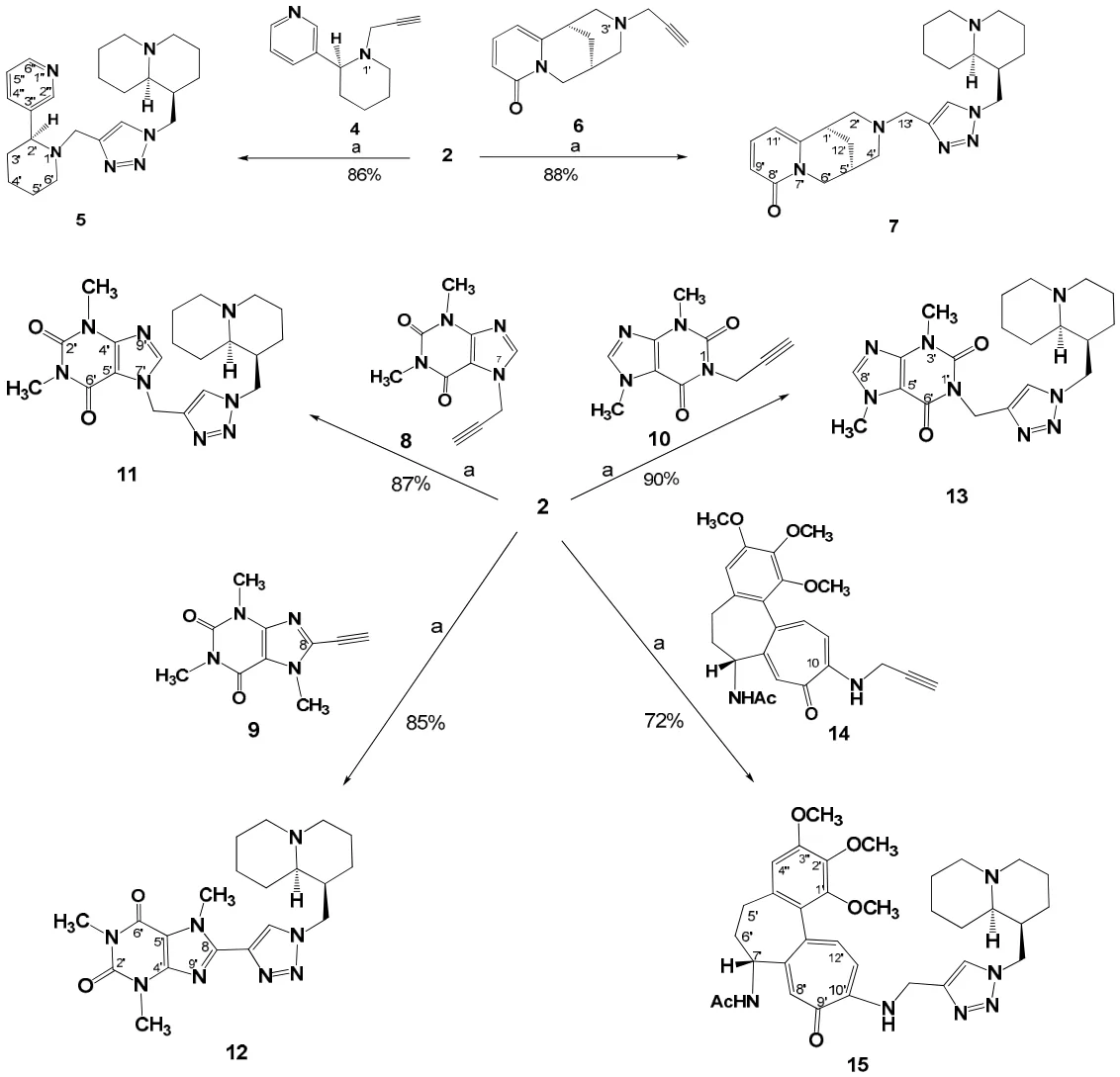
Synthesis of triazole-linked hybrid compounds **6**, **7**, **11–13**, **15** by CuAAC reaction of lupinylazide **2** with 8-ethynylcaffeine **9** or N-(prop-2-ynyl) derivatives of plant alkaloids (**4**, **5**, **8**, **10**, **14**). Reagents and conditions: (a) CuSO_4_·5H_2_O, sodium ascorbate, DMF, 75 °C, 5–8 h.

The reaction of lupinyl azide **2** [43] with N-(prop-2-ynyl)anabazine **4** (1 equiv.) in DMF in the presence of sodium ascorbate (15 mol%) and copper(II) sulfate (5 mol%) (the conditions used in our previous studies [15,43,44]) gave the 1-(4-(((*S*)-2-(pyridine-3-yl)piperidin-1-yl)methyl)-1*H*-1,2,3-triazol-1-yl)methyl)octahydro-1*H*-quinolizine **5** (yield 86%) (Scheme 1). Reaction of azide **2** with 3-(prop-2-ynyl)cytisine **6** produced an 88% yield of the octahydroquinolizine **7,** containing a (cytisinylmethyl)triazole substituent. We found that the reaction of azide **2** with alkynylxanthines (7-(prop-2-yn-1-yl)theophylline **8**, 8-ethynylcaffeine **9**, and 1-(prop-2-yn-1-yl)-3,7-dimet-hylxanthine **10** (from theobromine) proceeded well, and led to hybrid compound **11**–**13** isolated in 85–90% yield. The copper-mediated reaction of azide **2** with 10-propargylamino-substituted colchicine **14** afforded the targeted octahydro-1*H*-quinolizine-colchicine hybrid compound **15** in 72% isolated yield.

**Scheme 2:**
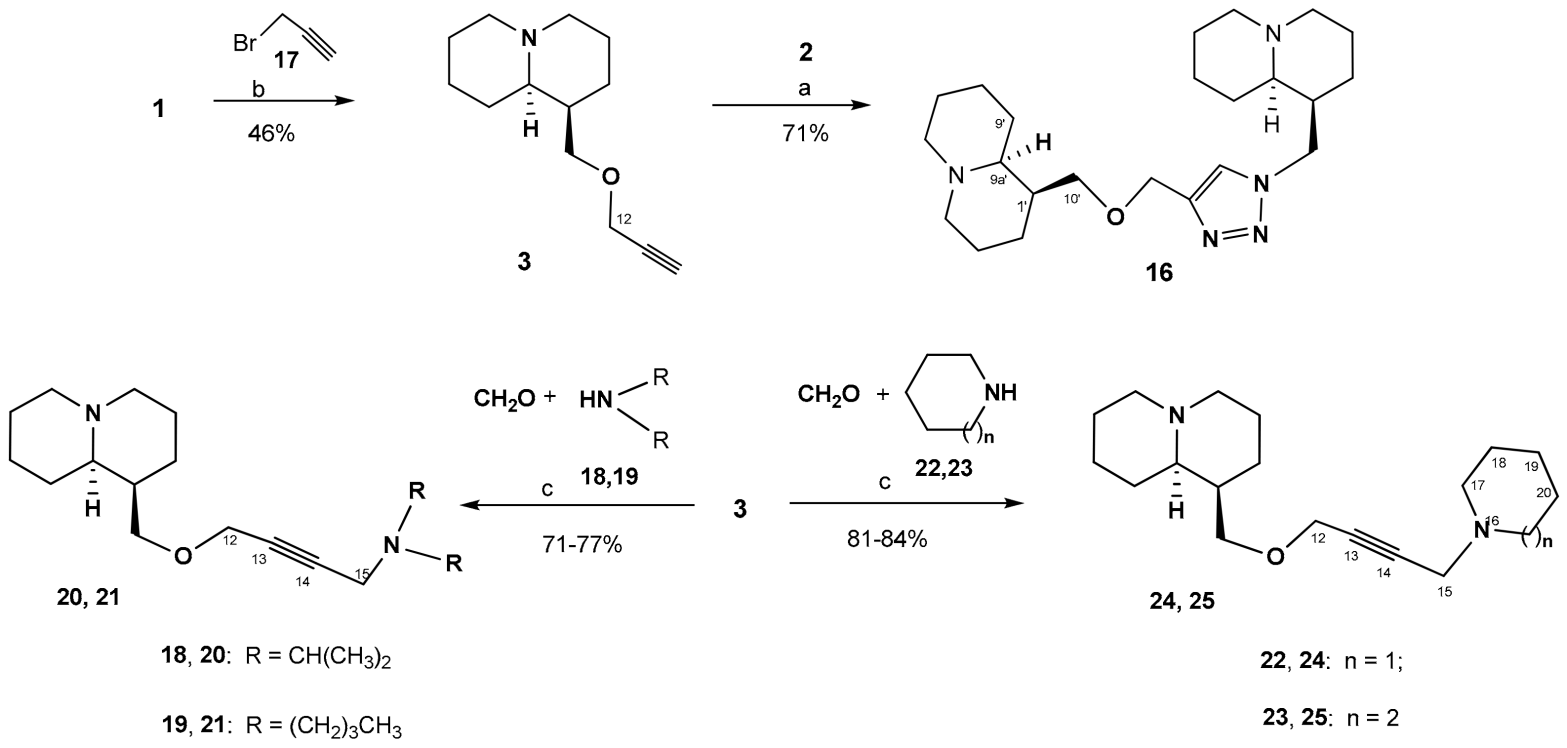
Synthesis of methyltriazolyloxymethyl-linked *bis*-octahydro-1*H*-quinolizine **16** and N-substituted (4-(amino)but-2-ynyloxy)methyl)octahydroqiunolizines **20**, **21**, **24**, **25**. Reagents and conditions: (a) CuSO_4_·5H_2_O, sodium ascorbate, DMF, 75 °C, 6 h; (b) NaH (60% in oil), DMSO, 10 → 20 °C, 10 h; (c) Cu(OAc)_2_·H_2_O, 1,4-dioxane, 65–70 °C, 5–8 h.

Given the valuable pharmacological properties of bisquinolizidine alkaloids [1,45,46], we have developed a method for the synthesis of bis-octahydro-1*H*-quinolizine derivatives linked by a methyltriazolylmethoxymethyl bridge (compound **16**) (Scheme 2). To this end, azide **2** was subjected to an azide–alkyne cycloaddition reaction with a novel alkyne, 10-*O*-(prop-2-ynyl)lupinine **3**. Compound **3** was synthesized by the reaction of (−)-lupinine **1** with propargyl bromide **17** in the presence of sodium hydride (isolated yield 46%). The 1,3-dipolar reaction of lupinyl azide **2** with alkyne **3** was realized in DMF medium in the presence of sodium ascorbate (15 mol%) and copper(II) sulfate (5 mol%) by heating to 75 °C (TLC control). Compound **16** was isolated from the reaction mixture ( yield 71%).

(1*R*,9a*R*)-1-((Prop-2-ynyloxy)methyl)octahydro-1*H*-quinolizine **3** was also utilized as an alkyne component in the A^3^-coupling reaction. The interaction of compound **3** with an aq. formaldehyde solution (4 equiv.) and diisopropylamine **18** or dibutylamine **19** (1.8 equiv.) in the presence of copper(II) acetate (5% mol) in 1,4-dioxane proceeded smoothly, and, by heating at 70 °C for 5 h, the alkyne **3** was almost completely consumed (TLC control). Following work-up and column chromatography, the target 9-((4-(dialkylamino)but-2-ynyloxy)methyl)octahydro-1*H*-quinolizines **20**, **21** were isolated in good yields (71–77%) (Scheme 2). The reaction of alkyne **3** with aq. formaldehyde and cyclic secondary amines (piperidine **22** and azepane **23**) required a longer duration, and resulted in the formation of 10-(4-(piperidin-1-yl)- but-2-ynyloxy)octahydro-1*H*-quinolizine **24** or 10-(4-(azepan-1-yl)- but-2-ynyloxy)octahydro-1*H*-quinolizine **25** (yields 81–84%) (Scheme 2).

**Figure 2:**
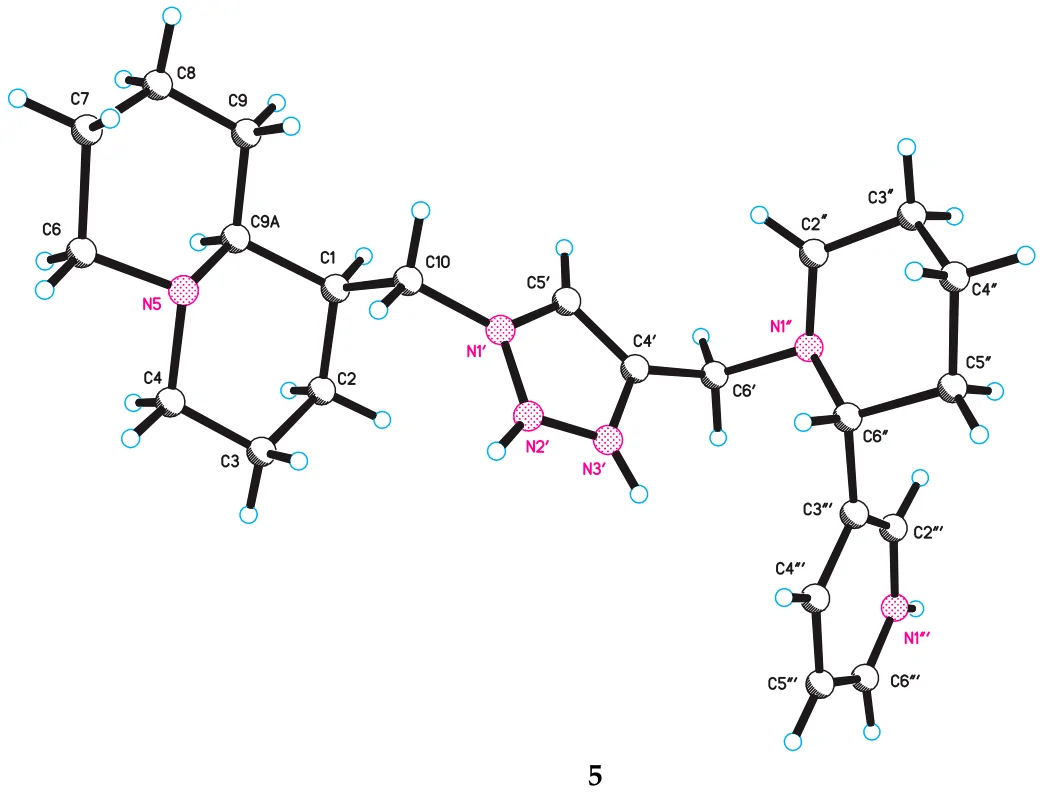
X-ray crystal structure of (1*S*,9a*R*)-1-((4-(((*S*)-2-(pyridin-3-yl)piperidin-1-yl)methyl)-1*H*-1,2,3-triazol-1-yl)methyl)octahydro-1*H*-quinolizine (**5**) (CCDC 2562170).

The structures of the novel compounds were confirmed by ^1^H and ^13^C NMR, IR and HR-MS data (Supplementary Materials, Figures S1–S39). The ^1^H and ^13^C NMR spectra of novel lupinine derivatives (recorded in CDCl_3_) were in good agreement with their structure, and contained one set of characteristic signals of the lupinine core, the corresponding substituents, and the triazole or butynyloxy linker group. In particular, the ^1^H NMR spectra of hybrid compounds **5**, **7**, **11**–**13**, and **15** contain the resonance signals of the 10-CH_2_ groups, which are represented as dd of two protons at the range of δ 4.42–4.70 and δ 4.33–4.60 ppm and a singlet signal of the 1,2,3-triazole H-5 proton (δ 6.94–8.11 ppm). The IR spectrum of final compounds **21**, **24**, and **25** showed C≡C stretching vibration around 2110–2180 cm^−1^ (as broad absorption bands). In the ^13^C NMR spectra, the signals of the triple-bond carbon atoms appear at δC 79.29–81.05 (C-13) and δC 81.02–85.14 (C-14) ppm. These chemical-shift values are characteristic of di-substituted alkynes. The ESI mass spectrometric data of all the synthesized compounds showed peaks at relevant [M]^+^ m/z, which complemented the IR and NMR spectra regarding the confirmation of expected structures of all the compounds. Additionally, the structure of compound **5** was confirmed by X-ray single-crystal diffraction study (Figure 2). The bond lengths, valence angles, and torsion angles in the quinolizidine framework are standard, and consistent with those found in the structure of lupinine hydrochloride [47] and the molecules previously studied by us: (1*S*,9a*R*)-1-({4-[4-(benzyloxy)-3-methoxyphenyl]-1*H*-1,2,3-triazol-1-yl}methyl)octahydro-2*H*-quinolizine [48] and (1*S*,9a*R*)-1-((4-(4-methoxyphenyl)-1*H*-1,2,3-triazol-1-yl)methyl)-octahydro-1*H*-quinolizine [43]. The conformations of the six-membered rings N5,C6…N9a (5) are similar to those in the crystal structure of the molecule. The orientation of the 1,2,3-triazole ring relative to the octahydroquinolizine framework is pseudo-equatorial, with the C9a-C1-N1′-N2′ torsion angle being −108.9°. The 1,2,3-triazole ring is planar within 0.07 Å. The piperidine ring in molecule **5** is oriented axially, relative to the triazole ring (torsion angle C4′C6′N1″C6″ = 69.0°). The piperidine ring N1″C2″C3″C4″C5″C6″ in molecule **5** adopts a nearly ideal chair conformation (asymmetry parameters ΔC2 2″,3″ = 0.51, ΔCs 2″ = 0.76).

### 2.2. Enzyme Inhibition

The synthesized 10-*O*-(prop-2-ynyl)lupinine **3**, triazole-linked hybrid compounds **5**, **7**, **11**–**13**, **15**, **16**, substituted at the 4-nitrogen atom 4-(R-amino)but-2-ynyloxy)methyl)octahydro-1*H*-qiunolizines **21**, **24**, **25**, and the parent compounds (−)-lupinine 1, (−)-colchicine **14** were evaluated for their ability to inhibit AChE from electric eel (EeAChE), using Ellman’s method [49]. The enzymatic activity was quantified indirectly by spectrophotometric determination of the 5-thio-2-nitrobenzoate concentration formed in the reaction between the reagent 5,5′-dithio-bis(2-nitrobenzoic acid) and thiocholine, a product of acetylthiocholine hydrolysis catalyzed by cholinesterases. The efficacy of inhibitors is expressed as IC_50_, i.e., the concentration required for 50% inhibition of the enzyme activity obtained from three independent experiments. Results are listed in Table 1. Galantamine, a clinically used drug acting as a balanced inhibitor of AChE, was used as a standard for comparing IC_50_ values. It is evident that the substituent at the C-10 position of lupinine has a significant effect on AChE inhibition. It should be noted that four new triazole-linked bis-alkaloids (**11**, **12**, **15**, and **16**) are more potent AChE inhibitors than galantamine, and the activity of 10-(4-(piperidin-1-yl)but-2-ynyloxy-**24** and 10-(4-(azepan-1-yl)but-2-ynyloxy)octahydro-1*H*-quinolizine **25** was comparable to a galantamine positive control. The obtained data indicated that the caffeine-octahydro-1*H*-quinolizine hybride **12** represented the most potent suppression activity (Table 1).

## 3. Discussion

In recent years, numerous attempts have been made to modify multifunctional plant alkaloids under mild reaction conditions using simple and selective methodologies, including one-pot synthesis strategies. In the present work, we investigated the transformations of previously obtained 10-azidolupinine **2** [43], as well as the novel lupinine derivatives (1*R*,9a*R*)-1-((prop-2-ynyloxy)methyl)octahydro-1*H*-quinolizine **3**. We employed two synthetic approaches: copper-catalyzed azide–alkyne 1,3-dipolar cycloaddition and the copper catalyzed three-component A^3^-coupling reaction of an alkyne, aldehyde, and amine.

As shown in Scheme 1, our strategy using the readily available lupinyl azide **2** is based on the possibility of regioselective synthesis of octahydro-1*H*-quinolizines containing fragments of natural alkaloids: anabasine, cytisine, methylxanthine, and colchicine. When performing the 1,3-dipolar cycloaddition of compound **2** with acetylenic derivatives of alkaloids (**4**, **6**, **8**–**10**, **14**), it proved beneficial to use a catalytic system of sodium ascorbate (15 mol%) and copper(II) sulfate (5 mol%), with an increased content of sodium ascorbate to suppress side reactions. As a result of the reaction of azide **2** with the above specified alkynes under heating in DMF to 75 °C for 5–8 h (TLC-control), the target hybrid compounds **5**, **7**, **11**–**13**, **15** were obtained in good-to-excellent yields (Scheme 1). Of interest also, was the preparation of a bis(octahydro-1*H*-quinolizine) compound **16** by the CuAAC reaction of lupinyl azide **2** with 1-((prop-2-ynyloxy)methyl)octahydro-1*H*-quinolizine **3** (yield 71%).

The synthesis of octahydro-1*H*-quinolizine derivatives having several nitrogen-substituted pharmacophores linked by the but-2-ynyloxymethyl substituent at the C-1 position was carried out by the three-component one-pot copper-catalyzed Mannich reaction of 1-(prop-2-ynyloxymethyl)octahydro-1*H*-quinolizine **3** with aq. formaldehyde and secondary amines. As can be seen, the target compounds **20**, **21**, **24**, and **25** are formed in high yields (71–84%) (Scheme 2). Thus, the reaction involving alkyne **2** with formaldehyde and various secondary amines (including the alkaloid compound) opens up the possibility for functionalization of alkaloid lupinine at the hydroxyl function.

The AChE inhibitory activity of the synthesized compounds was evaluated in comparison to the standard drug galantamine, and the results are shown in Table 1 as IC_50_ (μM) values. All tested octahydro-1*H*-quinolizine derivatives inhibited AChE at lower concentrations than the parent compound (–)-lupinine **1**. The IC_50_ values for the triazole-linked bis-alkaloids (**5**, **7**, **11**–**13**, **15**, and **16**) ranged from 0.035 to 47.0 μM. Theophylline–octahydro-1*H*-quinolizine **11** and caffeine–octahydro-1*H*-quinolizine **12** are identified as the most active inhibitors (IC_50_ = 0.47 and 0.035 μM, respectively). The activity of compound **12** was comparable with that of donepezil—a highly active and specific AChE inhibitor [50]. Compound **12**, bearing an 8-substituted caffeine moiety, is more potent than lupinine derivatives **11** and **13** derived from 7-substituted theophylline and 1-substituted theobromine. These data reveal that the direct attachment of a triazolylmethyloctahydro-1*H*-quinolizine moiety to the dimethylxanthine core could establish favorable interactions with the amino acid residues from the gorge of the AChE. In addition, attachment of the lupinine fragment at the C-1 position in analogue **13** (theobromine derivatives) decreases the enzymatic activity, in comparison with compound **11** (IC_50_ 5.8 and 0.47 μM, respectively). These data point out that the correct orientation of the xanthine improves inhibition by **11** orders of magnitude. Recently, compounds derived by chemical modifications of theophylline, theobromine, or caffeine, have been identified as inhibitors of the acetylcholinesterase enzyme [51,52,53,54,55], and all these results give valuable information about the necessary pharmacophoric chemical properties of AChE molecular targets.

Of particular interest is the inhibitory activity of the colchicine–octahydro-1*H*-quinolizine hybrid **15** against AChE (IC_50_ = 1.3 μM). Colchicine is well-known for its use in the treatment of gout. Recent studies on 10-(2-(1-benzylpiperidin-4-yl)ethylamino)colchicine have demonstrated its dual action: inhibition of cholinesterase activity and suppression of NLRP3 inflammasome activation [30]. These findings, along with our results, suggest that modification of colchicine at the C10 position is a promising strategy for the development of potential agents for the treatment of AD.

Among the synthesized bis-alkaloid hybrids, compounds **5** and **7** displayed the weakest AChE inhibitory activity (Table 1). Notably, N-(prop-2-ynyl)anabasine **4**, which served as the precursor for hybrid 5, was found to be inactive (IC_50_ = 260.0 μM).

The obtained results indicate significant inhibition of acetylcholinesterase by the bis-octahydro-1*H*-quinolizine derivative **16** (IC_50_ = 1.4 μM). These findings are comparable to those reported in [12], where a group of reversible acetylcholinesterase inhibitors—specifically, bis-lupinine derivatives featuring dicarboxylic acid ester linkers—was studied.

**Figure 3:**
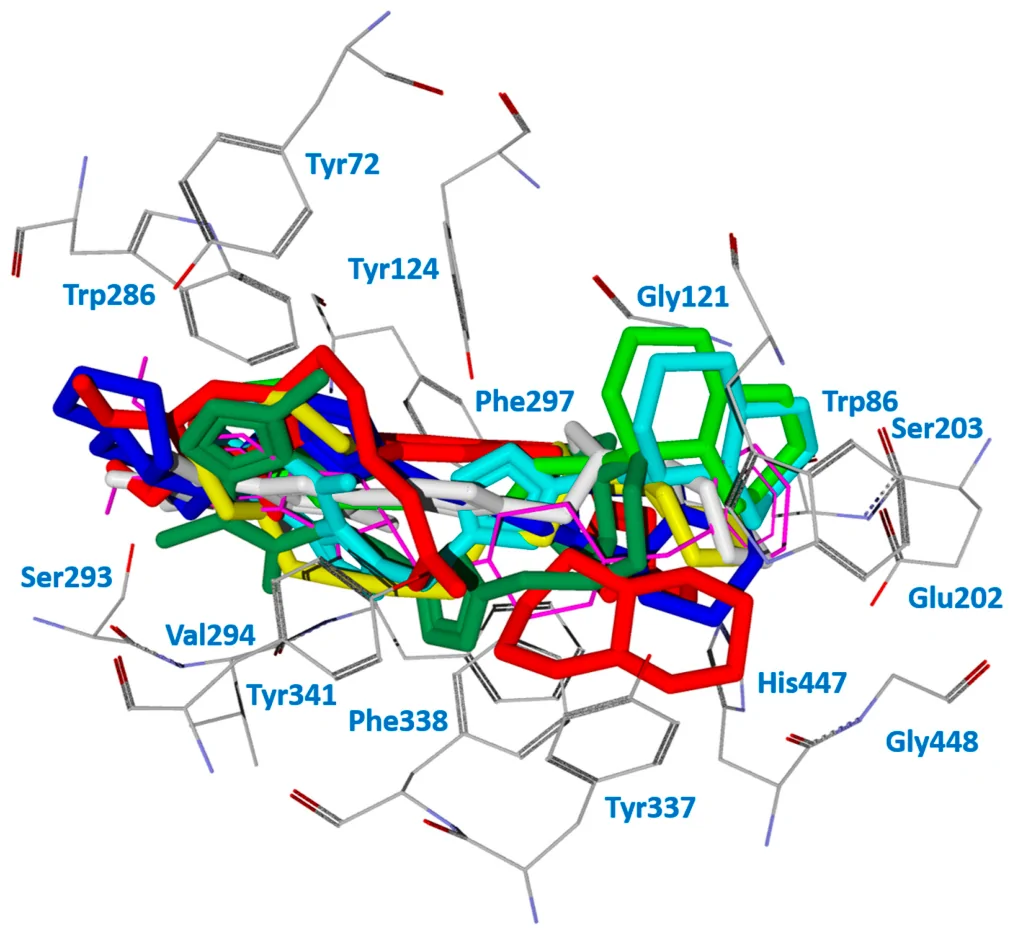
**S**uperimposed docking poses of the lupinine hybrids **11** (light-blue), **12** (gray), **13** (dark-green), **15** (red), **16** (blue), **24** (green), and **25** (yellow) in the active site of human AChE (PDB ID: 4EY7). Amino acid residues located within 3 Å of the co-crystallized ligand donepezil (pink sticks) are shown.

An observable increase in activity was detected by the two 10-(4-aminobut-2-yn-1-yloxy)lupinine derivatives **24** and **25** (IC_50_ = 3.4–4.0 μM) compared to the starting alkyne compound, 10-*O*-(prop-2-ynyl)lupinine **3** (IC_50_ = 15.0 μM). A visual inspection of the ester lupinine derivatives showed that a active compound should contain a sufficiently bulky R group for it to exhibit AChE inhibitory activity [15]. The inhibitory activity of compounds **21**, **24**, and **25** depends on the amino substituent. The presence of a piperidine or azepane cyclic moiety in the structure enhances the inhibitory activity. These results are in agreement with previously reported data indicating that the introduction of a 4-(amino)but-2-yn-1-yl fragment into the methylxanthine structure leads to an increase in acetylcholinesterase inhibitory activity, with the variation of the amino substituent having a significant impact on the activity [50]. Based on these data, molecular docking of the seven lupinine derivatives **11**, **12**, **13**, **15**, **16**, **24**, and **25** into the active site of human acetylcholinesterase (AChE; PDB ID: 4EY7, co-crystallized with donepezil) was undertaken, to interpret the measured inhibitory potencies at the atomic level, continuing our previous computational work on lupinine-based AChE inhibitors [15]. In the best-scored poses, all compounds adopted extended conformations spanning the active-site gorge (Figure 3). The lupinine fragment was invariably oriented toward the bottom of the gorge, within the subpocket bounded by Trp86, Tyr133, His447, Glu202, Ser203, Gly448, Gly120, Gly121, Ser125 and Tyr124, whereas the structurally variable opposite end of each molecule projected into the upper region framed by Ser293, Val294, Trp286, Tyr72 and Tyr341. The lower subpocket encompasses the catalytic residues Ser203 and His447, the oxyanion-domain residue Gly121 and the anionic-subsite residues Trp86, Tyr337 and Phe338, while Trp286 and Tyr124 in the upper region belong to the peripheral anionic site (PAS) at the gorge entrance [15]. Hence, analogously to donepezil, the present hybrids behave as dual-site ligands, simultaneously engaging the catalytic and peripheral subsites, reproducing the binding mode of the previously reported lupinine–triazole lead [15]. The Rosetta Interface Scores (IScores), the measured IC_50_ values, and the principal ligand–receptor interactions are presented in Table 2.

**Figure 4:**
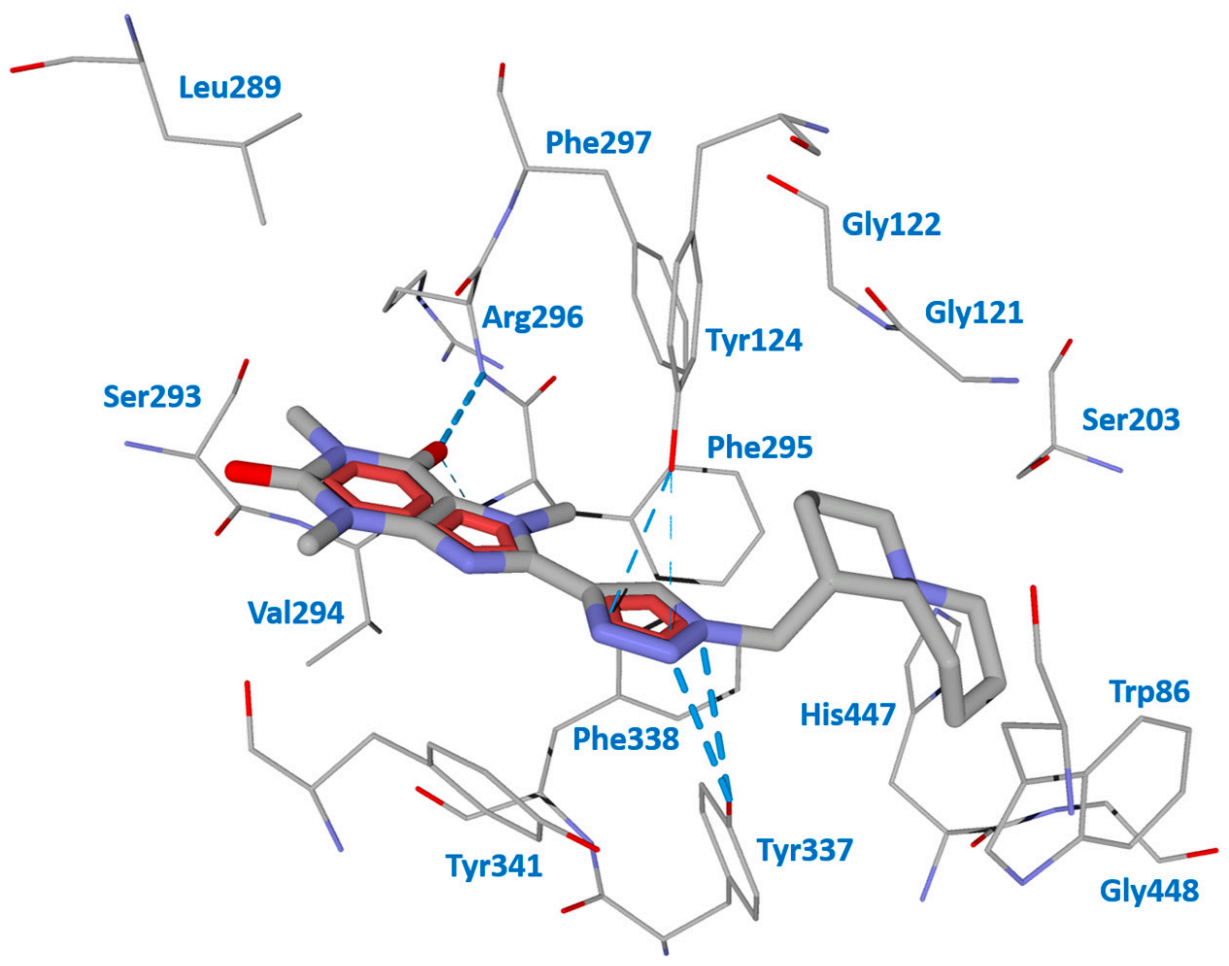
Docking pose of **12** in the AChE binding site (PDB ID: 4EY7). Amino acid residues located within 3 Å of the docked pose are shown. Hydrogen bonds are indicated by blue dashed lines.

*Lupinine–xanthine hybrids*. The point of attachment of the triazole linker to the xanthine core strongly influenced binding. Compound **12**, in which the caffeine unit is connected through its C8 position directly to the triazole ring, displayed the most favorable IScore (−20.945) and, consistently, the highest potency of the series (IC_50_ = 0.035 μM). Its docked pose (Figure 4) is stabilized by hydrogen bonds between the triazole and Tyr337/Tyr124, between a xanthine carbonyl oxygen and Arg296 and, weakly, Phe295, supplemented by van der Waals contacts with Tyr341, Trp286, Trp86, Phe338, His447, Gly121, Val294, and Phe297. The theophylline hybrid **11** preserved hydrogen bonds to Phe295 (xanthine nitrogen) and Tyr124 (triazole), whereas the theobromine hybrid **13** relied on hydrogen bonds from both xanthine carbonyls (Tyr124, Phe295) and on π–π stacking of the triazole with Tyr341 and of the xanthine with Trp286. Thus, although **13** exhibits the richest stacking pattern, the rigid, directly C8-linked caffeine architecture of **12** provides the optimal binding geometry: it is ~165-fold more potent than its N1-linked theobromine counterpart. Inhibitor **12** approaches the hAChE potency of donepezil (IC_50_ = 0.04 μM), and is about 200-fold more potent than caffeine itself (IC_50_ = 7.25 μM), a modest catalytic-site inhibitor whose enlargement in order to reach both subsites was shown to improve inhibition [56]; similar potency gains were reported upon grafting triazole-bearing arms onto caffeine (from 129 to 0.49 μM; [57] and for methylxanthine derivatives obtained by A^3^-coupling chemistry (IC_50_, down to 0.089 μM; [50]). The orientation of the xanthine toward the PAS observed here also agrees with the binding modes of caffeine-based hybrids, in which the xanthine core occupies the PAS, while a second pharmacophore anchors in the catalytic site [57,58].

*Bis-lupinine and lupinine-colchicine hybrids.* Compound **16** (IScore −17.865; IC_50_ = 1.4 μM) is held mainly by van der Waals contacts, with a single triazole–Tyr124 hydrogen bond; one quinolizidine ring occupies the deep anionic subpocket (Trp86, His447), while the second is accommodated in the upper region (Trp286, Tyr341, Tyr337, Phe338). The bis-lupinine hybrid **16** can thus be regarded as a triazole-based analogue of the bis-alkaloid derivatives of dicarboxylic acids built on lupinine, which are potent reversible cholinesterase inhibitors [59]. The colchicine hybrid **15** (IScore −18.755; IC_50_ = 1.3 μM) showed a better score in the series: its acetamide NH forms a hydrogen bond with Asp74, anchoring the colchicine end at the rim of the upper gorge, while the trimethoxyphenyl–tropolone system establishes multiple aromatic contacts (Trp286, Phe338, Tyr341, Tyr337) and the lupinine end contacts Trp86, His447 and Gly121. This arrangement agrees with the docking of colchicine–aryl/alkyl amine hybrids, in which the hydrophobic trimethoxyphenyl framework of colchicine preferentially occupies the PAS [30]; fusion with the lupinine anchor evidently improves AChE complementarity, relative to colchicine alkaloids alone.

*Acetylene-type amine hybrids*. Compounds **24** (IScore −16.696; IC_50_ = 4.2) and **25** (IScore −15.817; IC_50_ = 3.4 ± 0.1 μM), which carry a small saturated piperidine or azocane ring instead of a heteroaromatic terminus, and which lack the triazole, exhibited the least favorable scores and the weakest activities of the series. Compound **24** forms a single hydrogen bond (Tyr124 with the ether oxygen), whereas **25** is bound exclusively through van der Waals contacts. Because these heterocyclic rings cannot engage the aromatic residues of the upper gorge as effectively as the planar xanthine or colchicine systems, these results indirectly highlight the contribution of the triazole and of the heteroaromatic termini to binding [33].

Correlation between docking scores and activity. The pIC_50_ values correlated satisfactorily with the IScores (r = −0.82, *n* = 7, *F* = 10.37, *p* = 0.023): more favorable (more negative) scores corresponded to higher potency, and the best-scoring compound **12** was, indeed, the most active one (Table 2). A score–activity correlation of comparable strength (r = 0.800 for an external test set) was reported for galantamine derivatives docked into AChE [60], supporting the quantitative plausibility of the present model. At the same time, the Interface Score reflects the complementarity of a single best pose, and does not capture desolvation, conformational entropy, protonation equilibria of the basic nitrogen atoms, or assay-medium effects; consequently, minor inversions—such as that between **24** and **25**—are to be expected, and the score should be regarded as a within-series ranking tool, rather than an absolute affinity predictor.

All the obtained data revealed that the lupinine derivatives reported here could be used for further biological evaluation in cell culture and with in vivo models.

## 4. Materials and Methods 

### 4.1. General Information

Melting points were determined on the Mettler Toledo FP900 thermosystem (Columbus, OH, USA). NMR spectra were acquired on Bruker Avance-400 (^1^H: 400.13 MHz, ^13^C: 100.62 MHz) and Bruker DRX 500 (^1^H: 500.13 MHz, ^13^C: 125.76 MHz) spectrometers (Bruker Corporation, Karlsruhe, Germany). Deuterochloroform (CDCl_3_) was used as a solvent and the residual CHCl_3_ (δ_H_ = 7.26 ppm) and CDCl_3_ (δ_C_ = 77.0 ppm) were employed as internal standards. NMR signal assignments were carried out with the aid of a combination of 1D and 2D NMR techniques that included ^1^H, ^13^C, COSY, HSQC, and HMBC spectra. In the description of the ^1^H and ^13^C-NMR spectra for all compounds, the atom numbering system given in Scheme 1 and Scheme 2 was used. IR absorption spectra were recorded on a Bruker Vector 22 FT-IR spectrometer (Bruker Corporation, Karlsruhe, Germany) in KBr pellets. Optical rotation values ([α]_D_^t^) were registered on PolAAr 3005 (Optical Activity Ltd., Canbridgeshire, UK). Mass spectra were recorded on a DFS Thermo Scientific high-resolution mass spectrometer (evaporator temperature 120–280 °C, electron impact ionization at 70 eV) (Thermo Fisher Scientific, Waltham, MA, USA). The X-ray diffraction experiments for compound **5** were carried out at 296(2) K on a Bruker KAPPA APEX II CCD diffractometer (graphite-monochromated Mo Kα radiation). Reflection intensities were corrected for absorption by the multi-scan method the SAINT [61] and SADABS [62] programs included in the APEX2 package [63]. The structure of compound **5** was solved by direct methods using SHELXS-2014/5 [64] and refined by the anisotropic (isotropic for all H atoms) full-matrix least-squares method, against *F^2^* of all reflections, by SHELXL2018/3 [65]. Crystallographic data for the structure have been deposited at the Cambridge Crystallographic Data Centre as as Supplementary Publication no. CCDC 2562170. These data can be obtained free of charge from The Cambridge Crystallographic Data Centre via www.ccdc.cam.ac.uk/data_request/cif (accessed on 15 June 2026).

The reaction progress was controlled by TLC on Sorbfil UV-254 plates using CHCl_3_-EtOH, 10:1, or petroleum ether-EtOAc, 3:1; detection was under UV light or by treatment with iodine vapor. Column chromatography was performed using silica gel 60 (0.063–0.200 mm, Merck KGaA, Darmstadt, Germany).

The chemicals used were as follows: (–)-Lupinine (**1**) (m.p. 69–71 °C (EtOH), [α]_D_^25^ = −30.5° (*c* 0.41, MeOH); (literature data: mp 68–69 °C (EtOH), [α]_D_^25^ = −23.52° (EtOH) [66], [α]_D_^26^ = −25.93° (c 3.18, H_2_O [67]) was isolated from the *Anabasis aphyla* L., as described in [67]. Lupinine azide (**2**) [43] N-(prop-2-ynyl)cytisine (**6**) [68], 8-ethynylcaffeine (**9**) [50,69], 7-(prop-2-yn-1-yl)-1,3-dimethyl-3,7-dihydro-1*H*-purine-2,6-dione (**8**) [50,70], 1-(prop-2-yn-1-yl)-3,7-dimethylxanthine (**10**) [50,70] and 10-(prop-2ynylamino)-10-nor-colchicine (**14**) [71] were obtained in accordance with methods described earlier. Anabasine (for preparation of the compound **4**) was isolated from the plant *Anabasis aphylla* L., according to a known procedure [72], [*α*]_D_^23^ = −72.3 (c 5.2, CHCl_3_). The chemicals usedwere propargyl bromide (80% solution in toluene) **17**, formalin (30% formaldehyde in aq. solution), NaH (60% in mineral oil), diisopropylamine **18**, dibutylamine **19**, piperidine **22**, azepane **23**, Cu(OAc)_2_·H_2_O, CuSO_4_·5H_2_O, and sodium ascorbate, Ellman’s reagents, were purchased from Aldrich (St. Louis, MO, USA) or Alfa Aesar (GmbH, Karlsruhe, Germany). Solvents (DMF, DMSO, 1,4-dioxane, CHCl_3_, CH_2_Cl_2_) were purified by standard methods.

### 4.2. Synthetic Methods 

Synthesis of(1*R*,9a*R*)-1-((prop-2-ynyloxy)methyl)octahydro-1*H*-quinolizine (**3**). To a stirred solution of lupinine **1** (1.69 g, 10 mmol) in DMSO (20 mL) cooled to 10 °C, sodium hydride (60% dispersion in mineral oil, 0.8 g, 20 mmol) was added portion-wise, under an argon atmosphere. After 30 min. of stirring at 10 °C, an 80% solution of propargyl bromide in toluene **17** (2 g, 14 mmol) was added portion-wise. The mixture was stirred at room temperature for 10 h, diluted with saturated aq. sodium chloride, and extracted with EtOAc (3 × 30 mL). The combined extracts were washed with brine, dried over MgSO_4_, evaporated, and purified by silica-gel column chromatography (eluent: chloroform). Yellowish oil; Yield 45% (0.95 g); [*α*]_D_^20^ = −22.8 (*c* 1.04, CHCl_3_). IR (KBr) ν: 3310 (C≡CH), 2933, 2858, 2759 (quinolizine), 2113 (C≡C), 1097 (C-O) cm^–1^; ^1^H NMR (CDCl_3_, 500 MHz) δ: 1.15–1.26 (1H, m, H-2_a_), 1.33 (1H, dd, *J* = 13.0, 4.2 Hz, H-9_a_), 1.38–1.57 (5H, m, H-3_a_,7_a_, 8_a_,7_e_,8_e_), 1.62–1.75 (2H, m, H-2_e_,9_e_), 1.81 (1H, m, H-3_e_), 1.84–2.04 (4H, m, H-1_a_,9_a_,4_a_,6_a_), 2.38 (1H, t, *J* = 2.4 Hz, H-13), 2.74–2.83 (2H, m, H-4_e_,6_e_), 3.59 (1H, dd, *J* = 10.0, 8.5 Hz, H-10), 3.72 (1H, dd, *J* = 10.0, 5.4 Hz, H-10), 4.09 (1H, d, *J* = 2.4 Hz, H-12) ppm; ^13^C NMR (CDCl_3,_ 125 MHz) δ: 21.06 (C-3), 24.95 (C-8), 25.32 (C-7), 26.92 (C-2), 29.75 (C-9), 38.49 (C-1), 56.94 (C-4(6)), 57.32 (C-6(4)), 58.12 (C-11), 64.52 (C-9a), 69.05 (C-10), 74.6 (C-13), 79.98 (C-12) ppm; HR-MS, *m*/*z* (*I*_rel_, %): 207 (4), 206 (11, [M]^+^), 168 (33), 153(29), 152 (80), 138 (20), 124 (100), 110 (29), 98 (38), 97 (25), 96 (33), 83 (41); Calcd for: C_13_H_20_NO: 206.1539; found, *m*/*z*: 206.1538 [M]^+^.

Synthesis of (*S*)-3-(1-(prop-2-ynyl)piperidin-2-yl)pyridine [(*S*)-N-(prop-2-ynyl)anabasine] (**4**). To a stirred solution of anabasine (2.0 g, 12.33 mmol) in acetone (50 mL) was added K_2_CO_3_ (5.11 g, 36.98 mmol), followed by a solution of propargyl bromide **17** (1.87 mL, 24.66 mmol) in acetone (5 mL) at 5–10 °C. The reaction mixture was stirred at room temperature for 8 h, diluted with brine (100 mL), and extracted with chloroform (3 × 70 mL). The combined organic layers were washed with brine (50 mL), dried over magnesium sulfate, and filtered. The solvent was removed under reduced pressure, and the residue was purified by column chromatography (eluting with a chloroform–ethanol mixture, 100:1 → 20:1) to afford compound **4** as white crystals. Yield 78% (1.56g); mp 84–85 °C; [α]_D_^21^ = −296.6 (*c* 0.6, CHCl_3_); ^1^H NMR (CDCl_3_, 400 MHz) δ: 1.28–1.34 (1H, m, H-5_a_), 1.38–1.44 (1H, m, H-4_a_), 1.49–1.65 (3H, m, H-3_a_,4_e_,5_e_), 1.70–1.80 (1H, m, H-3_e_), 2.20 (1H, s, H-9), 2.68 (1H, dd, *J* = 12.4, 7.9 Hz, H-6_a_), 2.93 (1H, dd, *J* = 12.4, 2.8 Hz, H-6_e_), 3.03 (1H, d, *J* = 15.9 Hz, H-7), 3.14 (1H, d, *J* = 15.9 Hz, H-7), 3.31 (1H, dd, *J* = 11.9, 2.9 Hz, H-2_a_), 7.23 (1H, dd, *J* = 6.8, 7.6 Hz, H-5′), 7.68 (1H, dd, *J* = 7.6, 1.4 Hz, H-4′), 8.49 (1H, d, *J* = 6.8 Hz, H-6′), 8.66 (1H, d, *J* = 1.4 Hz H-2′) ppm; ^13^C NMR (CDCl_3_, 101 MHz) δ: 24.46 (C-4), 25.66 (C-3), 35.46 (C-5), 44.04 (C-7), 52.86 (C-6), 62.87 (C-2), 73.64 (C-9), 77.74 (C-10), 123.57 (C-5′), 134.31 (C-4′), 138.75 (C-3′), 148.74 (C-2′), 149.42 (C-6′) ppm; HR-MS, *m*/*z* (*I*_rel_, %): 201 (6), 200 (35, [M]^+^), 122 (100), 99 (29); Calcd for C_13_H_16_N_2_: 200.1308; found *m*/*z* 200.1307 [M]^+^.

#### 4.2.1. General Procedure for Synthesis of Triazol-Linked Bis-Alkaloid Derivatives **5**, **7**, **11**–**13**, **15** and **16**

A mixture of lupinine azide (**2**) (0.29 g, 1.5 mmol), substituted alkynes [N-(prop-2-ynyl)anabasine (**4**), N-(prop-2-ynyl)cytisine (**6**), 7-(prop-2-yn-1-yl)-1,3-dimethyl-3,7-dihydro-1*H*-purine-2,6-dione (**8**), 8-ethynylcaffeine (**9**), 1-(prop-2-yn-1-yl)-3,7-dimethylxanthine (**10**), 10-(prop-2-ynylamino)-10-nor-colchicine (**14**), and 1-((prop-2-ynyloxy)methyl)octahydro-1*H*-quinolizine (**3**)] (1.35 mmol), CuSO_4_·5H_2_O (0.017 g, 0.0675 mmol) and sodium ascorbate (0.039 g, 0.2025 mmol) in DMF (20 mL) was stirred at 75 °C for 5–8 h, using TLC monitoring. After cooling, the residue was filtered, the precipitate was washed with CHCl_3_ (50 mL), the combined organic extract was evaporated, and the residue was purified by column chromatography (eluting with a chloroform–ethanol mixture, 100:1 → 10:1) to afford compounds **5**, **7**, **11**–**13**, **15**, and **16**.

(1*S*,9a*R*)-1-((4-(((S)-2-(pyridin-3-yl)piperidin-1-yl)methyl)-1*H*-1,2,3-triazol-1-yl)methyl)octahydro-1*H*-quinolizine (**5**). Yellowish crystals; yield 86%; mp 109–112 °C, [α]_D_^20^ = −65.9 (*c* 0.7, CHCl_3_); IR (KBr) ν: 2933, 2854, 2750 (quinolizine), 1625 (C=C), 1589, 1577 (C=N) cm^–1^; ^1^H NMR (CDCl_3_, 400 MHz) δ: 1.15–1.37 (4H, m, H-2_a_,9_a_,5′,6′); 1.38–1.48 (2H, m, H-3_a_,7_a_), 1.49–1.63 (5H, m, H-2_e_,7_e_,8_e_,8_a_,3_e_), 1.64–1.88 (5H, _M_, H-9_e_,3′,5′,4′,3′), 1.89–2.12 (3H, m, H-1,4_a_,6_a_), 2.13–2.26 (2H, m, H-9_a_,6′), 2.79–2.95 (2H, m, H-4_e_,6_e_), 3.02–3.11 (m, 1H, H-6′), 3.16 (1H, dd, *J* = 11, 2.4 Hz, H-2′), 3.32 (1H, d, *J* = 14.3 Hz, CH_2_), 3.67 (1H, d, *J* = 14.3 Hz, CH2), 4.49 (1H, dd, *J* = 14.1, 5.5 Hz, H-10), 4.58 (1H, dd, *J* = 14.1, 10.3 Hz, H-10), 7.27 (1H, s, H-5, triazole), 7.30 (1H, dd, *J* = 6.8, 7.9 Hz, H-5″), 7.77 (1H, dd, *J* = 7.9, 1.6 Hz, H-6″), 8.51 (1H, dd, *J* = 6.8, 1.6 Hz, H-4″), 8.61 (1H, d, *J* = 1.6 Hz, H-2″) ppm; ^13^C NMR (CDCl_3,_ 125 MHz) δ: 20.39 (C-3), 24.65 (C-4′), 24.70 (C-8), 25.38 (C-7), 25.63 (C-5′), 26.11 (C-2), 29.65 (C-9), 36.67 (C-3′), 39.05 (C-1), 48.18 (C-10), 49.90 (C-14), 53.11 (C-13), 56.91 (C-4(6)), 57.14 (C-6(4)), 64.16 (C-9a), 65.16 (C-2′), 122.65 (C-5-triazole), 123.57 (C-5′’), 135.03 (C-6″), 140.20 (C-4-triazole), 144.26 (C-3″), 148.48 (C4″), 149.19 (C2″) ppm; HR-MS, *m*/*z* (*I*_rel_, %): 396 (0.4), 395 (1.4), 394 (1.6, [M]^+^), 234 (21), 206 (12), 162 (24), 161 (100), 152 (46), 151 (24), 150 (19), 133 (18), 106 (18), 105 (23), 84 (38); Calcd for C_23_H_34_N_6_: 394.2840; found *m*/*z* 394.2836 [M]^+^.

Crystal data **5**: C_23_H_34_N_6_, M = 394.56, crystal size 0.680 × 0.180 × 0.060 mm^3^; orthorhombic, space group P 2_1_ 2_1_ 2_1_, *a* = 17.5198(5), *b* = 27.842(3), *c* = 5.5235(4) Å, α = 90 (3), β = 90 (2), γ = 90 (2)°, *V* = 2694.2(4) Å^3^, *Z* = 4, d_calc_ = 0.973 g·cm^−3^, µ = 0.060 mm^−1^, a total of 36,707 (θ range for data collection 3.149 to 25.088°; completeness θ_max_ = 20.273° – 99.2%), 4770 unique (*R*_int_ = 0.0787), 4770 [*I* > 2σ(*I*)], 262 parameters. GooF = 2.515, *R*_1_ = 0.1005, *wR*_2_ = 0.2556 [*I* > 2σ(*I*)], *R*_1_ = 0.0852, *wR*_2_ = 0.2437 (all data), max/min diff. peak 2.408 and −0.756 e·Å^−3^; absolute structure parameter: −1.7(9).

(1*R*,5*S*)-3-(1-(((1*S*,9a*R*)-octahydro-1*H*-quinolizin-1-yl)methyl)-1*H*-1,2,3-triazol-4-yl)methyl-1,2,3,4,5,6-hexahydro-8*H*-1,5-methanopyrido[1,2-*a*][1,5]diazocine-8-one (**7**). White amorphous solid; yield 88%; [α]_D_^22^ = −106.0 (*c* 0.5, CHCl_3_); IR (KBr) ν: 2935, 2854, 2777 (quinolizine), 1645 (C=O), 1618, 1548 (C=C, C=N) cm^–1^; ^1^H NMR (CDCl_3_, 400 MHz) δ: 1.03–1.12 (1H, m, H-2_a_), 1.23–1.33 (2H, m, H-8_a_,9_a_), 1.40–1.61 (5H, m, H-2_e_,3_e_,7_e_,3_a_,7_a_), 1.70–1.88 (4H, _M_, 4_a_,6_a_,8_e_,9_e_), 2.00–2.05 (m, 1H, H-12′), 2.06–2.19 (2H, m, H-1,9_a_), 2.32–2.44 (3H, m, H-1′,5′,12′), 2.75–2.93 (6H, m, H-4_e_,6_e_,2H-2′,2H-4′), 3.53 (2H, c, CH_2_), 3.80 (1H, dd, *J* = 15.4, 7.0 Hz, H-6′), 4.00 (1H, d, J = 15.4 Hz, H-6′), 4.41 (1H, dd, *J* = 14.2, 5.0 Hz, H-10), 4.48 (1H, dd, *J* =14.2, 10.8 Hz, H-10), 5.88 (1H, dd, *J* = 7.2, 1.4 Hz, H-11′), 6.41 (1H, dd, J = 8.6, 1.4 Hz, H-9′), 6.94 (1H, c, H-5-triazole), 7.23 (1H, dd, J = 8.6, 7.2, H-10′) ppm; ^13^C NMR (CDCl_3,_ 125 MHz) δ: 20.12 (C-3), 24.45 (C-8), 24.98 (C-7), 25.54 (C-12′), 25.72 (C-2), 27.82 (C-5′), 29.41 (C-9), 35.24 (C-1′), 38.89 (C-1), 38.45 (C-13), 48.40 (C-10), 49.88 (C-6′); 52.97 (C-6′), 56.58, 57.00 (C-4,2′), 59.72, 59.78 (C-6,4′), 64.13 (C-9a), 104.48 (C-11′), 116.39 (C-9), 122.57 (C-5-triazole), 138.64 (C-10′), 144.95 (C-4-triazole), 151.46 (C-11a′), 163.49 (C-8′) ppm; HR-MS, *m*/*z* (*I*_rel_, %): 425 (3), 424 (13), 423 (4), 422 (8, [M]^+^), 152 (30), 151 (100), 150 (15), 138 (14), 136 (12); Calcd for C_24_H_34_N_6_O: 422.2789; found *m*/*z* 422.2790 [M]^+^.

1,3-Dimethyl-7-((1-(((1*S*,9a*R*)-octahydro-1*H*-quinolizin-1-yl)methyl)-1*H*-1,2,3-triazol-4-yl)methyl)-1*H*-purine-2,6(3*H*,7*H*)-dione (**11**). White solid; yield 87%; mp 162–164 °C; [α]_D_^20^ = −17.5 (*c* 0.7, CHCl_3_). IR (KBr) ν: 2935, 2863, 2802 (quinolizine), 1700 (C=O), 1645 (C=O), 1604, 1550 (C=C, C=N) cm^–1^; ^1^H NMR (CDCl_3_, 500 MHz) δ: 1.11–1.36 (3H, m, H-2_e_,2_a_,8_a_), 1.39–1.64 (5H, m, H-7_e_,8_e_,3_a_,7_a_,9a), 1.71–1.88 (2H, _M_, H-3_e_,9_e_), 1.89–2.05 (2H, m, H4_a_,6_a_), 2.05–2.13 (1H, m, H-1), 2.15–2.26 (1H, m, H-9_a_), 2.80–2.93 (2H, m, H-4_e_,6_e_), 3.37 (3H, s, CH_3_-N-3′), 3.53 (3H, s, CH_3_-N-1′), 4.49 (1H, dd, *J* = 14.0, 5.2 Hz, H-10), 4.59 (1H, dd, *J* = 14.0, 10.8 Hz, C-10), 5.54 (2H, s, CH_2_), 7.77 (1H, s, H-8′), 7.78 (1H, c, H-5-triazole) ppm; ^13^C NMR (CDCl_3_, 125 MHz) δ: 20.22 (C-3), 24.53 (C-8), 25.22 (C-2), 25.95 (C-7), 27.86 (CH_3_), 29.56 (C-9), 29.68 (CH_3_), 38.88 (C-1), 41.42 (CH_2_), 48.51 (C-10), 56.69 (C-4), 57.01 (C-6), 64.11 (C-9a), 106.31 (C-5′), 124.11 (C-5-triazole), 141.16 (C-8′), 141.70 (C-4-triazole), 148.82 (C-6′), 151.47 (C-2′), 155.25 (C-4′) ppm; HR-MS, *m*/*z* (*I*_rel_, %): 413 (5), 412 (16, [M]^+^), 411 (2), 180 (12), 152 (41), 151 (100), 150 (60), 136 (48), 122 (19), 111 (17), 110 (20), 98 (19), 96 (23), 83 (36); calcd for C_20_H_28_N_8_O_2_: 412.2330; found *m*/*z* 412.2334 [M]^+^.

1,3,7-Trimethyl-8-(1-(((1*S*,9a*R*)-octahydro-1*H*-quinolizin-1-yl)methyl)-1*H*-1,2,3-triazol-4-yl)-1*H*-purine-2,6(3*H*,7*H*)-dione (**12**). White solid; yield 85%; mp 238–239 °C; [α]_D_^20^ = −22.2 (*c* 0.4, CHCl_3_). IR (KBr) ν: 2939, 2860, 2804 (quinolizine), 1703 (C=O), 1662 (C=O), 1608, 1545 (C=C, C=N) cm^–1^; ^1^H NMR (CDCl_3_, 400 MHz) δ: 1.18–1.32 (2H, m, 2_a_,8_a_), 1.37 (1H, dd, *J* = 13.2, 11.2 Hz, H-9_a_), 1.43–1.64 (5H, m, H-2_e_,7_a_,8_e_,3_a_,7_a_), 1.73–1.81 (1H, m, H-3_e_), 1.87 (1H, dd, *J* = 13.2, 4.0 Hz, H-9_e_), 1.91–2.04 (2H, m, H-4_a_,6_a_), 2.06–2.14 (1H, m, H-1), 2.21–2.30 (1H, m, H-9a), 2.80–2.90 (2H, m, H-4_e_,6_e_), 3.37 (3H, s, CH_3_-N-1′), 3.54 (3H, s, CH_3_-N-3’), 4.41 (3H, s, CH_3_-N-7′), 4.60 (1H, dd, *J* = 14.1, 5.2 Hz, H-10), 4.70 (1H, dd, J =14.1, 11.2 Hz, H-10), 8.11 (1H, s, H-5-triazole) ppm; ^13^C NMR (CDCl_3_, 125 MHz) δ: 20.25 (C-3), 24.58 (C-8), 25.29 (C-7), 26.05 (C-2), 27.83 (CH_3_-N-7′), 29.57 (C-1), 29.71 (C-9), 34.24 (CH_3_-N-1′), 39.05 (CH_3_-N-3′), 48.70 (C-10), 56.80 (C-4), 57.06 (C-6), 64.07 (C-9a), 108.22 (C-5′), 125.39 (C-5-triazole), 139.18 (C-8′), 142.86 (C-4-triazole), 148.07 (C-4′), 151.58 (C-2′), 155.21 (C-6′) ppm; HR-MS, *m*/*z* (*I*_rel_, %): 414 (3), 413 (25), 412 (95, [M]^+^), 278 (13), 277 (17), 152 (25), 151 (100), 150 (49), 136 (14); calcd for C_20_H_28_N_8_O_2_: 412.2330; found *m*/*z* 412.2329 [M]^+^.

3,7-Dimethyl-1-((1-(((1*S*,9a*R*)-octahydro-1*H*-quinolizin-1-yl)methyl)-1*H*-1,2,3-triazol-4-yl)methyl)-1*H*-purine-2,6(3*H*,7*H*)-dione (**13**). White solid; yield 90%; mp 138–140 °C; [α]_D_^20^ = −12.2 (*c* 0.5, CHCl_3_); IR (KBr) ν: 2935, 2856, 2802 (quinolizine), 1709 (C=O), 1658 (C=O), 1604, 1548 (C=C, C=N) cm^–1^; ^1^H NMR (CDCl_3_, 400 MHz) δ: 1.05–1.23 (3H, m, H-2_e_,8_e_,2_a_), 1.25–1.49 (5H, m, H-7_e_,8_e_,3_a_,7_a_,9_a_), 1.58–1.66 (1H, m, H-3_e_), 1.72 (1H, dd, J = 13.2, 4.2 Hz, H-9_e_), 1.80–1.92 (2H, m, H-1,9a), 1.93–2.09 (2H, m, H-4_a_,6_a_), 2.67–2.75 (2H, m, 4_e_,6_e_), 3.39 (3H, s, CH_3_-N-3′), 3.84 (3H, s, CH_3_-N-7′), 4.33 (1H, dd, *J* = 14.2, 5.2 Hz, H-10), 4.42 (1H, dd, *J* = 14.2, 10.4 Hz, H-10), 5.13 (2H, m, CH_2_), 7.44 (1H, s, H-5-triazole), 7.46 (1H, s, H-8′) ppm; ^13^C NMR (CDCl_3_, 125 MHz) δ: 19.93 (C-3), 24.23 (C-7), 24.77 (C-8), 25.57 (C-2), 28.99 (C-9), 29.28 (CH3-N-3′), 33.19 (C-1), 35.54 (CH_2_), 38.62 (CH_3_-N-7′), 47.90 (C-10), 56.30 (C-4(6)), 56.70 (C-6(4)), 63.72 (C-9a), 107.07 (C-5′), 123.38 (C-5-triazole), 141.45 (C-8′), 142.86 (C-4-triazole), 148.4 (C-4′), 150.82 (C-2′), 154.27 (C-6′) ppm; HR-MS, *m*/*z* (*I*_rel_, %): 413 (3), 412 (13, [M]^+^), 261 (13), 152 (26), 151 (100), 150 (36), 136 (26), 111 (15), 83 (21); calcd for C_20_H_28_N_8_O_2_: 412.2330; found 412.2326 [M]^+^.

(1*R*,5*S*)-10-(1-(((1*S*,9a*R*)-octahydro-1*H*-quinolizin-1-yl)methyl)-1*H*-1,2,3-triazol-4-yl)methylamino-N-[(7*S*)-acetylamino-1,2,3,-trimethoxy-6,7-dihydro-5*H*-benzo[*a*]heptalen-9-one (**15**). White solid; yield 72%; mp 208–210 °C; [α]_D_^20^ = −87.2 (*c* 1.2, CHCl_3_); IR (KBr) ν: 2935, 2866, 2802 (quinolizine), 1700 (C=O), 1666 (C=O), 1604, 1548 (C=C, C=N) cm^–1^; ^1^H NMR (CDCl_3_, 400 MHz) δ: 1.23–1.42 (4H, m, H-2_a_,3_a_,2_e_,8_a_), 1.47–1.65 (5H, m, H-3_e_,7_a_,8_e_,7_e_,9_a_), 1.73–1.90 (4H, _M_, H-4_a_,6_a_,9_e_,6′), 1.95 (c, 3H, CH_3_C=O), 2.03–2.12 (1H, m, H-1), 2.19–2.37 (3H, m, H-9a,6′,5′), 2.44 (1H, dd, J = 12.8, 5.9 Hz, H-7′), 2.86–3.00 (2H, m, H-4_e_,6_e_), 3.57 (3H, s, OCH_3_-C-2′), 3.86 (3H, s, OCH_3_-C-3′), 3.90 (3H, s, OCH_3_-C-1′), 4.53– 4.67 (3H, m, H-10,7′,10), 4.69 (2H, d, J = 5.6 Hz, CH_2_NH), 6.49 (1H, s, H-4′), 6.66 (1H, d, *J* = 11.2 Hz, H-11′), 7.37 (1H, d, *J* = 11.2 Hz, H-12′), 7.45 (1H, s, H-5-triazole), 7.51 (1H, s, H-8′), 7.49–7.54 (1H, m, NH-CH_2_), 7.63 (1H, d, *J* = 5.8 Hz, NH-Ac) ppm; ^13^C NMR (CDCl_3_, 125 MHz) δ: 19.75 (C-3), 22.61 (CH_3_-Ac), 23.83 (C-8), 24.29 (C-7), 25.36 (C-2), 28.80 (C-9), 29.78 (C-5′), 36.72 (C-6′), 38.31 (C-1), 38.45 (CH_2_), 48.40 (C-10), 55.87 (OCH_3_-C-2′), 56.65 (C-4,6), 61.11 (OCH_3_), 61.19 (OCH_3_), 64.25 (C-9a), 106.86 (C-4′), 108.65 (C-11′), 122.66 (C-5-triazole), 123.56 (C-8′), 126.39 (C-12b′), 131.08 (C-4a′), 134.37 (C-12a′), 138.84 (C-12′), 141.11 (C-7a′), 143.32 (C-4-triazole), 150.74 (C-2′), 151.43 (C-10′), 152.65 (C-3′), 153.32 C-1′), 169.82 (C=O-Ac), 175.14 (C-9′) ppm; HR-MS, *m*/*z* (*I*_rel_, %): 618 (6), 617 (8), 616 (31, [M]^+^), 385 (25), 384 (30), 309 (24), 282 (20), 263 (21), 262 (100), 203 (75), 190 (37), 152 (33), 151 (65), 150 (25), 136 (37), 133 (42), 119 (31); calcd for C_34_H_44_N_6_O_5_: 616.3368; found *m*/*z*: 616.3367 [M]^+^.

(1*S*,9a*R*)-1-((4-((((1*S*,9a*S*)-octahydro-1*H*-quinolizin-1-yl)methoxy)methyl)-1*H*-1,2,3-triazol-1-yl)methyl)octahydro-1*H*-quinolizine (**16**). Yellowish oil; yield 71%; [α]_D_^20^ = −18.6 (*c* 0.9, CHCl_3_); IR (KBr) ν: 2933, 2858, 2800, 2761 (quinolizine), 1604, 1548 (C=C, C=N), 1110, 1103, 1051 (C-O-C) cm^–1^; ^1^H NMR (CDCl_3_, 300 MHz) δ: 1.15–1.33 (4H, m, H-2_a_,2′_a_,9_a_,9′_a_), 1.34–1.65 (11H, m, 3_a_,3′_a_,7_a_,7′_a_,8_a_,8′_a_,3_e_,3′e,7_e_,7′_e_,8_e_), 1.66–1.90 (6H, m, 1′,2_e_,2′_e_,8′_e_,9_e_,9′_e_); 1.91–2.42 (7H, m, H-1,9a,9a′,4_a_,4′_a_,6_a_,6′_a_), 2.77–3.13 (4H, m, 4_e_,4′_e_,6_e_,6′_e_), 3.62 (br s, 2H, H-10), 4.59 (1H, d, J = 10.2 Hz, 1H, H-10′), 4.53 (2H, c, CH_2_O), 4.59 (1H, d, H-10′), 7.57 (1H, c, H-5-triazole) ppm; ^13^C NMR (CDCl_3_, 125 MHz) δ: 20.24 (C-3,3’), 23.91 (C-8,8’), 24.49 (C-7(7′)), 25.11 (C-2,2′), 25.86 (C-7′(7)), 29.49 (C-9,9′), 37.78 (C-1(1′)), 38.86 (C1′(1)), 48.23 (C-10), 56.69 (C-4,4′), 57.02 (C-6,6′), 64.07 (C-9a,9′a), 64.23 (C-10′), 68.98 (CH_2_-O), 123.25 (C-11), 144.60 (C-12) ppm; HR-MS, *m*/*z* (*I*_rel_, %): 402 (3), 401 (12.6, [M]^+^), 169 (13), 168 (100), 153 (80), 152 (69), 151 (18), 138 (21), 85 (21), 83 (38), 45(26); calcd for C_23_H_39_N_5_O: 401.3149; found *m*/*z* 401.3150 [M]^+^.

#### 4.2.2. General Procedure for A^3^-Coupling Reaction; Synthesis of Lupinine Derivatives **20**, **21**, **24**, and **25**

Secondary amines diisopropylamine **18**, dibutylamine **19**, piperidine **22**, or azepane **23** (5.2 mmol), aq. formaldehyde (60 mg, 20 mmol), and Cu(OAc)_2_·H_2_O (0.75 mL, 0.5 mmol) were successively added in argon to the solution of 1-(prop-2-ynyloxymethyl)octahydro-1*H*-quinolizine **3** (0.61g, 5 mmol) in dioxane (10 mL), and the reaction mixture was stirred at 65–70 °C for a period of 5 to 8 h (TLC-control). After the consumption of the starting materials, the reaction mixture was cooled, and evaporated in vacuo. The residue was dissolved in EtOAc (150–200 mL), washed with brine, dried over MgSO_4_, filtered, and evaporated. The oily residue was dissolved in a minimum amount of CHCl_3_ and separated by silica-gel column chromatography (eluent CHCl_3_–EtOH, gradient 100:0.5 → 10:1).

N,N-Diisopropyl-4-(((1*R*,9a*R*)-octahydro-1H-quinolizin-1-yl)methoxy)but-2-yn-1-amine (**20**). Yellowish oil; yield 77%; ^1^H NMR (CDCl_3_, 300 MHz) δ: 1.06 (12H, d, *J* = 6.5 Hz, 4 × CH_3_-*i*-*Pr*), 1.12–1.24 (1H, m, H-2_a_) 1.25–1.35 (1H, m, H-9_a_), 1.36–1.57 (5H, m, 3_a_,7_a_,8_a_,7_e_,8_e_), 1.59–1.81 (3H, m, 2_e_,3_e_,9_e_), 1.82–2.04 (4H, m, 1,9a,4_a_,6_a_), 2.73–2.83 (2H, m, H-2_e_,6_e_), 3.08–3.24 (2H, m, 2-CH-*i*-*Pr*), 3.41 (2H, m, H-15), 3.56 (1H, dd, *J* = 10.6, 8.3 Hz, H-10), 3.72 (1H, dd, *J* = 10.6, 5.2 Hz, H-10), 4.09 (2H, m, H-12) ppm; ^13^C NMR (CDCl_3_, 125 MHz) δ: 20.01 (4-CH_3_-*i*-*Pr*), 21.19 (C-3), 24.98 (C-8), 25.42 (C-7), 27.09 (C-2), 29.55 (C-9), 32.61 (2-CH-*i*-*Pr*), 34.21 (C-15), 38.48 (C-1), 56.81 (C-4), 57.39 (C-6), 58.57 (C-12), 64.55 (C-9a), 68.78 (C-10), 79.29 (C-13), 85.14 (C-14) ppm; HR-MS, *m*/*z* (I*_rel_.*, %): 321 (2), 320.3 (4.5, [M]^+^), 305 (4), 277 (19), 221 (18), 168 (17), 153 (59), 152 (36), 151 (100), 150 (21), 136 (45), 124 (15); calcd for C_20_H_36_N_2_O: 320.2822; found *m*/*z* 320.2819 [M]^+^.

N,N-Dibutyl-4-(((1*R*,9a*R*)-octahydro-1H-quinolizin-1-yl)methoxy)but-2-yn-1-amine (**21**). Yellowish oil; yield 71%; [α]_D_^22^ = −4.2 (*c* 0.9, CHCl_3_); IR (KBr) ν: 2953, 2931, 2860, 2761 (quinolizine), 2110 (C≡C), 1103, 1086 (C-O-C) cm^–1^; ^1^H NMR (CDCl_3_, 300 MHz) δ: 0.85 (6H, t, *J* = 7.4 Hz, 2-CH_3_-20,24), 1.19 (1H, m, H-2_a_), 1.21–1.33 (5H, m, H-9_a_, 2-CH_2_-19,23), 1.31–1.50 (8H, m, H-3_a_,7_a_,8_a_,7_e_,2-CH_2_-18,22), 1.54–1.71 (4H, m, H-2_e_,3_e_,8_e_,9_e_), 1.72–1.92 (4H, m, H-1,9a,4_a_,6_a_), 2.39 (4H, t, *J* = 7.8 Hz, 2-CH_2_-17,21), 2.99–3.20 (2H, m, H-4_e_,6_e_), 3.39 (2H, br. s, H-15), 3.43-3.78 (2H, m, H-12), 4.10 (1H, dd, *J* = 10.2, 8.6 Hz, H-10) 4.16 (1H, dd, *J* = 10.2, 5.0 Hz, H-10) ppm; ^13^C NMR (CDCl_3_, 125 MHz) δ: 13.92 (C-20,24), 20.09 (C-3), 20.49 (C-19,23), 23.60 (C-7,8), 26.56 (C-2), 29.38 (C-18,22), 29.54 (C-9), 37.52 (C-1), 41.87 (C-15), 53.31 (C-17,21), 56.67 (C-4,6), 58.38 (C-12), 64.94 (C-9a), 68.47 (C-10). 80.55 (C-13), 81.02 (C-14) ppm; HR-MS, *m*/*z* (*I*_rel_, %): 349 (3), 348 (9.7), 347 (5.8, [M]^+^), 305 (38), 222 (24), 221 (100), 220 (32), 181 (36), 180 (18), 179 (47), 168 (54), 153 (32), 152 (85), 151 (24), 150 (79), 138 (29), 127 (26), 136 (33), 124 (51), 86 (39); calcd for C_22_H_39_N_2_O: 347.3057; found *m*/*z* 347.3055.

(1*R*,9a*R*)-1-((4-(Piperidin-1-yl)but-2-ynyloxy)methyl)octahydro-1*H*-quinolizine (**24**). Yellowish oil; yield 84%; [α]_D_^23^ = −11.3 (*c* 1.0, CHCl_3_); IR (KBr) ν: 2946, 2854, 2798, 2765 (quinolizine), 2160 (C≡C), 1111, 1082 (C-O-C) cm^–1^; ^1^H NMR (CDCl_3_, 300 MHz) δ: 1.11– 1.34 (2H, m, H-2_a_,9_a_), 1.34–1.48 (5H, m, H-3_a_,7_a_,8_a_,19,19), 1.48–1.64 (6H, m, H-7_e_,8_e_,18,20, 18,20), 1.64–1.83 (3H, m, H-2_e_,3_e_,9_e_), 1.83–2.08 (4H, m, H-1,9a,4_a_,6_a_), 2.36–2.55 (4H, m H-17,21,17,21), 2.70–2.92 (2H, m, 4_e_,6_e_), 3.26 (2H, br. s, H-15), 3.58 (1H, dd, *J* = 10.6, 8.4 Hz, H-10), 3.73 (1H, dd, *J* = 10.6, 5.2 Hz, H-10), 4.12 (2H, m, H-12) ppm; ^13^C NMR (CDCl_3_, 100 MHz) δ: 21.18 (C-3), 23.80 (C-19), 24.99 (C-8), 25.47 (C-7), 25.85 (C-18,20), 27.05 (C-9), 29.62 (C-2), 38.54 (C-1), 47.94 (C-15), 53.19 (C-17,21), 56.91 (C-4(6)), 57.39 (C-6(4)), 58.44 (C-12), 64.64 (C-9a), 68.89 (C-10), 81.05 (C-13), 81.48 (C-14) ppm; HR-MS, *m*/*z* (*I*_rel_, %): 305 (4), 304 (17.4, [M]^+^), 303 (5), 222 (18), 221 (97), 220 (25), 168 (53), 166 (23), 153 (37), 152 (100), 150 (28), 137 (35), 136 (37), 135 (62), 134 (26), 124 (71), 122 (22), 98 (40), 97 (21), 96 (27), 84 (42), 83 (36), 41 (33); calcd for C_19_H_32_N_2_O 304.2509; found *m*/*z*: 304.2507 [M]^+^.

(1*R*,9a*R*)-1-((4-(Azepan-1-yl)but-2-ynyloxy)methyl)octahydro-1*H*-quinolizine (**25**). Yellowish oil; yield 81%; [α]_D_^20^ = −9.4 (*c* 0.7, CHCl_3_); IR (KBr) ν: 2923, 2854, 2802, 2757 (quinolizine), 2180 (C≡C), 1103, 1061 (C-O-C) cm^–1^; ^1^H NMR (CDCl_3_, 300 MHz) δ: 1.15–1.27 (1H, m, H-2_a_), 1.33 (1H, dd, J = 12.9, 4.2 Hz, H-9_a_), 1.40–1.48 (3H, m, H-3_a_,7_a_,8_a_), 1.49–1.60 (13H, m, H-3_e_,7_e_,8_e_,20,21,18,19,18,19,20,21), 1.63–1.76 (2H, m, 2_e_,9_e_), 1.76–1.90 (2H, m, 1,9a), 1.92–2.08 (2H, m, 4_a_,6_a_), 2.55–2.59 (4H, m, H-17,22,17,22), 2.78–2.86 (2H, m, 4_e_,6_e_), 3.33 (2H, br. s, H-15), 3.56 (1H, dd, *J* = 10.3, 8.6 Hz, H-10), 3.71 (1H, dd, *J* = 10.3, 5.2 Hz, H-10), 4.10 (2H, m, H-12) ppm; ^13^C NMR (CDCl_3_, 100 MHz) δ: 21.10 (C-3), 24.73 (C-7), 25.08 (C-8), 25.85 (C-19,20), 26.61 (C-2), 27.34, 27.48 (C-18,21), 29.34 (C-9), 38.34 (C-1), 47.76 (C-15), 53.06 (C-17,22), 56.50 (C-4(6)), 57.08 (C-6(4)), 58.38 (C-12), 64.33 (C-9a), 68.72 (C-10), 79.53 (C-13), 83.01 (C-14) ppm; HR-MS, *m*/*z* (*I*_rel_, %): 333 (2), 332 (7.4, [M]^+^), 331(4), 221 (97), 168 (50), 165 (28), 164(24), 163 (100), 153 (29), 152 (99), 150 (33), 138 (26), 124 (49), 98 (22), 84 (24); calcd for C_21_H_36_N_2_O: 332.2822; found: 332.2816 [M]^+^.

### 4.3. Biochemical Method

AChE activity inhibition was evaluated by the assay described by Ellman et al. [49]. 5,5′-dithiobis(2-nitrobenzoic acid) (DTNB), AChE (AChE, E.C.3.1.1.7, Type V-S, lyophilized powder, from electric eel, 1000 units), and acetylthiocholine iodide were obtained from Sigma. The lyophilized enzyme was prepared in buffer to obtain 1130 U/mL stock solution. The enzyme stock solution was kept at −80 °C. We performed the Ellmann assay in 96-well microtiter plates in a final reaction volume of 200 mL. 1E-2 units (1 μL) of acetylcholinesterase was added to a solution of 500 μM DTNB in 0.05 M Tris-HCl pH7.5, into polystyrene wells. Then, 2 μL of solutions of tested compounds (10^−4^–10^−8^ M) in DMSO were added. The control was supplemented with 2 µL of DMSO without inhibitors. Incubation was performed at room temperature for 15 min., then 5 min., at 4 °C, on a planetary shaker. After that, 10 µL of a cooled solution containing 1 mM Tris-HCl pH7.5, 0.5 mM DTNB, and 1 μM acetylthiocholine iodide, prepared immediately before addition, were added to each well. The wells were placed in a reader (PerkinElmer 2103 Multilabel Reader, Waltham, MA, USA), and the reaction was monitored at room temperature at a wavelength of 405 nm. Each inhibitor was applied at three concentrations in half-log increments (e.g., 10, 30, and 100 μM or 1, 3, 10 μM, for active compounds) in triplicate. The measurements were carried out at a wavelength of 405 nm every 2 min. The 10th measurement was used for calculations (18 min. from the beginning of the reaction). For each well, the first measurement (background) was subtracted from the indicator of the last measurement, and the resulting numbers were used for further calculations.

Next, activity curves were built, where the point without inhibitor was taken as 100%. The points were used to build a trend line from which the inhibitor concentration where the activity was at 50% was calculated. All constructions and determination of the mean and standard deviation were made in Excel 2007 and using online services for solving equations. The curves for AChE inhibition by compounds **3, 4**, **5**, **7**, **11**, **12**, **13**, **15**, **16**, **21**, **24**, and **25** are given in the Supplementary Materials (Figure S40A–L). 

### 4.4. Docking Methodology

Molecular docking was performed against the crystal structure of human AChE in complex with donepezil (PDB ID: 4EY7), the AChE structure most frequently used in docking studies [73], using the ligand docking application of the ROSIE server [74]. The docking region was centered on the geometric center of the co-crystallized donepezil. For each compound, up to 2000 conformers were generated with the BCL algorithm [75] to adequately sample their conformational flexibility (including up to 8 internal rotational degrees of freedom and mobile rings). The number of intermediate docking poses evaluated during the protocol was set to 2000 (the maximum limit allowed in the ROSIE interface); however, only the top 10 final docking poses were requested and saved for each ligand. All other parameters were defaults of the ROSIE ligand docking protocol, which implements the RosettaLigand approach with full backbone and side-chain flexibility of residues near the docking region [76], and whose scoring framework has been independently validated in the CASF-2016 benchmark [77]. The poses with the most favorable Rosetta Interface Scores (IScores) were downloaded and imported into Molegro Virtual Docker 6.0 software, where binding modes and hydrogen-bond, π–π stacking and van der Waals contacts were analyzed with the built-in “Pose Organizer” tool. The IScores were compared with the experimental pIC_50_ values by Pearson correlation analysis.

To validate the docking protocol, a self-docking of the co-crystallized ligand (donepezil) back into the 4EY7 structure was performed, using the ROSIE server. The root-mean-square deviation (RMSD) between the generated docked pose and the original crystal conformation was 0.685 Å for heavy atoms, which is well within the acceptable limit (<2.0 Å), confirming the reliability of the computational setup.

## 5. Conclusions

In summary, we have proposed copper-catalyzed azide–alkyne cycloaddition (CuAAC) and A^3^-coupling methods to conjugate the lupinine scaffold with various alkaloids (anabasine, cytisine, methylxanthines, and colchicine) and nitrogen-containing substituents, such as 10-(4-aminobut-2-ynyloxy), to generate novel candidate molecules as acetylcholinesterase (AChE) inhibitors. Six of the new compounds exhibited AChE inhibitory activity comparable to that of galantamine, with 1,3,7-trimethyl-8-(1-(((1*S*,9a*R*)-octahydro-1*H*-quinolizin-1-yl)methyl)-1*H*-1,2,3-triazol-4-yl)-1*H*-purine-2,6(3*H*,7*H*)-dione (**12**) demonstrating inhibitory activity against AChE in the nanomolar range. The inhibitory activity of compound **12** was comparable to that of donepezil. The binding mode of the most active molecules with AChE was analyzed, using molecular docking methods. Most of the active compounds interacted with amino acid residues Tyr341, Phe338, Trp86, and Phe297, which are key components of the AChE binding pocket. This indicates that all ligands are well-accommodated within the catalytic center, which may explain the observed in vitro activity.

Overall, some of the described lupinine derivatives have demonstrated promising properties for further investigation. Furthermore, due to the simplicity and efficiency of the proposed CuAAC and A^3^-coupling methods, it is highly likely that this new modification of the lupinine core will find application in the development of compounds that can serve as scaffolds for creating other libraries of biologically active substances.

## Figures and Tables

**Table 1:** AChE inhibitory activity of 10-*O*-(prop-2-ynyl)lupinine **3**, N-(prop-2-ynyl)anabazine **4**, triazole-linked hybrids **5**, **7**, **11**–**13**, **15**, **16**, N-substituted (4-(amino)but-2-ynyloxy)methyl)octa-hydro-1*H*-quinolizines **21**, **24**, **25**, the parent compounds (−)-lupinine 1, and the reference compounds Galantamine and Donepezil.

Compound	IC_50_ ± SEM, μM ^[a]^	Compound	IC_50_ ± SEM, μM ^[a]^
**3**	15.0 ± 0.5	**16**	1.4 ± 0.05
**5**	20.1 ± 0.1	**21**	13.0 ± 0.4
**7**	47.0 ± 1.1	**24**	4.0 ± 0.2
**11**	0.47 ± 0.01	**25**	3.4 ± 0.1
**12**	0.035 ± 0.0001	N-(Prop-2-ynyl)anabazine **4**	260.0 ± 1.1
**13**	5.8 ± 0.1	(−)-Lupinine **1**	65.4 ± 2.1
**15**	1.3 ± 0.13	Galantamine	3.9 ± 0.2
		Donepezil	0.04 ± 0.003

^[a]^ Data represent the mean (±standard error of mean, SEM) of three independent experiments.

**Table 2:** AChE inhibitory activities, Rosetta Interface Scores (IScores) and key ligand–receptor interactions of the docked lupinine hybrids (PDB ID: 4EY7).

Compound	Hybrid Type (Distal Fragment, Linker)	IC_50_ ± SEM, μM	pIC_50_	IScore	Key Interactions *
**11**	lupinine–theophylline (N7–CH_2_–triazole)	0.47 ± 0.01	6.33	−19.643	Phe295 (HB, xanthine nitrogen), Tyr124 (HB, triazole); Tyr341, Trp86, Trp286, Phe338, Gly121, Phe297, Tyr337
**12**	lupinine-caffeine (C8–triazole)	0.035 ± 0.0001	7.46	−20.945	Tyr337 (HB, triazole), Arg296 (HB, xanthine oxygen), Tyr124 (HB, triazole), Phe295 (HB, weak, xanthine oxygen); Tyr341, Trp286, Trp86, Phe338, His447, Gly121, Val294, Phe297
**13**	lupinine–theobromine (N1–CH_2_–triazole)	5.8 ± 0.1	5.24	−18.458	Tyr124 (HB, xanthine oxygen), Phe295 (HB, xanthine oxygen), Tyr341 (π–π, triazole), Trp286 (π–π, xanthine); Tyr337, Phe338, Phe297, Trp86
**15**	lupinine–colchicine (triazole–CH_2_–NH)	1.3 ± 0.13	5.89	−18.755	Asp74 (HB, acetamide NH); Trp286, Phe338, Tyr341, Tyr337, Tyr124, Trp86, His447, Phe297, Gly121, Tyr72
**16**	bis-lupinine (triazole–CH_2_–O–CH_2_)	1.4 ± 0.05	5.85	−17.865	Tyr124 (HB, triazole); Trp286, Tyr341, His447, Tyr337, Phe338, Trp86
**24**	lupinine–piperidine (O–CH_2_–C≡C–CH_2_)	4.0 ± 0.2	5.38	−16.696	Tyr124 (HB, ether oxygen); Trp86, Trp286, Tyr341, Phe297, Gly121
**25**	lupinine–azocane (O–CH_2_–C≡C–CH_2_)	3.4 ± 0.1	5.47	−15.817	Tyr341, Phe338, Trp286, Trp86, Tyr337, Tyr124, His447, Gly121, Phe297

* HB: hydrogen bond; π–π: stacking interaction. Unlabeled residues participate in van der Waals interactions, listed in descending order of strength. pIC_50_ = −log IC_50_ (IC_50_ in mol/L); SEM: standard error of the mean; IScore: a unitless Rosetta Interface Score (more negative values indicate more favorable predicted binding).

## Data Availability

Data are contained within the article.

## Supplementary Materials

The following supporting information can be downloaded at: https://www.mdpi.com/article/10.3390/molecules31213961/s1, Copies of 1H, 13C NMR spectra and HR-MS spectra of all new compounds (Figures S1–S39); The curves for AChE inhibition by compounds **3, 4**, **5**, **7**, **11**, **12**, **13**, **15**, **16**, **21**, **24**, and **25** (Figure S40A–L).

## Abbreviations

The following abbreviations are used in this manuscript:

AD	Alzheimer’s disease
CuAAC	Copper-catalyzed azide–alkyne cycloaddition
AChE	Acetylcholinesterase
MAO	Monoamine oxidase
