# Supplementary material for: Design and Synthesis of New Derivatives of Alkaloid Lupinine: Structural Characterization, Acetylcholinesterase Inhibition Evaluation, and Molecular Modeling Study
Source: Molecules. 2026 Nov 3;31(21):3961. doi: 10.3390/molecules31213961 (PMC13765206; doi:10.3390/molecules31213961)
Supplement: Supplementary file 1 [file molecules-31-03961-s001.zip › molecules-4633502-supplementary.pdf]

SUPPLEMENTARY MATERIAL

Design and Synthesis of New Derivatives of Alkaloid Lupinine: Structural Characterization, Acetylcholinesterase Inhibition Evaluation, and Molecular Modeling Study

Zhangeldy S. Nurmaganbetov [1,*], Igor D. Ivanov [2], Oralgazy A. Nurkenov [1], Victor A. Savelyev [3], Andrey I. Khlebnikov [4], Dastan M. Turdybekov [5], Aizhan M. Kozhanova [1], Serik D. Fazylov [1], Zainulla M. Muldakhmetov [1] and Elvira E. Shults [3,*]

[1] Laboratory of Synthesis of Biologically Active Substances, Institute of Organic Synthesis and Coal Chemistry, Karaganda 100008, Kazakhstan; nurkenov_oral@mail.ru (O.A.N.); nurs-djoni@mail.ru (A.M.K.); iosu8990@mail.ru (S.D.F.); iosu.rk@mail.ru (Z.M.M.)
[2] Institute of Molecular Biology and Biophysics, Timakova Str 14, Novosibirsk 630090, Russia; diadoryh@mail.ru
[3] N.N. Vorozhtsov Novosibirsk Institute of Organic Chemistry, Siberian Branch of the Russian Academy of Sciences, Novosibirsk 630090, Russia; vicsav@nioch.nsc.ru
[4] Kizhner Research Center, Tomsk Polytechnic University, Tomsk 634050, Russia; aikhl@chem.org.ru
[5] Department of Physics, Abylkas Saginov Karaganda Technical University, Karaganda 100027, Kazakhstan; turdas@mail.ru
* Correspondence: nzhangeldy@yandex.ru (Z.S.N.); schultz@nioch.nsc.ru (E.E.S.)

Table of contents

		Page
1	Figure S1. ^{1}H NMR spectrum of (1*R*,9a*R*)-1-((prop-2-ynyloxy)methyl)octahydro-1*H*-quinolizine (**3**) ($CDCl_3$, 500 MHz).	3
2	Figure S2. ^{13}C NMR spectrum of (1*R*,9a*R*)-1-((prop-2-ynyloxy)methyl)octahydro-1*H*-quinolizine (**3**) ($CDCl_3$, 125 MHz).	4
3	Figure S3. HR-MS spectrum of (1*R*,9a*R*)-1-((prop-2-ynyloxy)methyl)octahydro-1*H*-quinolizine (**3**)	5
4	Figure S4. ^{1}H NMR spectrum of (*S*)-N-(prop-2-ynyl)anabasine (**4**) ($CDCl_3$, 400 MHz).	6
5	Figure S5. ^{13}C NMR spectrum of (*S*)-N-(prop-2-ynyl)anabasine (**4**) ($CDCl_3$, 101 MHz).	7
6	Figure S6. HR_MS spectrum of (*S*)-N-(prop-2-ynyl)anabasine (**4**)	8
7	Figure S7. ^{1}H NMR spectrum of (1*S*,9a*R*)-1-((4-(((*S*)-2-(pyridin-3-yl)piperidin-1-yl)methyl)-1*H*-1,2,3-triazol-1-yl)methyl)octahydro-1*H*-quinolizine (**5**) ($CDCl_3$, 400 MHz).	9
8	Figure S8. ^{13}C NMR spectrum of (1*S*,9a*R*)-1-((4-(((*S*)-2-(pyridin-3-yl)piperidin-1-yl)methyl)-1H-1,2,3-triazol-1-yl)methyl)octahydro-1*H*-quinolizine (**5**) ($CDCl_3$, 125 MHz).	10
9	Figure S9. HR-MS spectrum of (1*S*,9a*R*)-1-((4-(((*S*)-2-(pyridin-3-yl)piperidin-1-yl)methyl)-1H-1,2,3-triazol-1-yl)methyl)octahydro-1*H*-quinolizine (**5**)	11
10	Figure S10. ^{1}H NMR spectrum of (1*R*,5*S*)-3-(1-(((1*S*,9a*R*)-octahydro-1*H*-quinolizin-1-yl)methyl)-1*H*-1,2,3-triazol-4-yl)methyl-1,2,3,4,5,6-hexahydro-8*H*-1,5-methanopyrido[1,2-*a*][1,5]diazocine-8 one (**7**) ($CDCl_3$, 400 MHz).	12
11	Figure S11. ^{13}C NMR spectrum of (1*R*,5*S*)-3-(1-(((1*S*,9a*R*)-octahydro-1*H*-quinolizin-1-yl)methyl)-1*H*-1,2,3-triazol-4-yl)methyl-1,2,3,4,5,6-hexahydro-8*H*-1,5-methanopyrido[1,2-*a*][1,5]diazocine-8 one (**7**) ($CDCl_3$, 125 MHz).	13
12	Figure S12. HR-MS spectrum of (1*R*,5*S*)-3-(1-(((1*S*,9a*R*)-octahydro-1*H*-quinolizin-1-yl)methyl)-1*H*-1,2,3-triazol-4-yl)methyl-1,2,3,4,5,6-hexahydro-8*H*-1,5-methanopyrido[1,2-*a*][1,5]diazocine-8 one (**7**).	14
13	Figure S13. ^{1}H NMR spectrum of 1,3-dimethyl-7-((1-(((1*S*,9a*R*)-octahydro-1*H*-quinolizin-1-yl)methyl)-1*H*-1,2,3-triazol-4-yl) methyl)-1*H*-purine-2,6-dione (**11**) ($CDCl_3$, 500 MHz).	15
14	Figure S14. ^{13}C NMR spectrum of 1,3-dimethyl-7-((1-(((1*S*,9a*R*)-octahydro-1*H*-quinolizin-1-yl)methyl)-1*H*-1,2,3-triazol-4-yl) methyl)-1*H*-purine-2,6-dione (**11**) ($CDCl_3$, 125	16

MHz).
15 Figure S15. HR-MS spectrum of 1,3-dimethyl-7-((1-(((1*S*,9a*R*)-octahydro-1*H*-quinolizin-1-yl)methyl)-1*H*-1,2,3-triazol-4-yl) methyl)-1*H*-purine-2,6(3*H*,7*H*)-dione (**11**) 17
16 Figure S16. ^{1}H NMR spectrum of of 1,3,7-trimethyl-8-(1-(((1*S*,9a*R*)-octahydro-1*H*-quinolizin-1-yl)methyl)-1*H*-1,2,3-triazol-4-yl)-1*H*-purine-2,6-dione (**12**) (CDCl$_3$, 400 MHz). 18
17 Figure S17. ^{13}C NMR spectrum of 1,3,7-trimethyl-8-(1-(((1*S*,9a*R*)-octahydro-1*H*-quinolizin-1-yl)methyl)-1*H*-1,2,3-triazol-4-yl)-1*H*-purine-2,6-dione (**12**) (CDCl$_3$, 125 MHz). 19
18 Figure S18. HR-MS spectrum of 1,3,7-trimethyl-8-(1-(((1*S*,9a*R*)-octahydro-1*H*-quinolizin-1-yl)methyl)-1*H*-1,2,3-triazol-4-yl)-1*H*-purine-2,6-dione (**12**) 20
19 Figure S19. ^{1}H NMR spectrum 3,7-dimethyl-1-((1-(((1*S*,9a*R*)-octahydro-1*H*-quinolizin-1-yl)methyl)-1*H*-1,2,3-triazol-4-yl)methyl)-1*H*-purine-2,6(3*H*,7*H*)-dione (**13**) (CDCl$_3$, 400 MHz). 21
20 Figure S20. ^{13}C NMR spectrum of 3,7-dimethyl-1-((1-(((1*S*,9a*R*)-octahydro-1*H*-quinolizin-1-yl)methyl)-1*H*-1,2,3-triazol-4-yl)methyl)-1*H*-purine-2,6(3*H*,7*H*)-dione (**13**) (CDCl$_3$, 125 MHz). 22
21 Figure S21. HR-MS spectrum of 3,7-dimethyl-1-((1-(((1*S*,9a*R*)-octahydro-1*H*-quinolizin-1-yl)methyl)-1*H*-1,2,3-triazol-4-yl)methyl)-1*H*-purine-2,6-dione (**13**). 23
22 Figure S22. ^{1}H NMR spectrum of 1*R*,5*S*)-10-(1-(((1*S*,9a*R*)-octahydro-1*H*-quinolizin-1-yl)methyl)-1*H*-1,2,3-triazol-4-yl)methylamino-N-[(7*S*)-acetylamino-1,2,3,-trimethoxy-6,7-dihydro-5*H*-benzo[*a*]heptalen-9-one (**15**) (CDCl$_3$, 400 MHz). 24
23 Figure S23. ^{13}C NMR spectrum of 1*R*,5*S*)-10-(1-(((1*S*,9a*R*)-octahydro-1*H*-quinolizin-1-yl)methyl)-1*H*-1,2,3-triazol-4-yl)methylamino-N-[(7*S*)-acetylamino-1,2,3,-trimethoxy-6,7-dihydro-5*H*-benzo[*a*]heptalen-9-one (**15**) (CDCl$_3$, 125 MHz). 25
24 Figure S24. HR-MS spectrum of of 1*R*,5*S*)-10-(1-(((1*S*,9a*R*)-octahydro-1*H*-quinolizin-1-yl)methyl)-1*H*-1,2,3-triazol-4-yl)methylamino-N-[(7*S*)-acetylamino-1,2,3,-trimethoxy-6,7-dihydro-5*H*-benzo[*a*]heptalen-9-one (**15**). 26
25 Figure S25. ^{1}H NMR spectrum of (1*S*,9a*R*)-1-((4-((((1*S*,9a*S*)-octahydro-1*H*-quinolizin-1-yl)methoxy)methyl)-1*H*-1,2,3-triazol-1-yl)methyl)octahydro-1*H*-quinolizine (**16**) (CDCl$_3$, 300 MHz). 27
26 Figure S26. ^{13}C NMR spectrum of (1*S*,9a*R*)-1-((4-((((1*S*,9a*S*)-octahydro-1*H*-quinolizin-1-yl)methoxy)methyl)-1H-1,2,3-triazol-1-yl)methyl)octahydro-1*H*-quinolizine (**16**) (CDCl$_3$, 125 MHz). 28
27 Figure S27. HR-MS spectrum of (1*S*,9a*R*)-1-((4-((((1*S*,9a*S*)-octahydro-1*H*-quinolizin-1-yl)methoxy)methyl)-1*H*-1,2,3-triazol-1-yl)methyl)octahydro-1*H*-quinolizine (**16**) 29
28 Figure S28. ^{1}H NMR spectrum of N,N-diisopropyl-4-(((1*R*,9a*R*)-octahydro-1*H*-quinolizin-1-yl)methoxy)but-2-yn-1-amine (**20**) (CDCl$_3$, 300 MHz). 30
29 Figure S29. ^{13}C NMR spectrum of N,N-diisopropyl-4-(((1*R*,9a*R*)-octahydro-1*H*-quinolizin-1-yl)methoxy)but-2-yn-1-amine (**20**) (CDCl$_3$, 125 MHz). 31
30 Figure S30. HR-MS spectrum of N,N-diisopropyl-4-(((1*R*,9a*R*)-octahydro-1*H*-quinolizin-1-yl)methoxy)but-2-yn-1-amine (**20**) 32
31 Figure S31. ^{1}H NMR spectrum of N,N-dibutyl-4-(((1*R*,9a*R*)-octahydro-1*H*-quinolizin-1-yl)methoxy)but-2-yn-1-amine (**21**) (CDCl$_3$, 500 MHz). 33
32 Figure S32. ^{13}C NMR spectrum of N,N-dibutyl-4-(((1*R*,9a*R*)-octahydro-1*H*-quinolizin-1-yl)methoxy)but-2-yn-1-amine (**21**) (CDCl$_3$, 125 MHz). 34
33 Figure S33. HR-MS spectrum of N,N-dibutyl-4-(((1*R*,9a*R*)-octahydro-1*H*-quinolizin-1-yl)methoxy)but-2-yn-1-amine (**21**). 35
34 Figure S34. ^{1}H NMR spectrum of (1R,9aR)-1-((4-(piperidin-1-yl)but-2-ynyloxy)methyl)octahydro-1*H*-quinolizine (**24**) (CDCl$_3$, 300 MHz). 36
35 Figure S35. ^{13}C NMR spectrum of (1R,9aR)-1-((4-(piperidin-1-yl)but-2-ynyloxy)methyl)octahydro-1*H*-quinolizine (**24**) (CDCl$_3$, 100 MHz). 37
36 Figure S36. HR-MS spectrum of (1*R*,9a*R*)-1-((4-(piperidin-1-yl)but-2-ynyloxy)methyl)octahydro-1H-quinolizine (**24**). 38
37 Figure S37. ^{1}H NMR spectrum of (1*R*,9a*R*)-1-((4-(azepan-1-yl)but-2-ynyloxy)methyl)octahydro-1*H*-quinolizine (**25**) (CDCl$_3$, 300 MHz). 39
38 Figure S38. ^{13}C NMR spectrum of (1*R*,9a*R*)-1-((4-(azepan-1-yl)but-2-ynyloxy)methyl)octahydro-1*H*-quinolizine (**25**) (CDCl$_3$, 100 MHz). 40
39 Figure S39. HR-MS spectrum of (1*R*,9a*R*)-1-((4-(azepan-1-yl)but-2-ynyloxy)methyl)octahydro-1*H*-quinolizine (**25**). 41
40 Figure S40A–L: The curves for AChE inhibition by compounds **3**, **4**, **5**, **7**, **11**, **12**, **13**, **15**, **16**, **21**, **24**, and **25**. 42-43

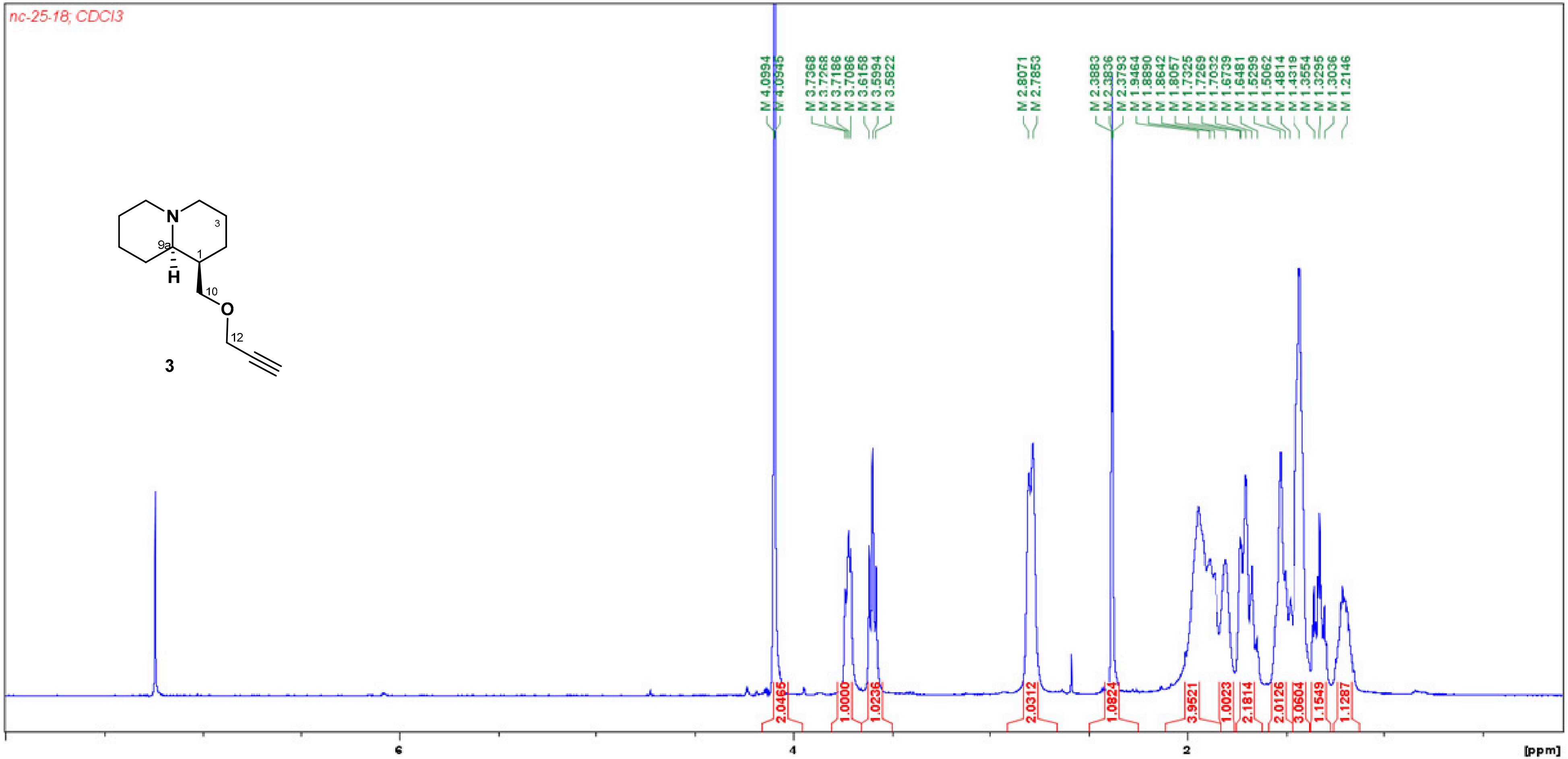

Figure S1. ^{1}H NMR spectrum of (1*R*,9a*R*)-1-((prop-2-ynyloxy)methyl)octahydro-1*H*-quinolizine (**3**) ($CDCl_3$, 500 MHz).

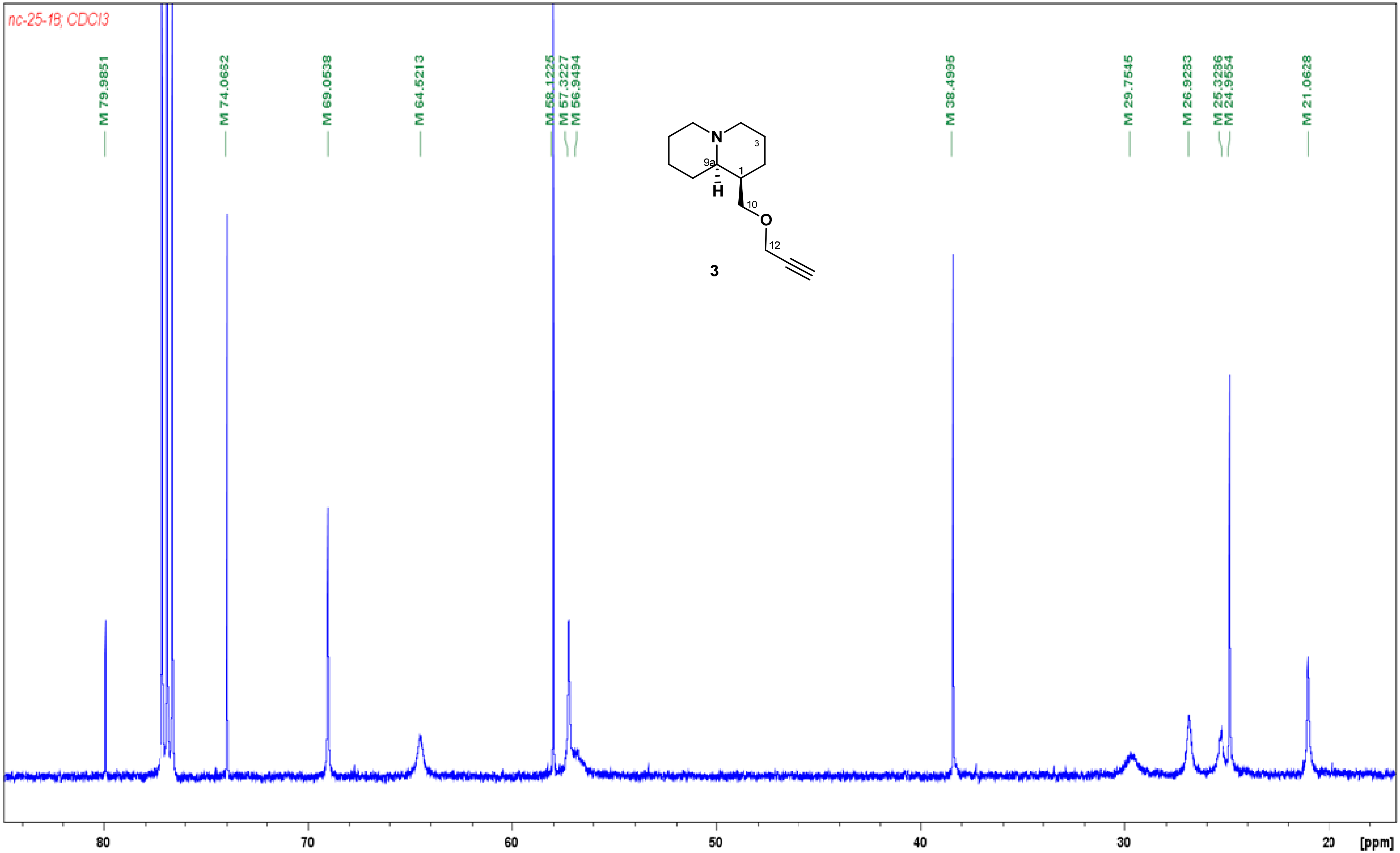

Figure S2. ^{13}C NMR spectrum of (1*R*,9a*R*)-1-((prop-2-ynyloxy)methyl)octahydro-1*H*-quinolizine (**3**) ($CDCl_3$, 125 MHz).

Figure S3. HR-MS spectrum of (1*R*,9a*R*)-1-((prop-2-ynyloxy)methyl)octahydro-1*H*-quinolizine (**3**)

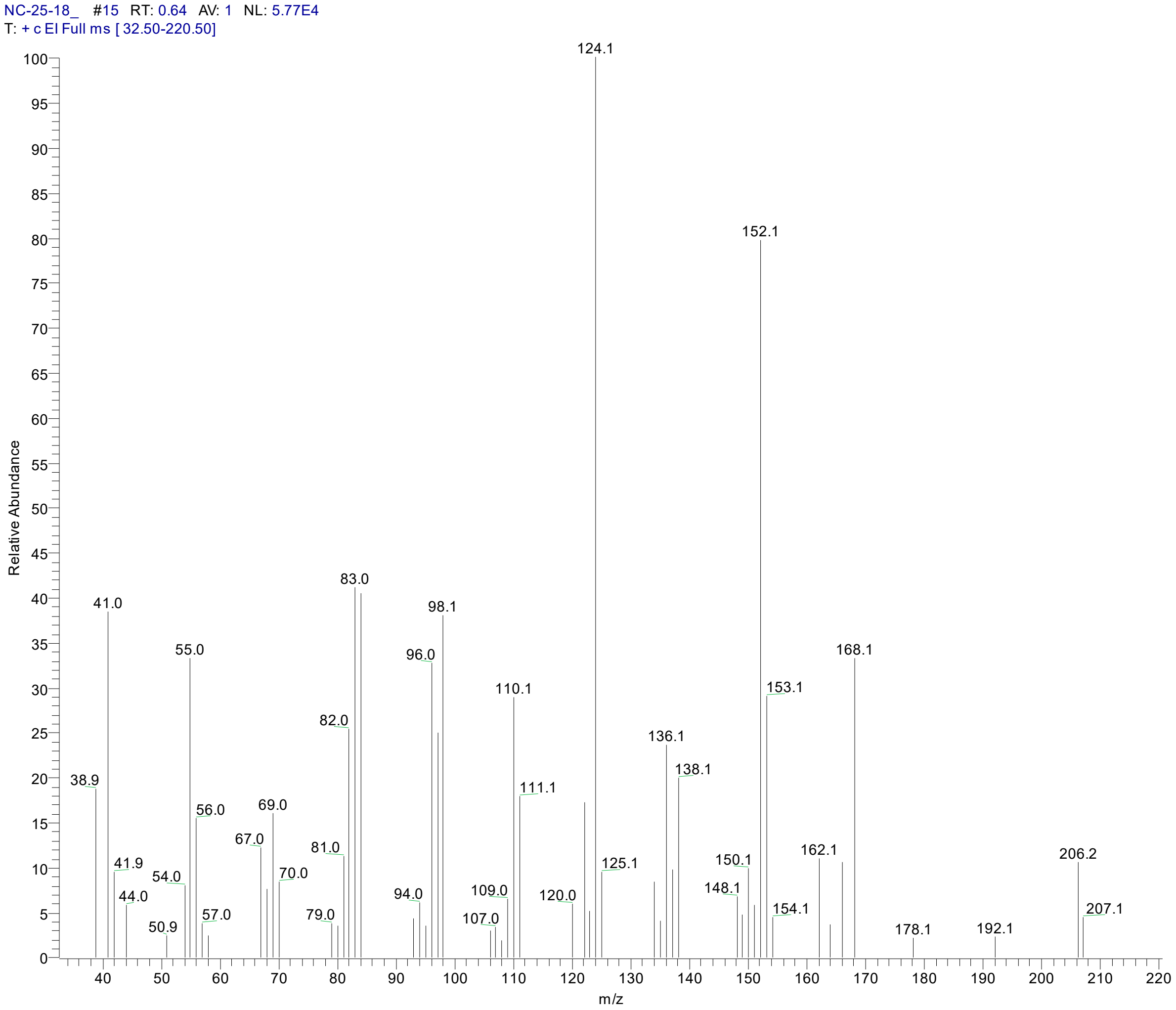

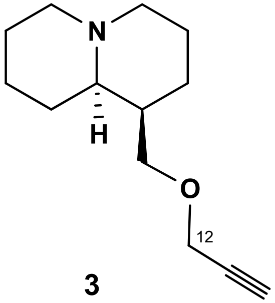

3

Tsource=90˚C

Tprobe=180˚C

Calculated: m/z= 206.1539

(C13 H20 O1 N1)$^{+\cdot}$

Found: m/z= 206.1538

1	C13 H20 O1 N1
RDB	(ppm)
4.5	-0.684

Isotope	min	max
12 C	10	16
1 H	15	30
16 O	0	3
14 N	0	3

m/z	Intensity	Relative
38.9	10768.0	18.65
41.0	22124.0	38.32
41.9	5437.0	9.42
44.0	3358.0	5.82
54.0	4558.0	7.89
54.9	3465.0	6.00
55.0	19191.0	33.24

m/z	Intensity	Relative
56.0	8876.0	15.37
67.0	7037.0	12.19
68.0	4318.0	7.48
69.0	5784.0	10.02
69.0	9249.0	16.02
70.0	4824.0	8.36
81.0	5944.0	10.30
81.0	6450.0	11.17
82.0	14607.0	25.30
83.0	23750.0	41.14
84.1	23323.0	40.40
94.0	3491.0	6.05
96.0	18872.0	32.69
97.0	14367.0	24.88
98.0	9356.0	16.21
98.1	21910.0	37.95
109.0	3731.0	6.46
110.1	16633.0	28.81
111.1	10315.0	17.87
120.0	3385.0	5.86
122.1	9915.0	17.17
123.1	2958.0	5.12
124.1	57735.0	100.00
125.1	5464.0	9.46
134.1	4824.0	8.36
136.1	13647.0	23.64
137.1	5597.0	9.69
138.1	11488.0	19.90
148.1	3918.0	6.79
149.1	2692.0	4.66
150.1	5650.0	9.79
151.1	3305.0	5.72
152.1	45980.0	79.64
153.1	16766.0	29.04
162.1	6317.0	10.94
166.1	6077.0	10.53
168.1	19191.0	33.24
206.2	6077.0	10.53
207.1	2585.0	4.48

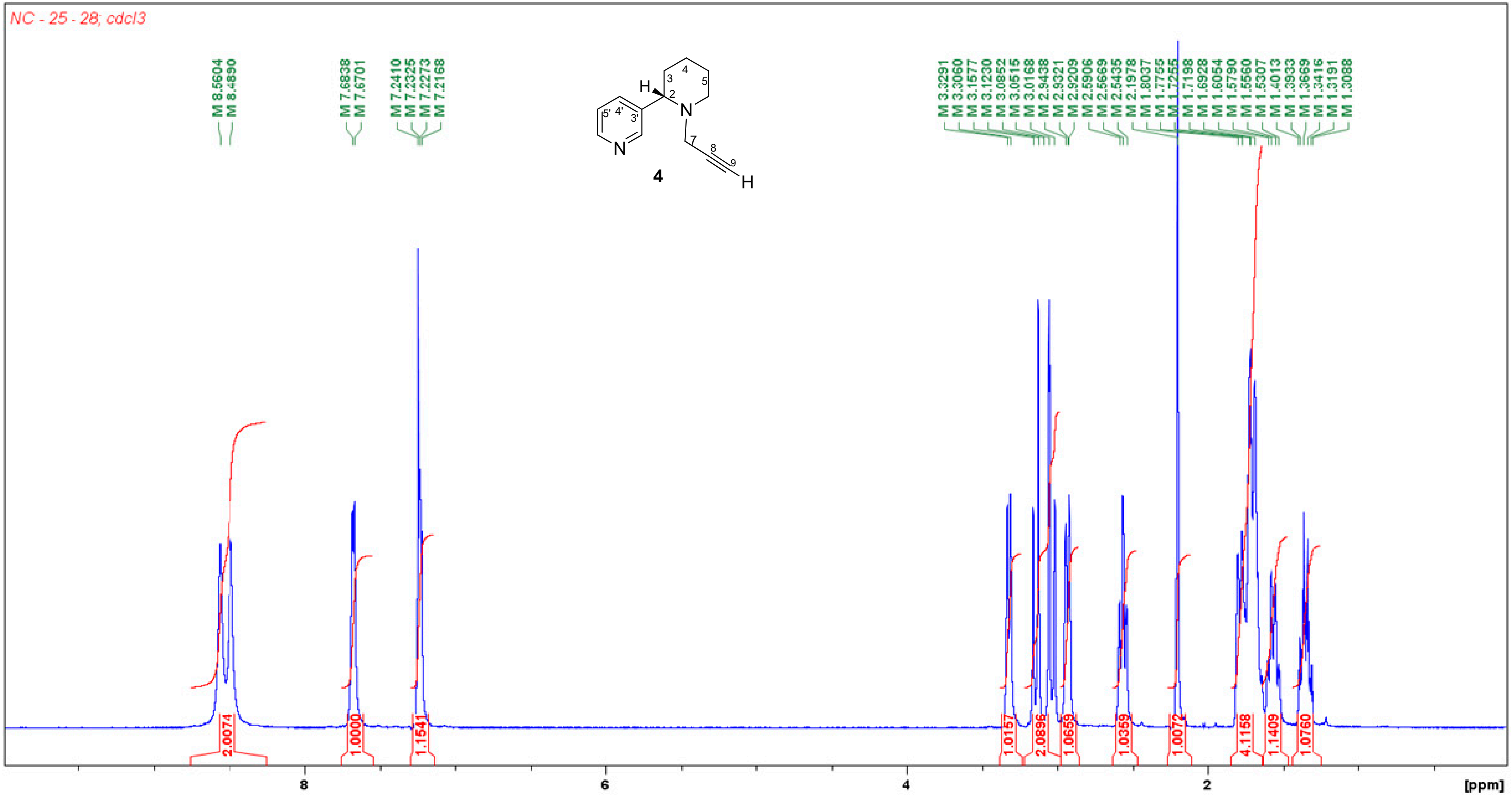

Figure S4. ^{1}H NMR spectrum of (*S*)-N-(prop-2-ynyl)anabasine (**4**) ($CDCl_3$, 400 MHz).

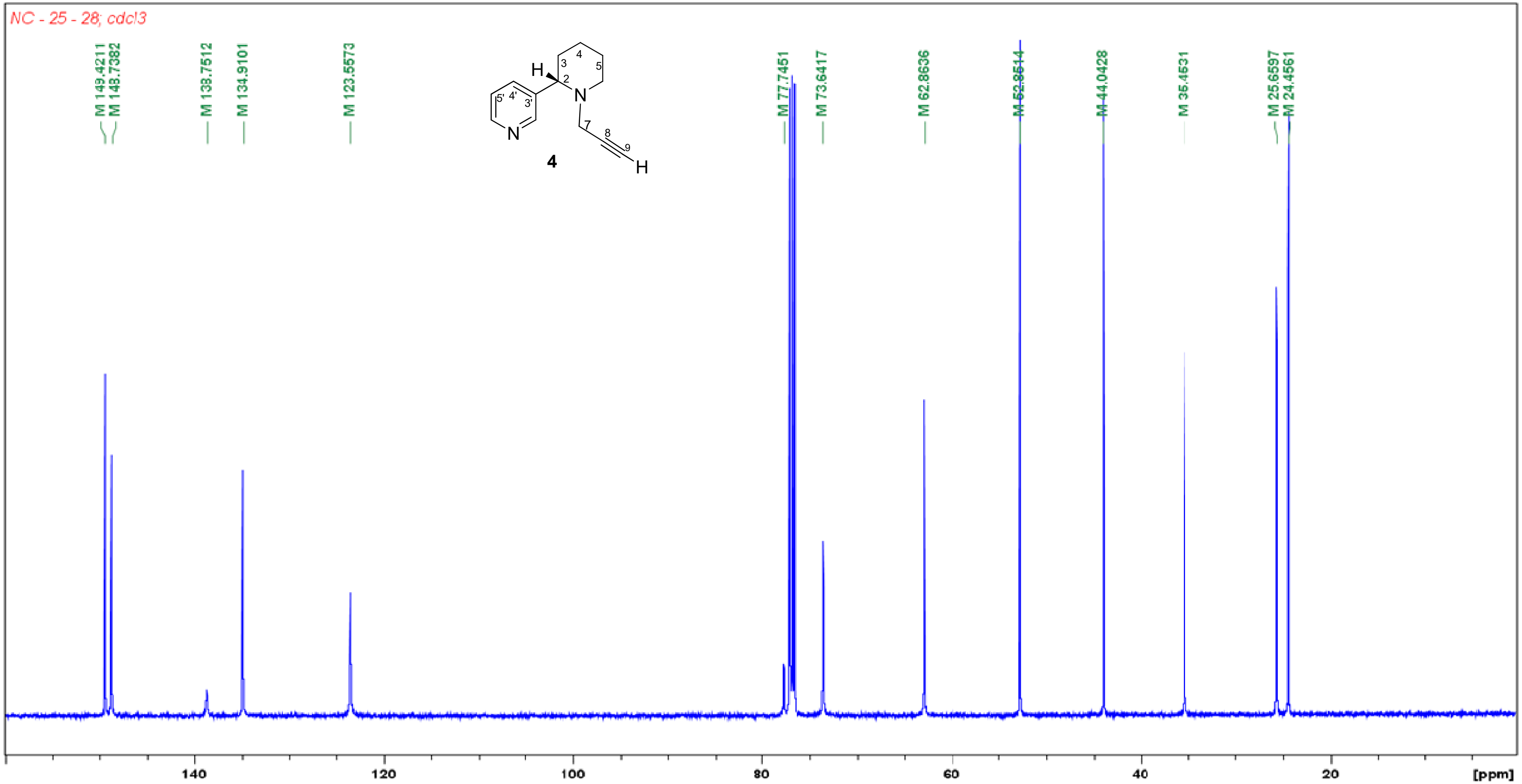

Figure S5. ^{13}C NMR spectrum of (*S*)-N-(prop-2-ynyl)anabasine (**4**) ($CDCl_3$, 101 MHz).

Figure S6. HR-MS spectrum of (*S*)-N-(prop-2-ynyl)anabasine (**4**)

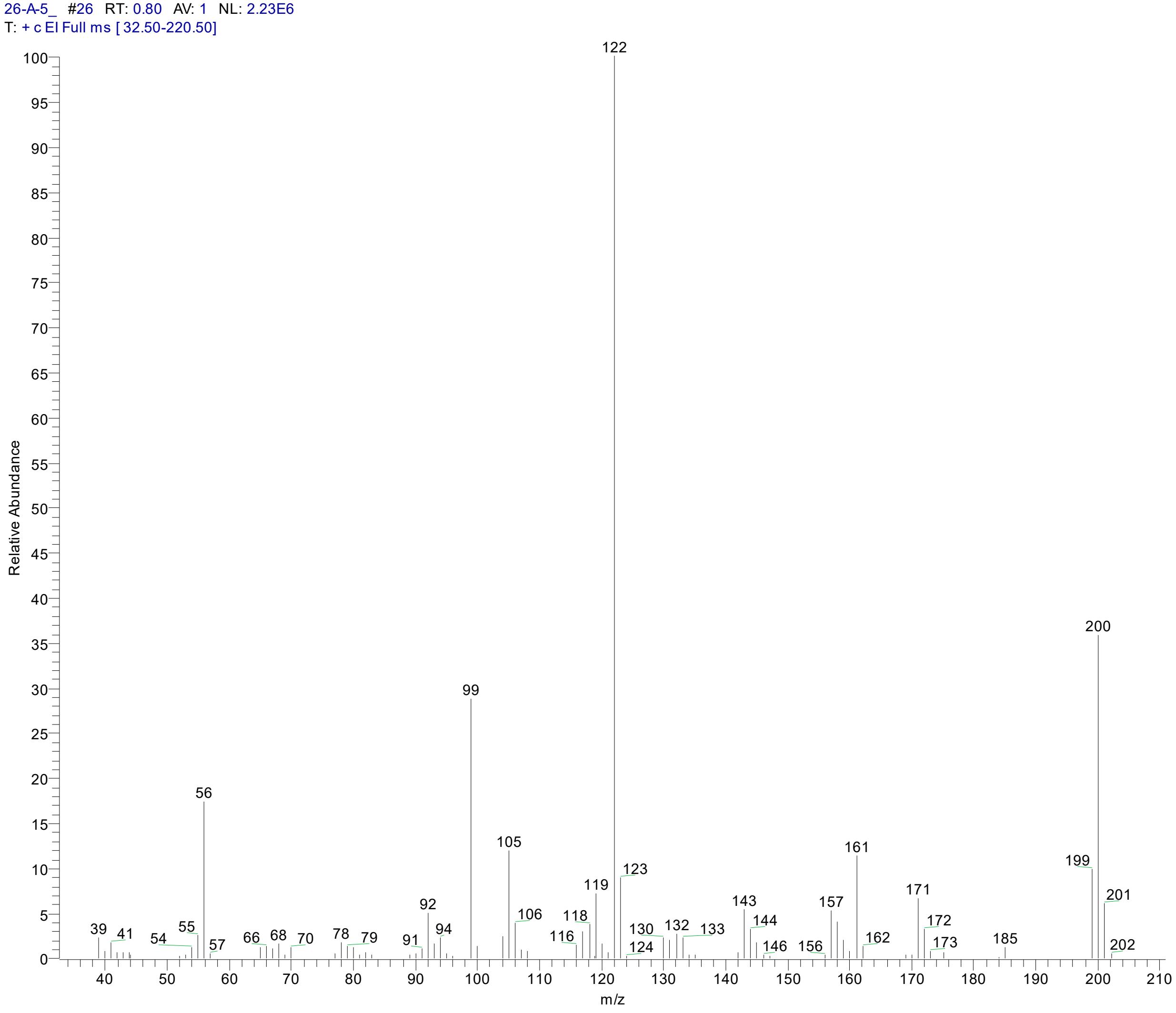

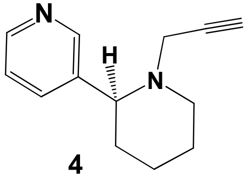

4

Calculated:
m/z= 200.1308
(C13 H16 N2)$^{+\cdot}$
Found: m/z= 200.1307

1 C13 H16 N2

RDB	(ppm)
7.0	-0.501

Isotope	min	max
12 C	10	15
1 H	10	25
14 N	0	4

m/z	Intensity	Relative
39	50613.0	2.27
41	38530.0	1.73
55	54813.0	2.46
55	50717.0	2.27
56	385667.0	17.27
65	25877.0	1.16
66	30077.0	1.35
68	36248.0	1.62
70	26240.0	1.18
78	37545.0	1.68
79	29766.0	1.33

m/z	Intensity	Relative
80	25514.0	1.14
92	110301.0	4.94
93	34018.0	1.52
94	50405.0	2.26
99	642468.0	28.78
100	29662.0	1.33
104	53361.0	2.39
105	264112.0	11.83
106	84995.0	3.81
116	30440.0	1.36
117	65600.0	2.94
118	84528.0	3.79
119	159929.0	7.16
120	34122.0	1.53
122	2232588.0	100.00
123	200016.0	8.96
130	50561.0	2.26
131	45271.0	2.03
132	58703.0	2.63
133	49472.0	2.22
143	119843.0	5.37
144	72238.0	3.24
145	36404.0	1.63
157	116472.0	5.22
158	88573.0	3.97
159	42990.0	1.93
161	253585.0	11.36
162	28158.0	1.26
171	148573.0	6.65
172	71045.0	3.18
185	26914.0	1.21
199	221381.0	9.92
200	800064.0	35.84
201	134726.0	6.03

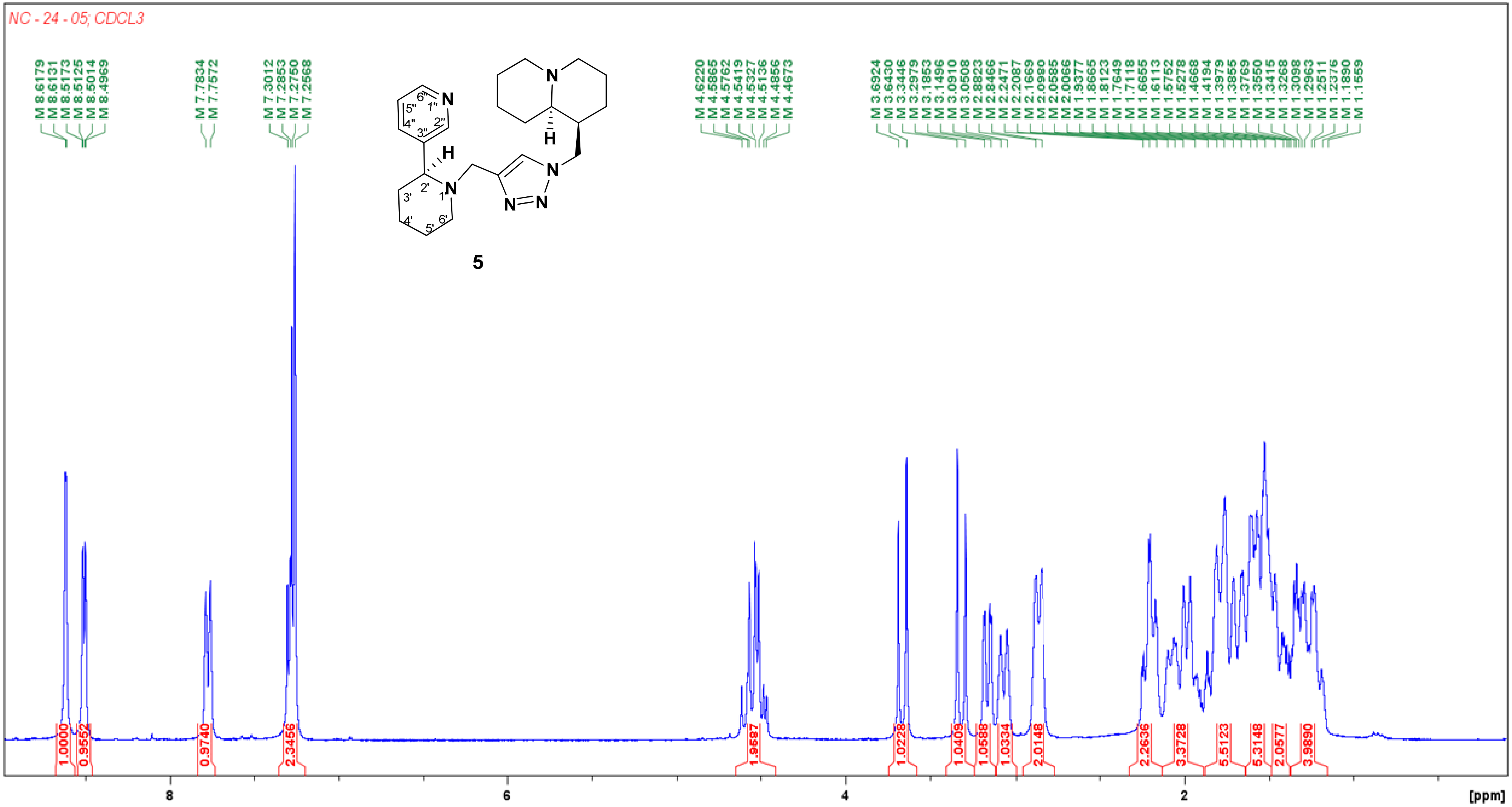

Figure S7. ^{1}H NMR spectrum of (1*S*,9a*R*)-1-((4-(((*S*)-2-(pyridin-3-yl)piperidin-1-yl)methyl)-1H-1,2,3-triazol-1-yl)methyl)octahydro-1*H*-quinolizine (**5**) ($CDCl_3$, 400 MHz).

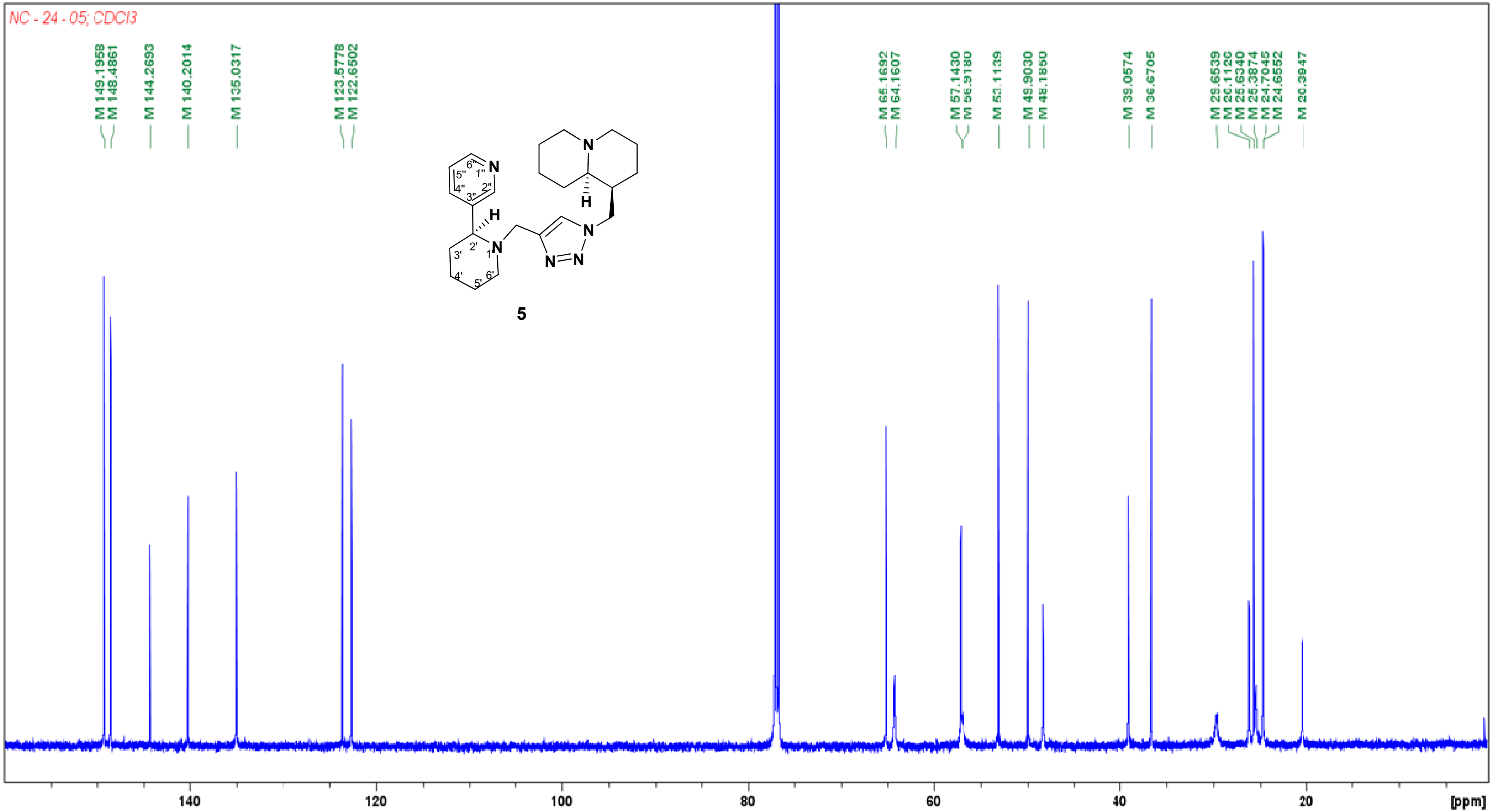

Figure S8. ^{13}C NMR spectrum of of (1*S*,9a*R*)-1-((4-(((*S*)-2-(pyridin-3-yl)piperidin-1-yl)methyl)-1H-1,2,3-triazol-1-yl)methyl)octahydro-1*H*-quinolizine (**5**) ($CDCl_3$), 125 MHz).

Figure S9. HR-MS spectrum of (1*S*,9a*R*)-1-((4-(((*S*)-2-(pyridin-3-yl)piperidin-1-yl)methyl)-1H-1,2,3-triazol-1-yl)methyl)octahydro-1*H*-quinolizine (**5**)

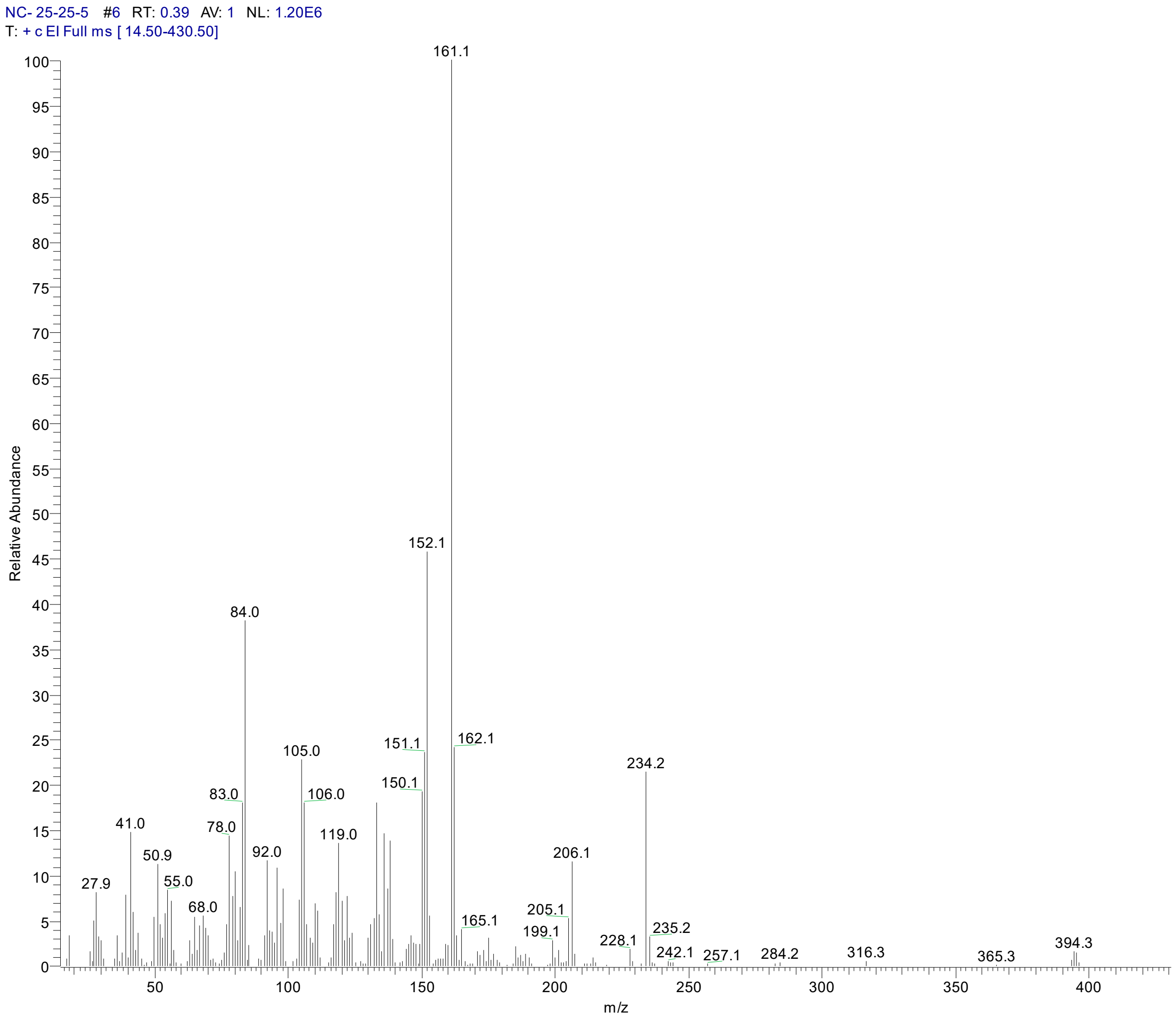

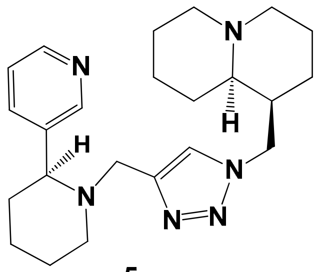

5

Tsource=100˚C
Tprobe=250˚C

Calculated: **m/z= 394.2840**
(C23 H34 N6) $^{+\cdot}$
Found: **m/z= 394.2836**

1	C23 H34 N6
RDB	(ppm)
10.0	-0.880

Isotope	min	max
12 C	20	26
1 H	25	40
14 N	0	8

m/z	Intensity	Relative
41.0	176680.0	14.74
50.9	134194.0	11.20
78.0	171025.0	14.27
80.0	124430.0	10.38
83.0	215921.0	18.02
84.0	457521.0	38.17
92.0	139786.0	11.66
96.0	129837.0	10.83

m/z	Intensity	Relative
105.0	272992.0	22.78
106.0	215056.0	17.94
119.0	161972.0	13.51
133.0	215736.0	18.00
136.1	175011.0	14.60
137.1	102368.0	8.54
138.1	164475.0	13.72
150.1	230228.0	19.21
151.1	283034.0	23.61
152.1	547900.0	45.71
161.1	1198540.0	100.00
162.1	289708.0	24.17
171.0	19188.0	1.60
172.0	13842.0	1.15
173.1	20764.0	1.73
175.1	36368.0	3.03
177.1	15696.0	1.31
185.1	25429.0	2.12
186.1	11339.0	0.95
187.1	14306.0	1.19
189.1	15603.0	1.30
199.1	33648.0	2.81
200.1	11154.0	0.93
201.1	20022.0	1.67
205.1	62477.0	5.21
206.1	138180.0	11.53
207.2	16160.0	1.35
228.1	21660.0	1.81
234.2	257233.0	21.46
235.2	38005.0	3.17
257.1	1977.0	0.16
284.2	3862.0	0.32
316.3	5098.0	0.43
393.3	7106.0	0.59
394.3	19404.0	1.62
395.3	16747.0	1.40
396.3	4758.0	0.40

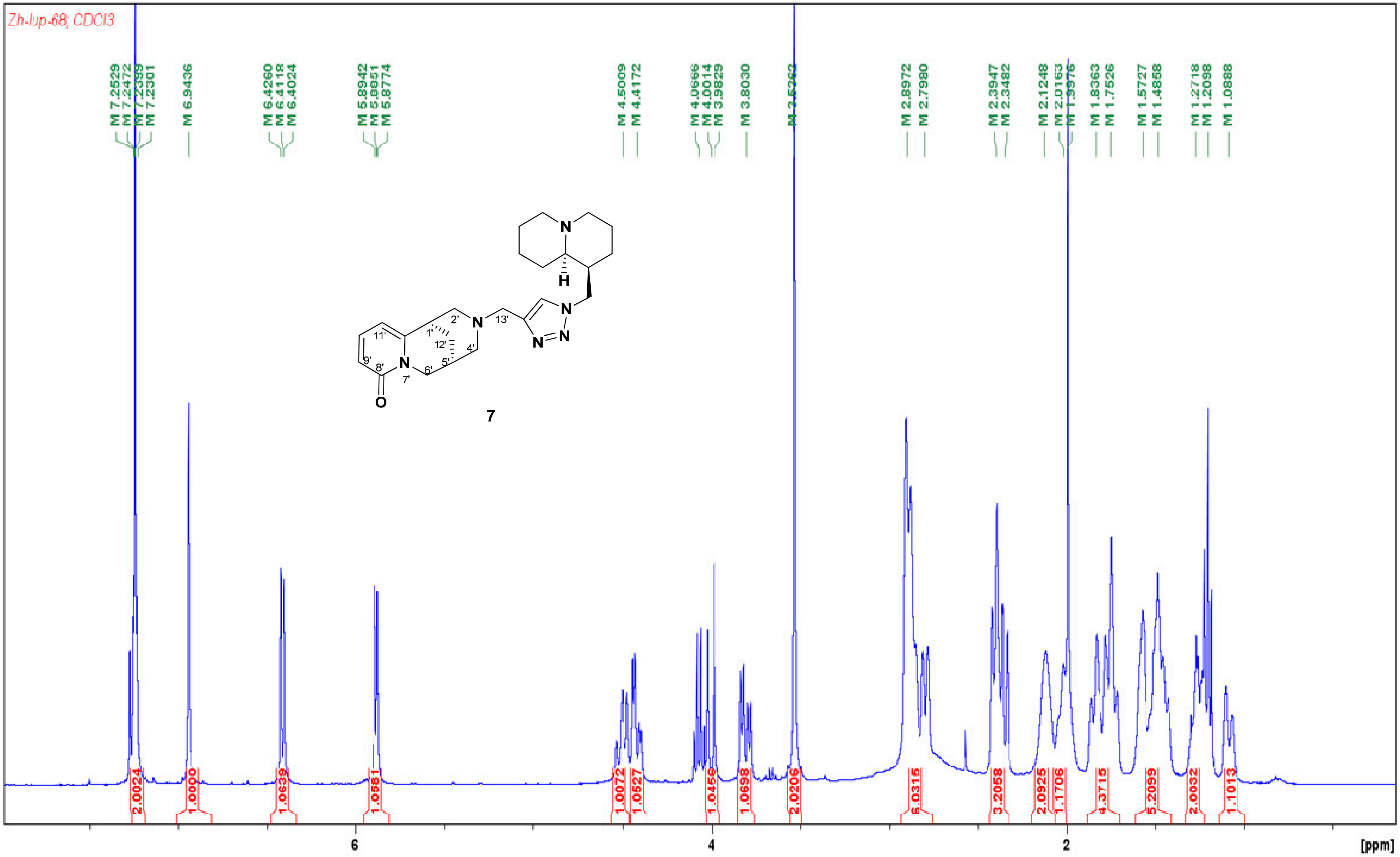

Figure S10. ^{1}H NMR spectrum of (1*R*,5*S*)-3-(1-(((1*S*,9a*R*)-octahydro-1*H*-quinolizin-1-yl)methyl)-1*H*-1,2,3-triazol-4-yl)methyl-1,2,3,4,5,6-hexahydro-8*H*-1,5-methanopyrido[1,2-*a*][1,5]diazocine-8 one (**7**) ($CDCl_3$, 400 MHz).

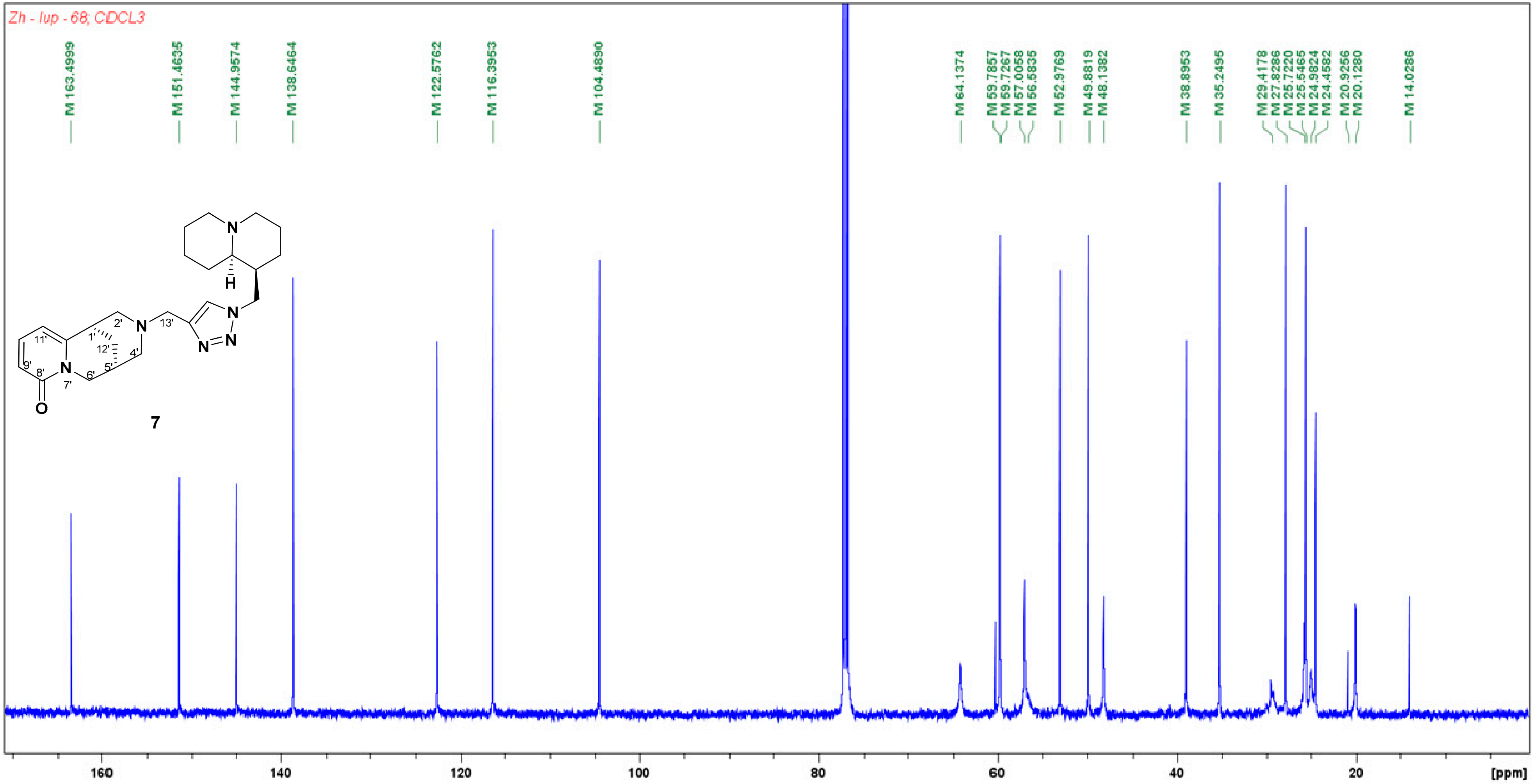

Figure S11. ^{13}C NMR spectrum of (1*R*,5*S*)-3-(1-(((1*S*,9a*R*)-octahydro-1*H*-quinolizin-1-yl)methyl)-1*H*-1,2,3-triazol-4-yl)methyl-1,2,3,4,5,6-hexahydro-8*H*-1,5-methanopyrido[1,2-*a*][1,5]diazocine-8 one (**7**) ($CDCl_3$, 125 MHz).

Figure S12. HR-MS spectrum of(1*R*,5*S*)-3-(1-(((1*S*,9a*R*)-octahydro-1*H*-quinolizin-1-yl)methyl)-1*H*-1,2,3-triazol-4-yl)methyl-1,2,3,4,5,6-hexahydro-8*H*-1,5-methanopyrido[1,2-*a*][1,5]diazocine-8 one (**7**).

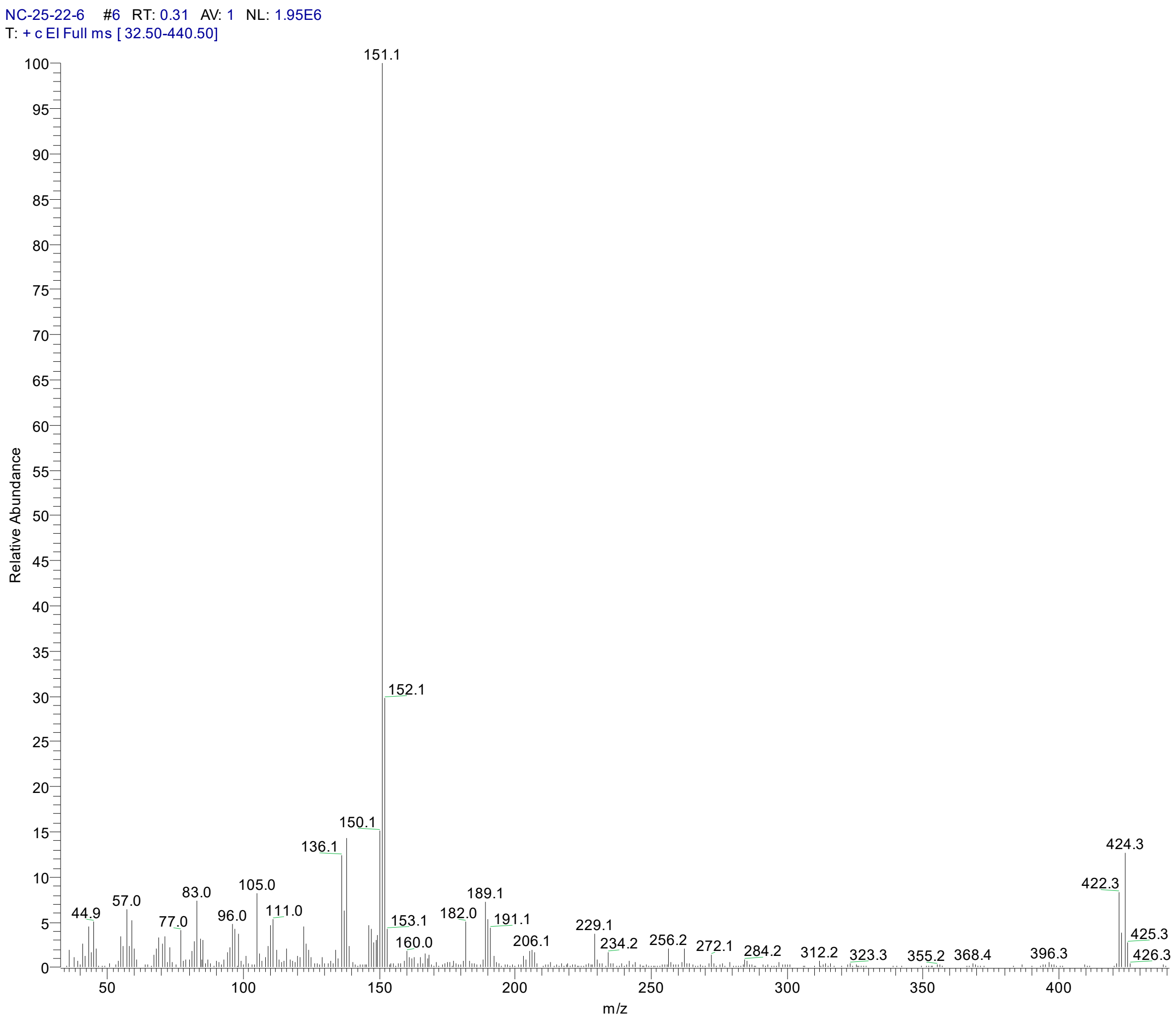

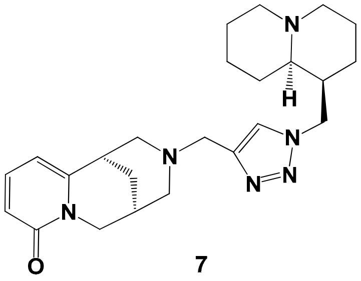

7

Tsource=100°C
Tprobe=280°C

Calculated: m/z= 422.2789
(C24 H34 O1 N6) $^{+\cdot}$
Found: m/z= 422.2790

1 C24 H34 O1 N6

RDB	(ppm)
11.0	0.328

Isotope	min	max
12 C	18	28
1 H	30	45
16 O	0	3
14 N	0	8

m/z	Intensity	Relative
42.9	86021.0	4.42
44.9	95422.0	4.90
57.0	124176.0	6.37
59.0	99589.0	5.11
83.0	142668.0	7.32
96.0	92598.0	4.75
97.0	80580.0	4.14
105.0	158543.0	8.14
110.0	88603.0	4.55
111.0	102963.0	5.29
122.0	84781.0	4.35
136.1	239502.0	12.29
137.1	119837.0	6.15
138.1	277037.0	14.22
146.0	87811.0	4.51
147.0	80959.0	4.16
150.1	292120.0	15.00
151.1	1948081.0	100.00
152.1	577113.0	29.62
153.1	80545.0	4.13
160.0	34057.0	1.75
167.0	26756.0	1.37
168.1	24897.0	1.28
182.0	97453.0	5.00
189.1	137813.0	7.07
190.1	102412.0	5.26
191.1	83094.0	4.27
192.1	23278.0	1.19
205.1	33230.0	1.71
206.1	36846.0	1.89
207.1	30303.0	1.56
229.1	71041.0	3.65
234.2	30097.0	1.54
256.2	39635.0	2.03
262.1	37122.0	1.91
272.1	25448.0	1.31
422.3	160437.0	8.24
423.3	73555.0	3.78
424.3	244461.0	12.55
425.3	52824.0	2.71

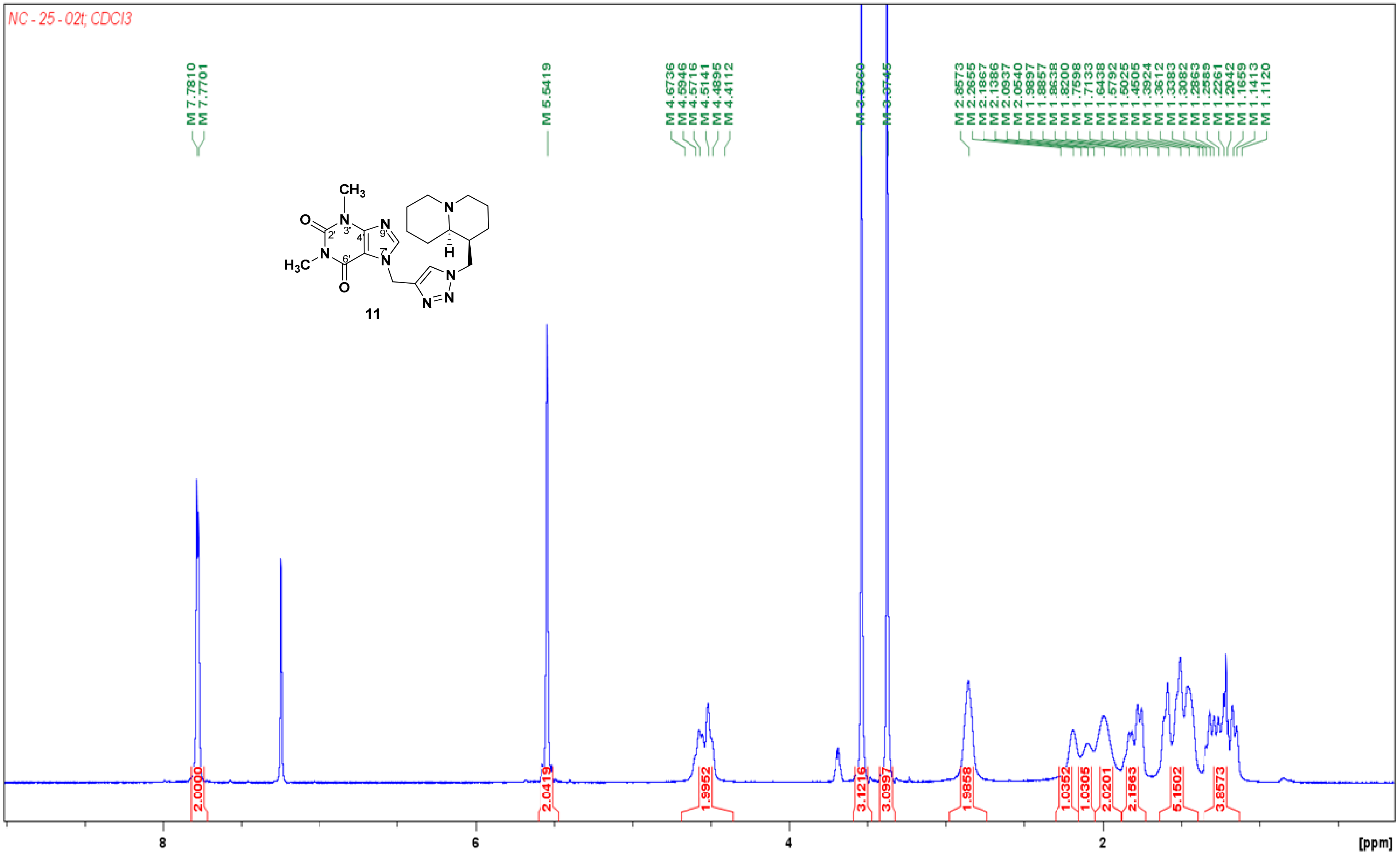

Figure S13. ^{1}H NMR spectrum of 1,3-dimethyl-7-((1-(((1*S*,9a*R*)-octahydro-1*H*-quinolizin-1-yl)methyl)-1*H*-1,2,3-triazol-4-yl) methyl)-1*H*-purine-2,6(3*H*,7*H*)-dione (**11**) ($CDCl_3$, 500 MHz).

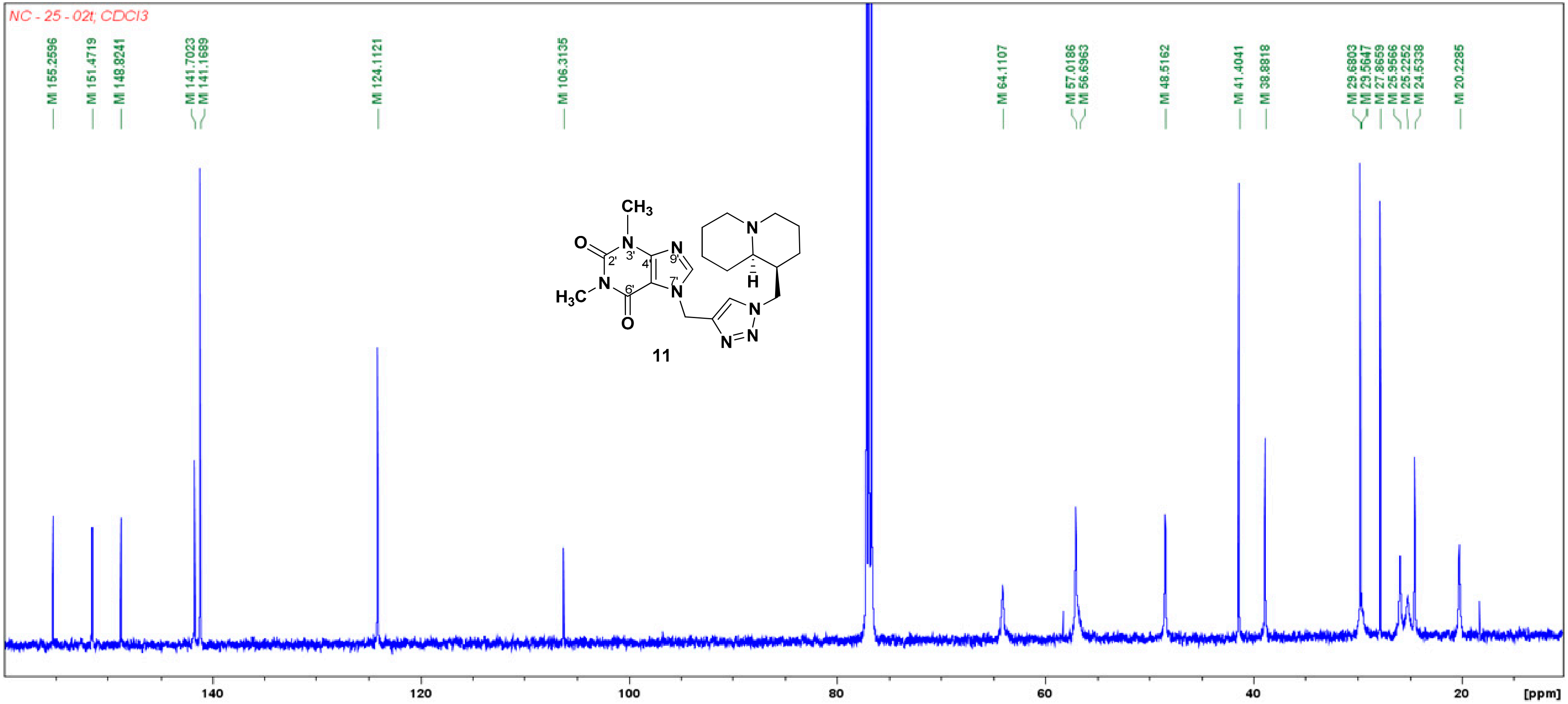

Figure S14. ^{13}C NMR spectrum of 1,3-dimethyl-7-((1-(((1*S*,9a*R*)-octahydro-1*H*-quinolizin-1-yl)methyl)-1*H*-1,2,3-triazol-4-yl) methyl)-1*H*-purine-2,6(3*H*,7*H*)-dione (**11** ($CDCl_3$, 125 MHz).

Figure S15. HR-MS spectrum of 1,3-dimethyl-7-((1-(((1*S*,9a*R*)-octahydro-1*H*-quinolizin-1-yl)methyl)-1*H*-1,2,3-triazol-4-yl) methyl)-1*H*-purine-2,6(3*H*,7*H*)-dione (**11**)

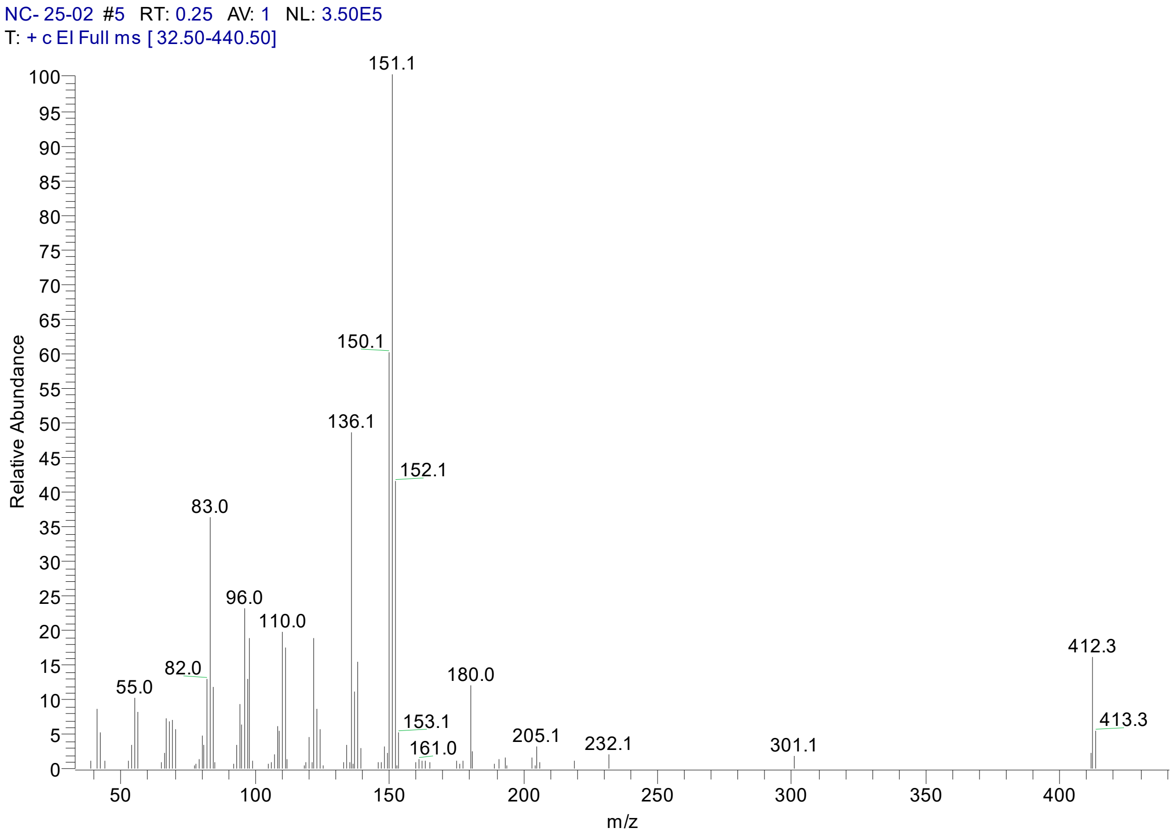

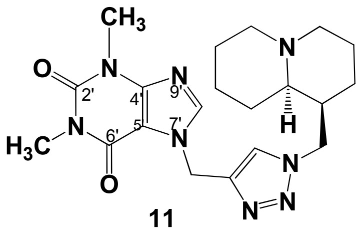

11

Tsource=110°C
Tprobe=300°C

Calculated: m/z= 412.2330
(C20 H28 O2 N8)$^{+\cdot}$
Found: m/z= 412.2334

**1**	**C20 H28 O2 N8**	
**RDB**		**(ppm)**
**11.0**		**1.033**
**Isotope**	**min**	**max**
**12 C**	**15**	**25**
**1 H**	**20**	**35**
**16 O**	**0**	**4**
**14 N**	**0**	**10**

m/z	Intensity	Relative
40.9	30044.0	8.59
55.0	35580.0	10.17
56.0	27960.0	7.99
82.0	44818.0	12.81
83.0	126524.0	36.17
84.0	40775.0	11.66
94.0	32470.0	9.28
96.0	80865.0	23.12
97.0	45129.0	12.90
98.0	65190.0	18.64
110.0	68798.0	19.67
111.1	60929.0	17.42
122.0	65221.0	18.64
123.1	29391.0	8.40
136.1	169351.0	48.41
137.1	38629.0	11.04
138.1	53682.0	15.35
150.1	210095.0	60.06
151.1	349807.0	100.00
152.1	144936.0	41.43
163.1	3390.0	0.97
165.1	2736.0	0.78
175.0	3856.0	1.10
175.1	3918.0	1.12
176.1	1803.0	0.52
177.1	3483.0	1.00
180.0	41614.0	11.90
181.0	8273.0	2.37
189.0	1803.0	0.52
191.1	4261.0	1.22
193.0	4914.0	1.40
194.0	1586.0	0.45
203.1	5100.0	1.46
205.1	10885.0	3.11
206.1	2892.0	0.83
219.1	3856.0	1.10
232.1	6593.0	1.88
301.1	6002.0	1.72
411.3	7868.0	2.25
412.3	56108.0	16.04
413.3	18754.0	5.36

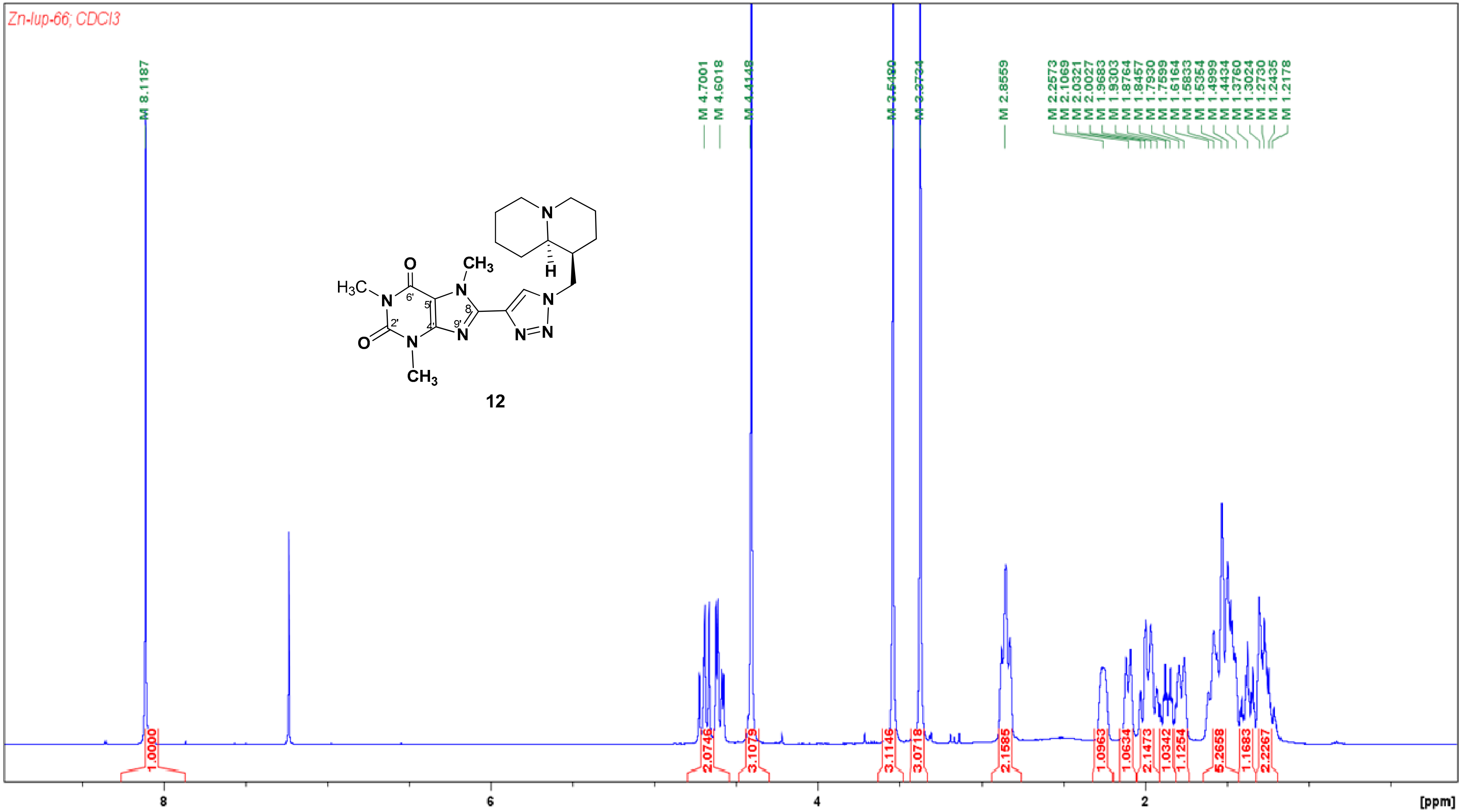

Figure S16. ^{1}H NMR spectrum of 1,3,7-trimethyl-8-(1-(((1*S*,9a*R*)-octahydro-1*H*-quinolizin-1-yl)methyl)-1*H*-1,2,3-triazol-4-yl)-1*H*-purine-2,6(3H,7*H*)-dione (**12**) ($CDCl_3$, 400 MHz).

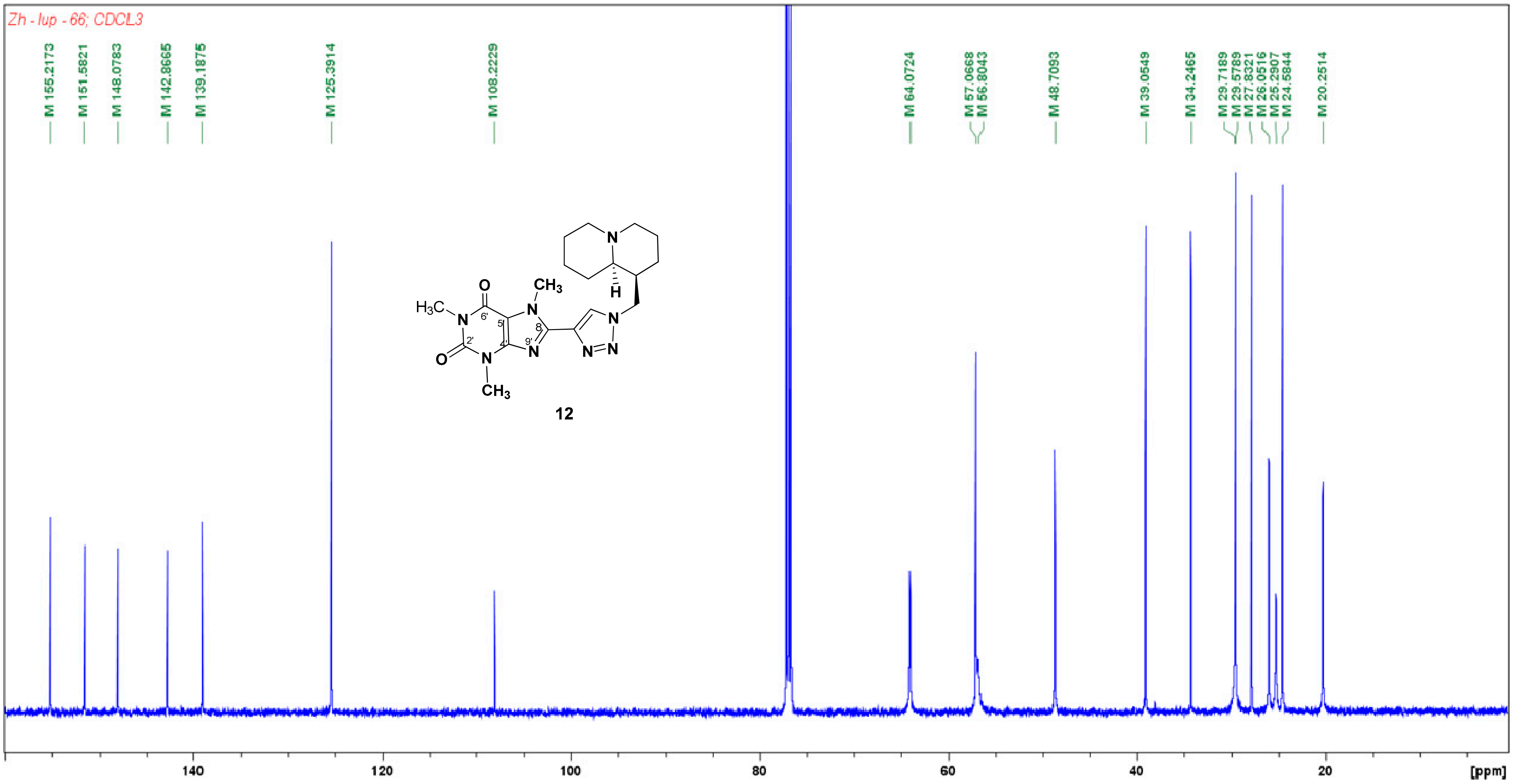

Figure S17. ^{13}C NMR spectrum of 1,3,7-trimethyl-8-(1-(((1*S*,9a*R*)-octahydro-1*H*-quinolizin-1-yl)methyl)-1*H*-1,2,3-triazol-4-yl)-1*H*-purine-2,6(3H,7*H*)-dione (**12**) ($CDCl_3$, 125 MHz).

Figure S18. HR-MS spectrum of 1,3,7-trimethyl-8-(1-(((1*S*,9a*R*)-octahydro-1*H*-quinolizin-1-yl)methyl)-1*H*-1,2,3-triazol-4-yl)-1*H*-purine-2,6-dione (**12**)

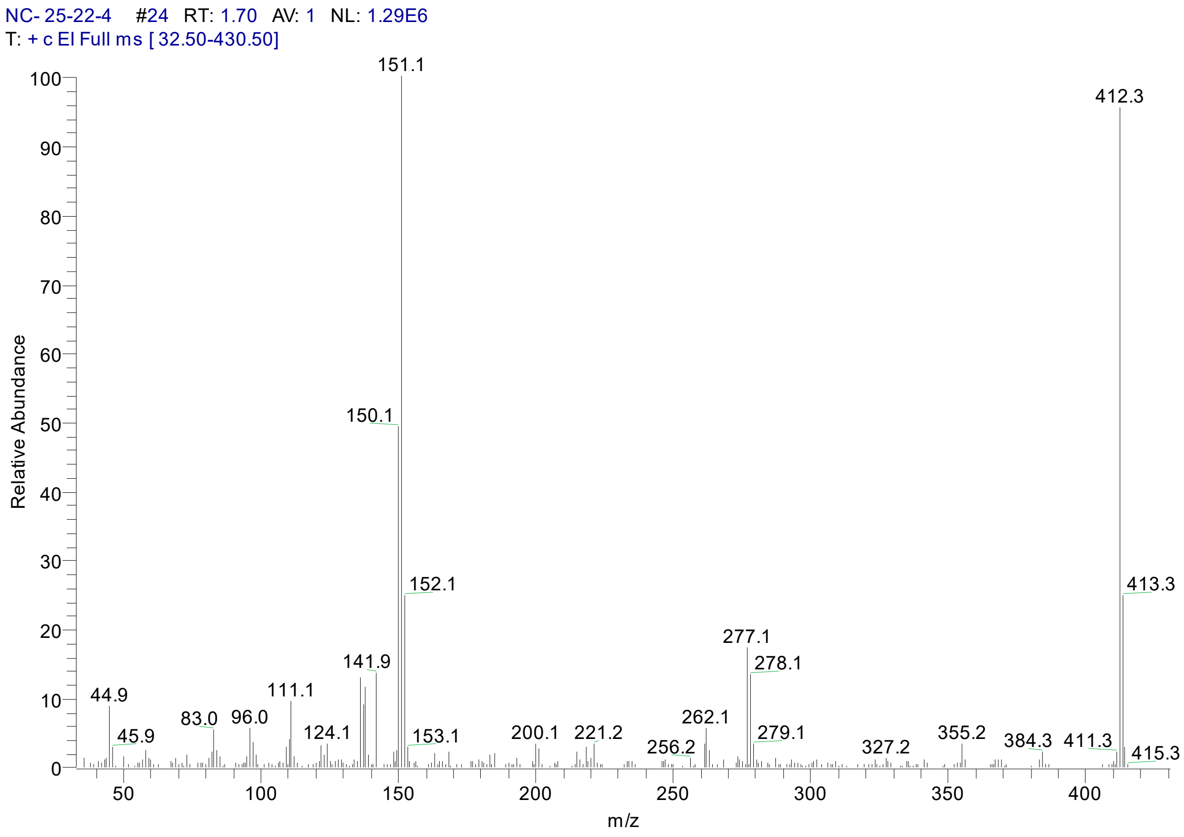

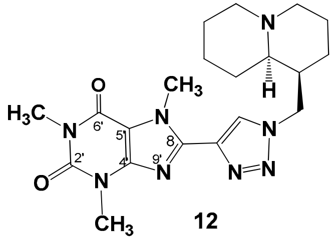

12

Tsource=100°C
Tprobe=270°C
Calculated: m/z= 412.2330
(C20 H28 O2 N8)$^{+\cdot}$
Found: m/z= 412.2329

**1**	**C20 H28 O2 N8**
**RDB**	**(ppm)**
**11.0**	**-0.179**

Isotope	min	max
12 C	15	25
1 H	22	35
16 O	0	4
14 N	0	10

m/z	Intensity	Relative
44.9	114280.0	8.83
45.9	37482.0	2.89
58.0	31328.0	2.42
83.0	68417.0	5.28
84.0	31917.0	2.47
96.0	72837.0	5.63
97.0	46092.0	3.56
109.0	35583.0	2.75
110.0	53261.0	4.11

m/z	Intensity	Relative
111.1	121711.0	9.40
122.0	39250.0	3.03
124.1	44193.0	3.41
136.1	168589.0	13.02
137.1	117750.0	9.09
138.1	149439.0	11.54
141.9	176085.0	13.60
150.1	639396.0	49.38
151.1	1294799.0	100.00
152.1	320811.0	24.78
153.1	36434.0	2.81
200.1	43113.0	3.33
201.0	33619.0	2.60
218.1	37220.0	2.87
221.2	41967.0	3.24
261.1	43244.0	3.34
262.1	72084.0	5.57
263.1	31262.0	2.41
277.1	224535.0	17.34
278.1	173598.0	13.41
279.1	43964.0	3.40
287.1	15254.0	1.18
302.2	13912.0	1.07
327.2	17415.0	1.34
341.2	13879.0	1.07
355.1	12963.0	1.00
355.2	41509.0	3.21
356.3	13061.0	1.01
367.2	14076.0	1.09
368.2	13356.0	1.03
383.3	14371.0	1.11
384.3	27432.0	2.12
411.3	29200.0	2.26
412.3	1236890.0	95.53
413.3	321531.0	24.83
414.3	38399.0	2.97

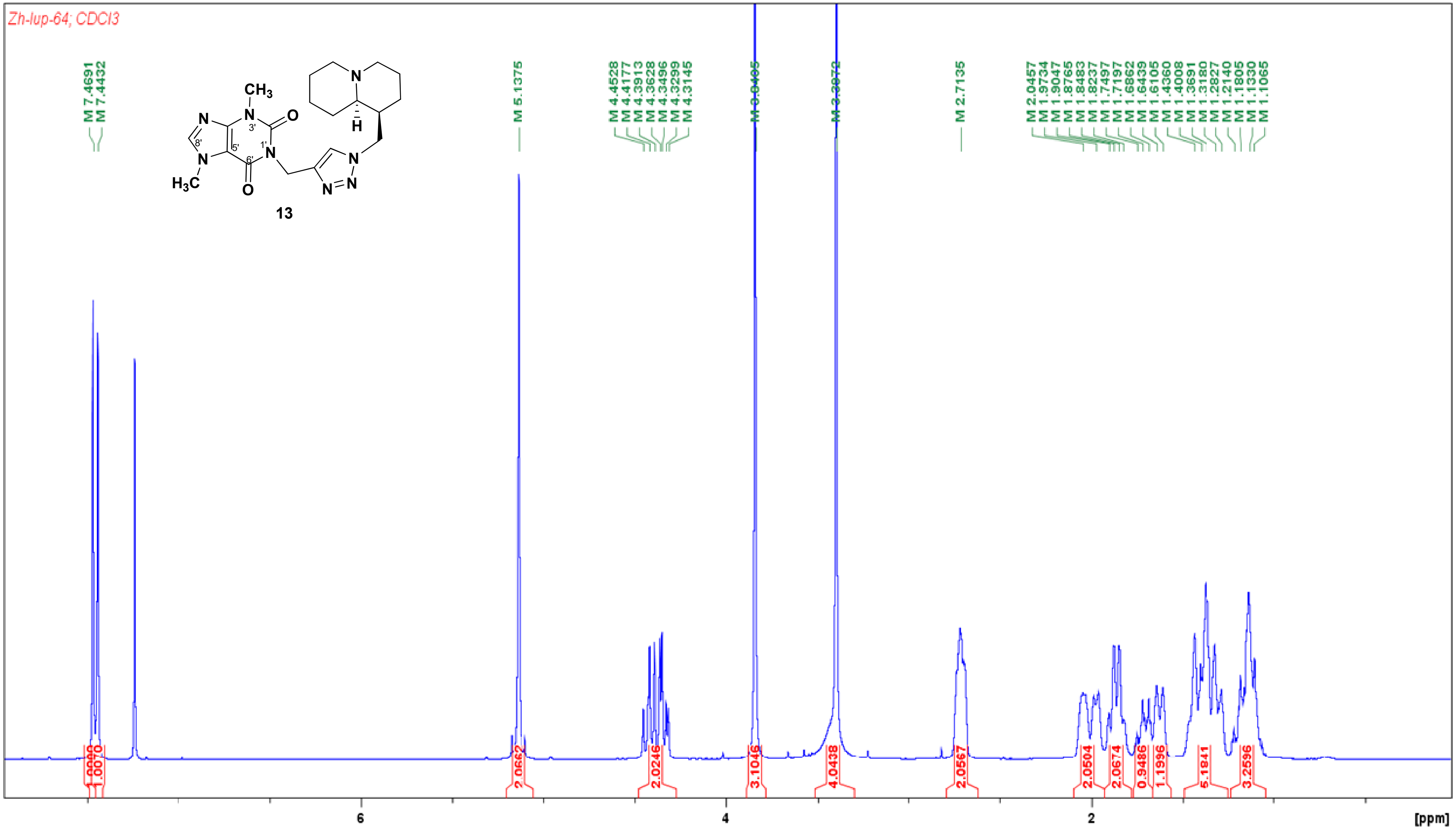

Figure S19. ^{1}H NMR spectrum of 3,7-dimethyl-1-((1-(((1*S*,9a*R*)-octahydro-1*H*-quinolizin-1-yl)methyl)-1*H*-1,2,3-triazol-4-yl)methyl)-1*H*-purine-2,6(3*H*,7*H*)-dione (**13**) ($CDCl_3$, 400 MHz).

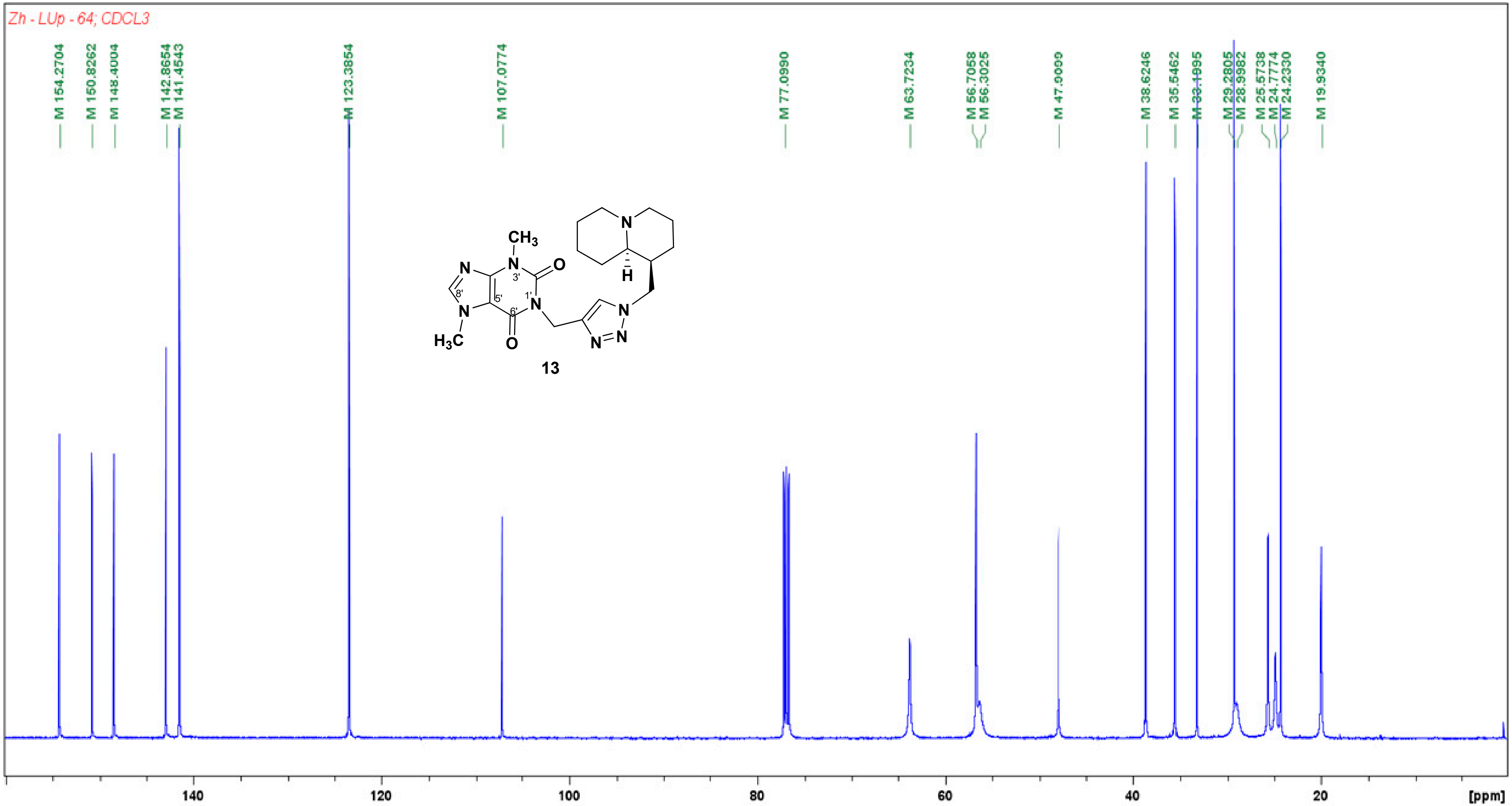

Figure S20. ^{13}C NMR spectrum of 3,7-dimethyl-1-((1-((((1*S*,9a*R*)-octahydro-1*H*-quinolizin-1-yl)methyl)-1*H*-1,2,3-triazol-4-yl)methyl)-1*H*-purine-2,6(3*H*,7*H*)-dione (**13**) ($CDCl_3$, 125 MHz).

Figure S21. HR-MS spectrum of 3,7-dimethyl-1-((1-(((1*S*,9a*R*)-octahydro-1*H*-quinolizin-1-yl)methyl)-1*H*-1,2,3-triazol-4-yl)methyl)-1*H*-purine-2,6(3*H*,7*H*)-dione (**13**) .

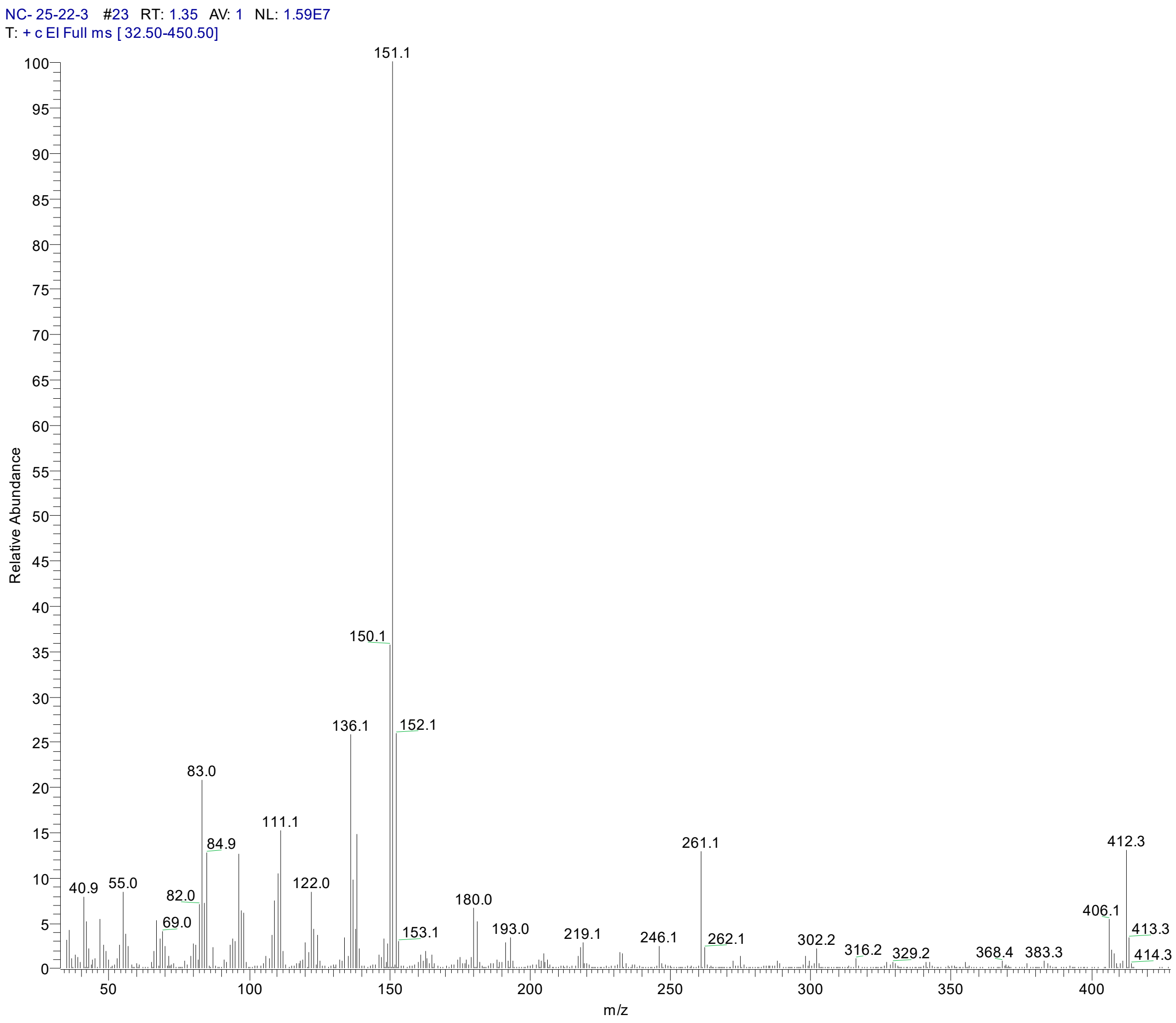

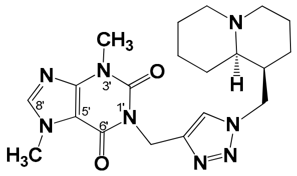

13

Tsource=110˚C
Tprobe=270˚C
Calculated: m/z=412.2330
(C20 H28 O2 N8) $^{+\cdot}$
Found: m/z= 412.2326

1	C20 H28 O2 N8
RDB	(ppm)
11.0	-0.907

Isotope	min	max
12 C	16	25
1 H	20	35
16 O	0	4
14 N	0	10

m/z	Intensity	Relative
40.9	1238564.0	7.81
55.0	1329211.0	8.38
82.0	1118255.0	7.05
82.9	2995528.0	18.88
83.0	3291690.0	20.74
84.0	1136298.0	7.16

m/z	Intensity	Relative
84.9	2020297.0	12.73
96.0	1996506.0	12.58
97.0	1005015.0	6.33
98.0	951162.0	5.99
109.0	1169741.0	7.37
110.0	1639205.0	10.33
111.1	2405170.0	15.16
122.0	1328381.0	8.37
136.1	4085842.0	25.75
137.1	1544778.0	9.74
138.1	2334165.0	14.71
150.1	5667045.0	35.71
151.1	15868160.0	100.00
152.1	4108373.0	25.89
161.1	218487.0	1.38
163.0	294902.0	1.86
180.0	1044760.0	6.58
181.0	810719.0	5.11
191.1	437834.0	2.76
193.0	534014.0	3.37
205.0	244737.0	1.54
218.1	353120.0	2.23
219.1	440877.0	2.78
232.1	267299.0	1.68
233.1	255157.0	1.61
246.1	370948.0	2.34
261.1	2042675.0	12.87
262.1	357423.0	2.25
302.2	344236.0	2.17
406.1	861314.0	5.43
407.1	316941.0	2.00
408.1	245321.0	1.55
412.3	2058228.0	12.97
413.3	538287.0	3.39

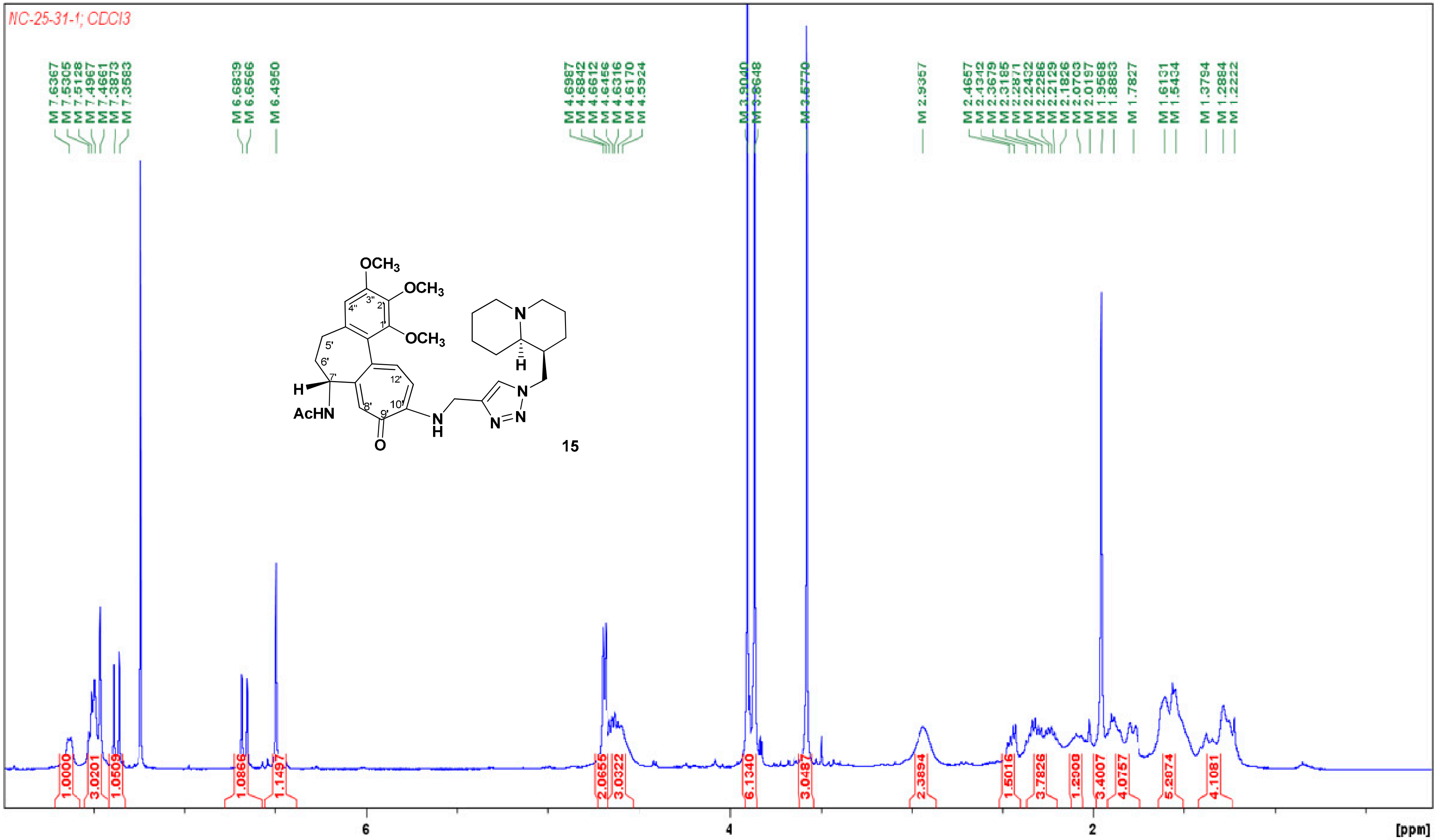

Figure S22. ^{1}H NMR spectrum of 1*R*,5*S*)-10-(1-(((1*S*,9a*R*)-octahydro-1*H*-quinolizin-1-yl)methyl)-1*H*-1,2,3-triazol-4-yl)methylamino-N-[(7*S*)-acetylamino-1,2,3,-trimethoxy-6,7-dihydro-5*H*-benzo[*a*]heptalen-9-one (**15**) ($CDCl_3$, 400 MHz).

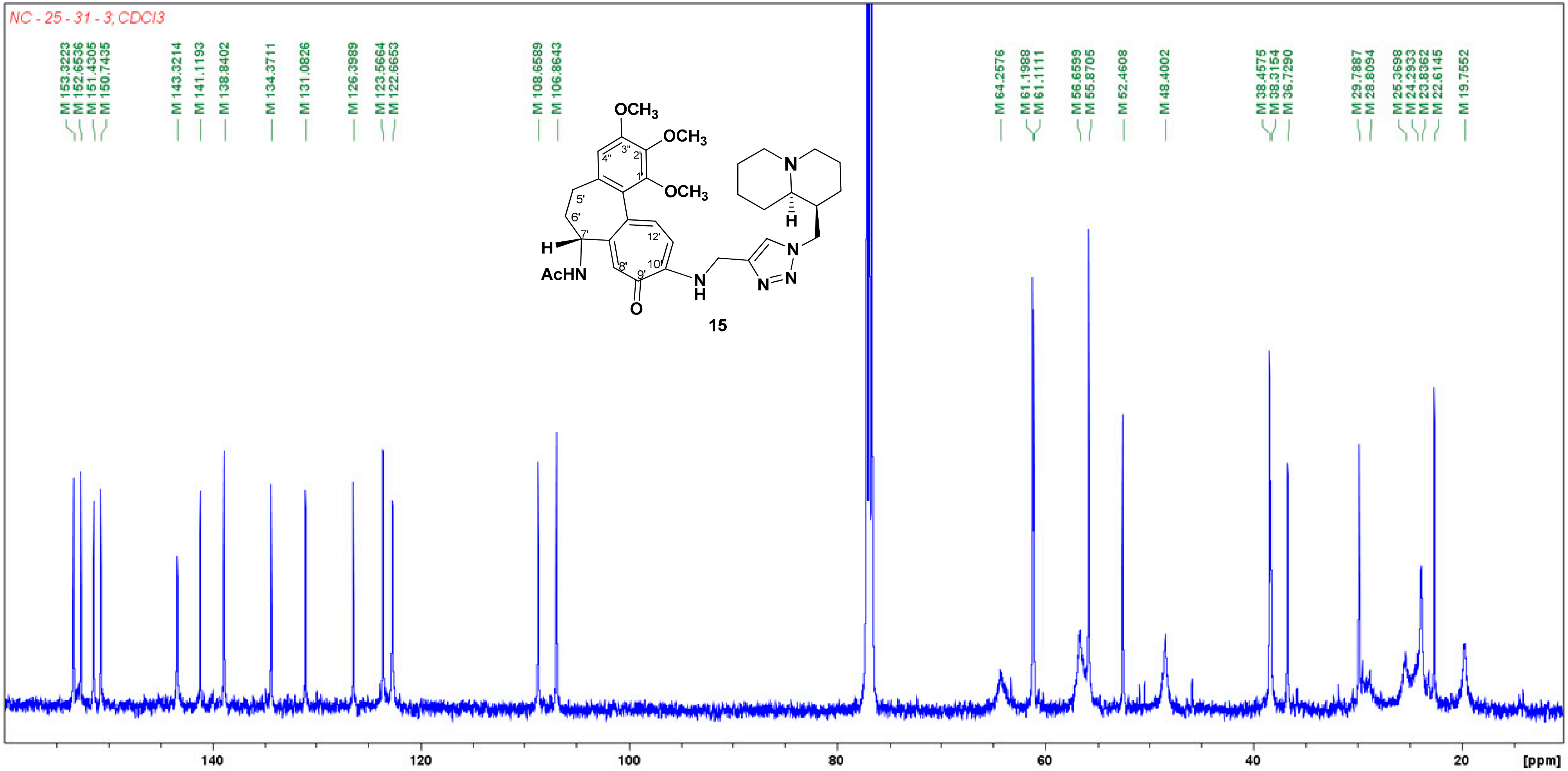

Figure S23. ^{13}C NMR spectrum of of 1*R*,5*S*)-10-(1-(((1*S*,9a*R*)-octahydro-1*H*-quinolizin-1-yl)methyl)-1*H*-1,2,3-triazol-4-yl)methylamino-N-[(7*S*)-acetylamino-1,2,3,-trimethoxy-6,7-dihydro-5*H*-benzo[*a*]heptalen-9-one (**15**) (CDCl3, 125 MHz).

Figure S24. HR-MS spectrum of of 1*R*,5*S*)-10-(1-(((1*S*,9a*R*)-octahydro-1*H*-quinolizin-1-yl)methyl)-1*H*-1,2,3-triazol-4-yl)methylamino-N-[(7*S*)-acetylamino-1,2,3,-trimethoxy-6,7-dihydro-5*H*-benzo[*a*]heptalen-9-one (**15**).

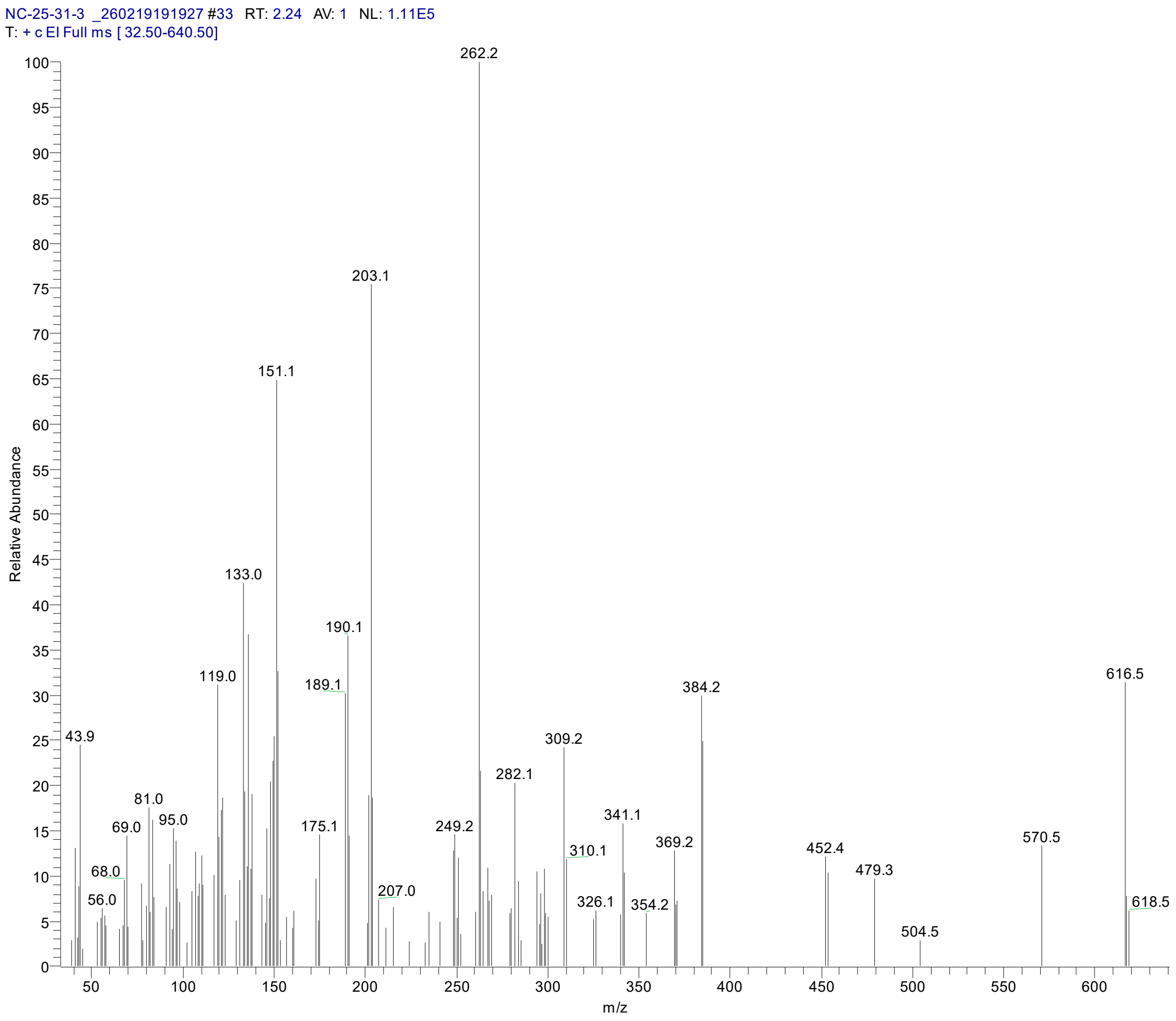

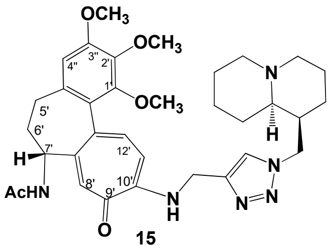

15

Tsource=115°C
Tprobe=340°C
Calculated: m/z= 616.3368
(C24 H34 O1 N6) $^{+\cdot}$
Found: m/z= 616.3367

1	C24 H34 O1 N6
RDB	(ppm)
11.0	-0.114

Isotope	min	max
12 C	30	40
1 H	35	55
16 O	0	7
14 N	0	8

m/z	Intensity	Relative
43.9	27171.0	24.40
81.0	19445.0	17.46
119.0	34545.0	31.02
122.0	20630.0	18.53

m/z	Intensity	Relative
133.0	47100.0	42.29
134.0	21377.0	19.20
136.1	40822.0	36.66
138.1	21157.0	19.00
148.1	22606.0	20.30
149.1	25239.0	22.66
150.1	28268.0	25.38
151.1	72164.0	64.80
152.1	36301.0	32.60
189.1	33492.0	30.07
190.1	40647.0	36.50
202.1	21026.0	18.88
203.1	83972.0	75.40
204.1	20674.0	18.56
262.2	111363.0	100.00
263.2	23923.0	21.48
282.1	22474.0	20.18
284.1	10359.0	9.30
294.1	11500.0	10.33
296.1	8823.0	7.92
298.1	11895.0	10.68
309.2	26820.0	24.08
310.1	13080.0	11.75
341.1	17470.0	15.69
342.1	11412.0	10.25
369.2	14178.0	12.73
370.1	7506.0	6.74
371.2	7901.0	7.09
384.2	33272.0	29.88
385.2	27654.0	24.83
452.4	13388.0	12.02
453.3	11456.0	10.29
479.3	10622.0	9.54
504.5	3116.0	2.80
570.5	14705.0	13.20
616.5	34853.0	31.30
617.6	8559.0	7.69
618.5	6803.0	6.11

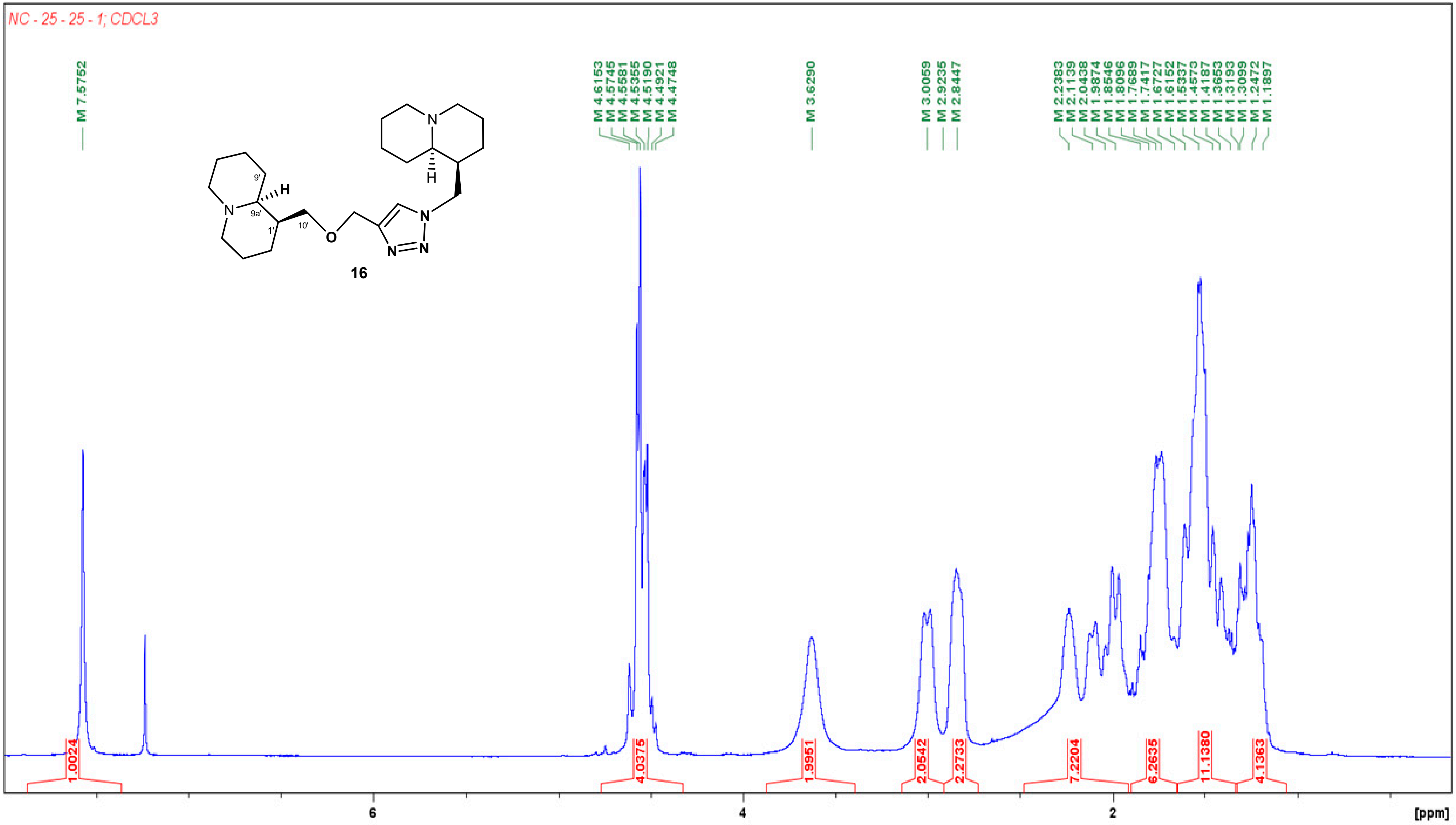

Figure S25. ^{1}H NMR spectrum of (1*S*,9a*R*)-1-((4-((((1*S*,9a*S*)-octahydro-1*H*-quinolizin-1-yl)methoxy)methyl)-1*H*-1,2,3-triazol-1-yl)methyl)octahydro-1*H*-quinolizine (**16**) ($CDCl_3$, 300 MHz).

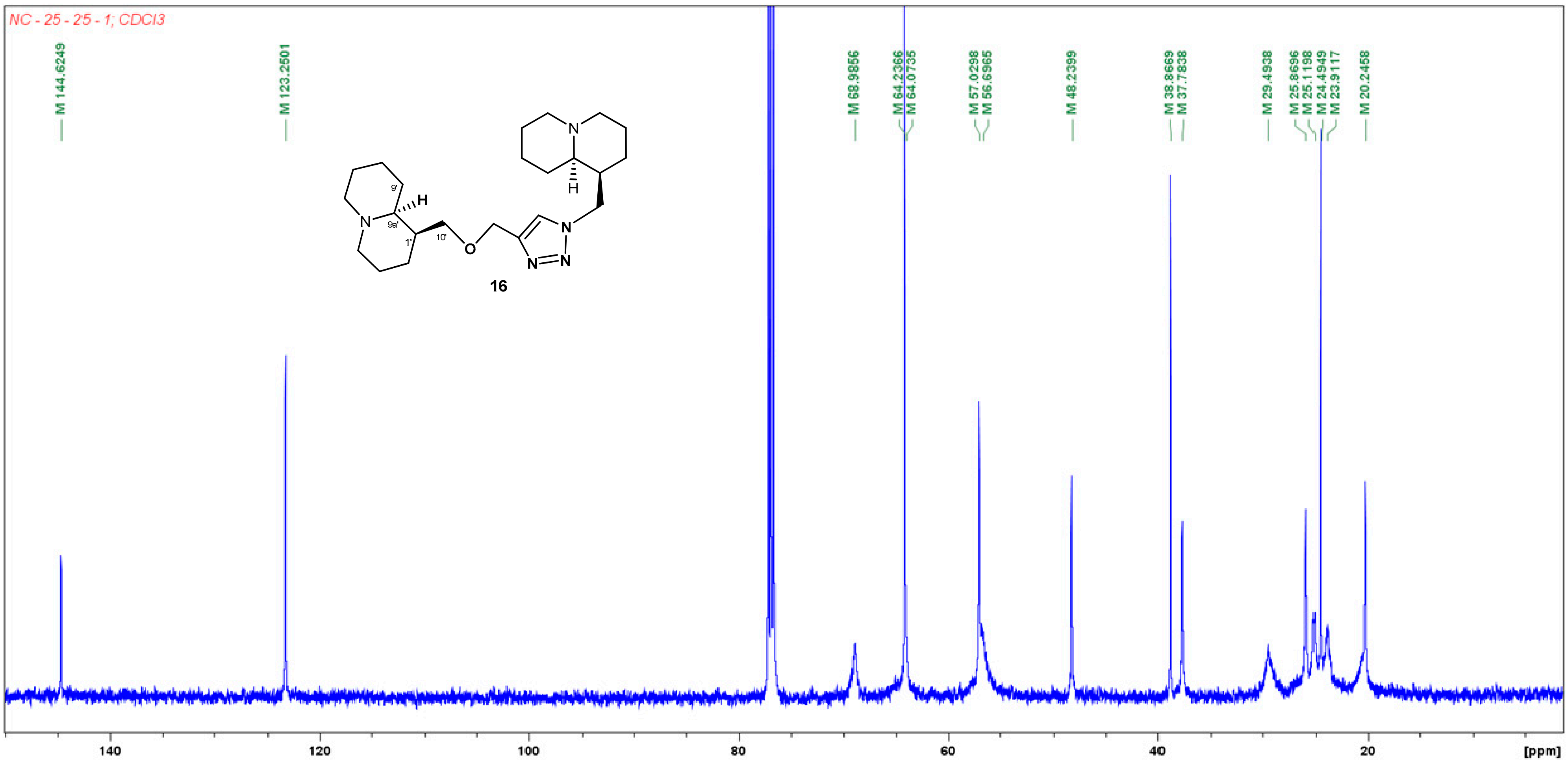

Figure S26. ^{13}C NMR spectrum of (1*S*,9a*R*)-1-((4-((((1*S*,9a*S*)-octahydro-1*H*-quinolizin-1-yl)methoxy)methyl)-1*H*-1,2,3-triazol-1-yl)methyl)octahydro-1*H*-quinolizine (**16**) ($CDCl_3$, 125 MHz).

Figure S27. HR-MS spectrum of (1*S*,9a*R*)-1-((4-((((1*S*,9a*S*)-octahydro-1*H*-quinolizin-1-yl)methoxy)methyl)-1*H*-1,2,3-triazol-1-yl)methyl)octahydro-1*H*-quinolizine (**16**)

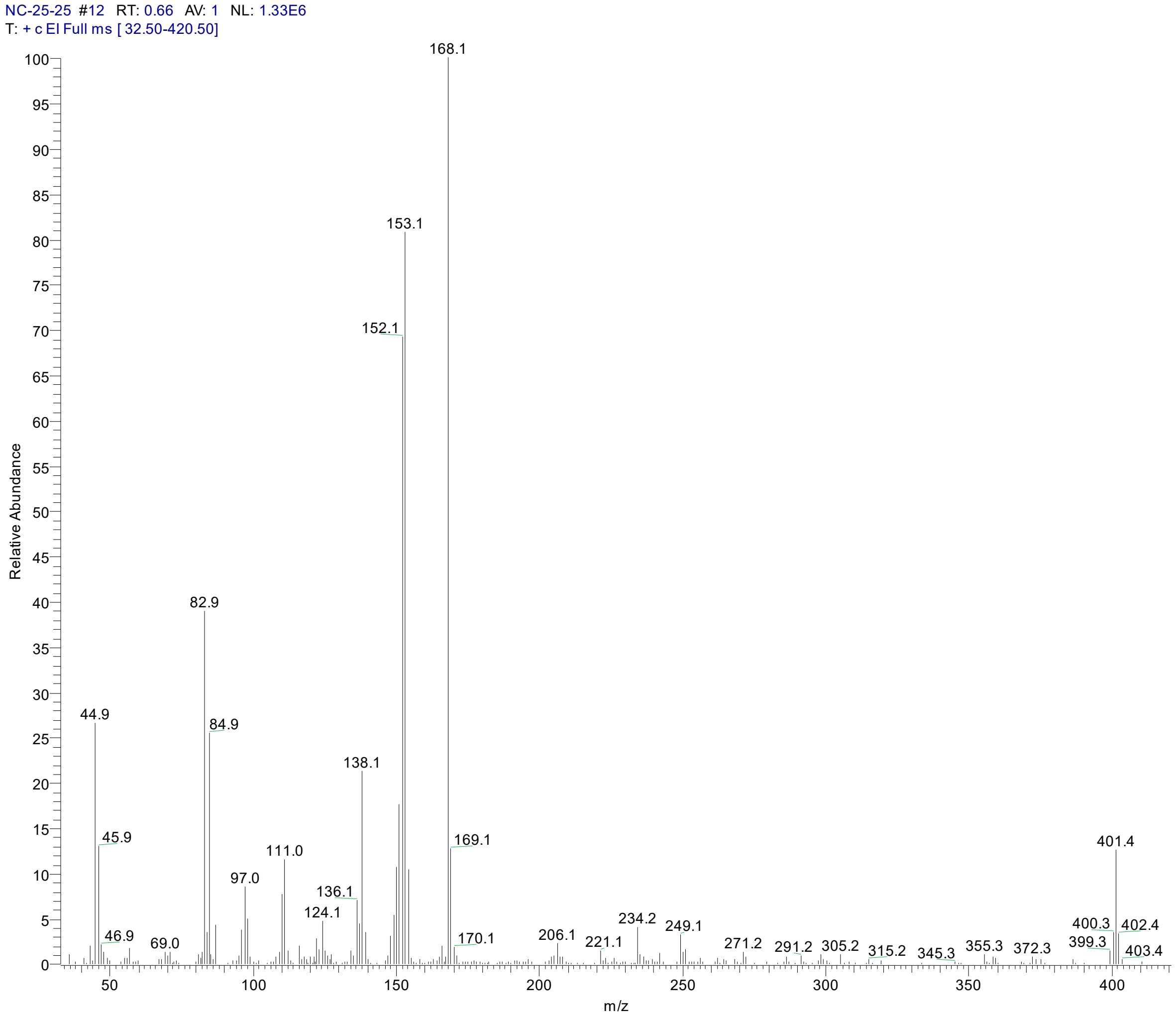

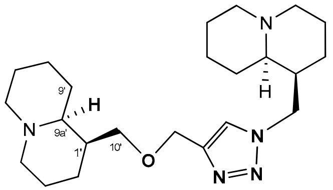

16

Tsource=100°C

Tprobe=260°C

Calculated: m/z= 401.3149

(C23 H39 O1 N5) $^{+\cdot}$

Found: m/z= 401.3150

1 C23 H39 O1 N5

RDB	(ppm)
7.0	0.217

Isotope	min	max
12 C	18	28
1 H	30	50
16 O	0	3
14 N	0	7

m/z	Intensity	Relative
44.9	352499.0	26.51
45.9	171802.0	12.92
82.9	517834.0	38.94
83.0	71644.0	5.39
84.9	338226.0	25.43
97.0	112534.0	8.46
98.0	66699.0	5.02
110.0	101402.0	7.62
111.0	153363.0	11.53

m/z	Intensity	Relative
124.1	62533.0	4.70
136.1	92633.0	6.97
138.1	282316.0	21.23
149.1	71644.0	5.39
150.1	140956.0	10.60
151.1	233683.0	17.57
152.1	921516.0	69.29
153.1	1075097.0	80.84
154.1	138748.0	10.43
168.1	1329894.0	100.00
169.1	169159.0	12.72
205.1	11660.0	0.88
206.1	29447.0	2.21
208.1	10541.0	0.79
221.1	18564.0	1.40
234.2	53422.0	4.02
235.2	12842.0	0.97
242.2	15765.0	1.19
249.1	41730.0	3.14
250.2	16791.0	1.26
251.2	20647.0	1.55
271.2	17351.0	1.30
291.2	11132.0	0.84
298.2	13619.0	1.02
305.2	13930.0	1.05
355.3	13744.0	1.03
358.3	9421.0	0.71
359.3	7307.0	0.55
372.3	10790.0	0.81
373.3	7058.0	0.53
375.3	6747.0	0.51
399.3	19030.0	1.43
400.3	45803.0	3.44
401.4	167231.0	12.57
402.4	43595.0	3.28

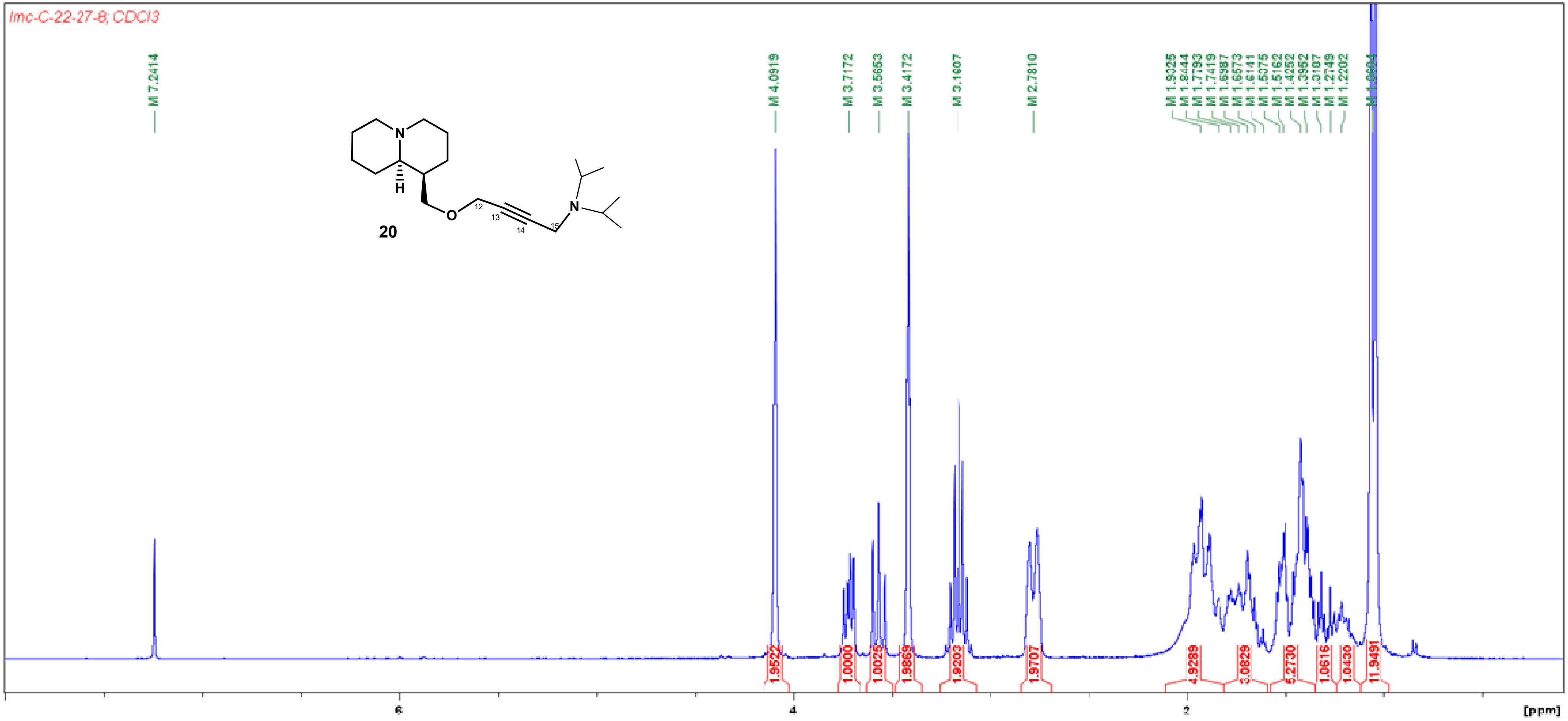

Figure S28. ^{1}H NMR spectrum of N,N-diisopropyl-4-(((1*R*,9a*R*)-octahydro-1H-quinolizin-1-yl)methoxy)but-2-yn-1-amine (**20**) ($CDCl_3$, 300 MHz).

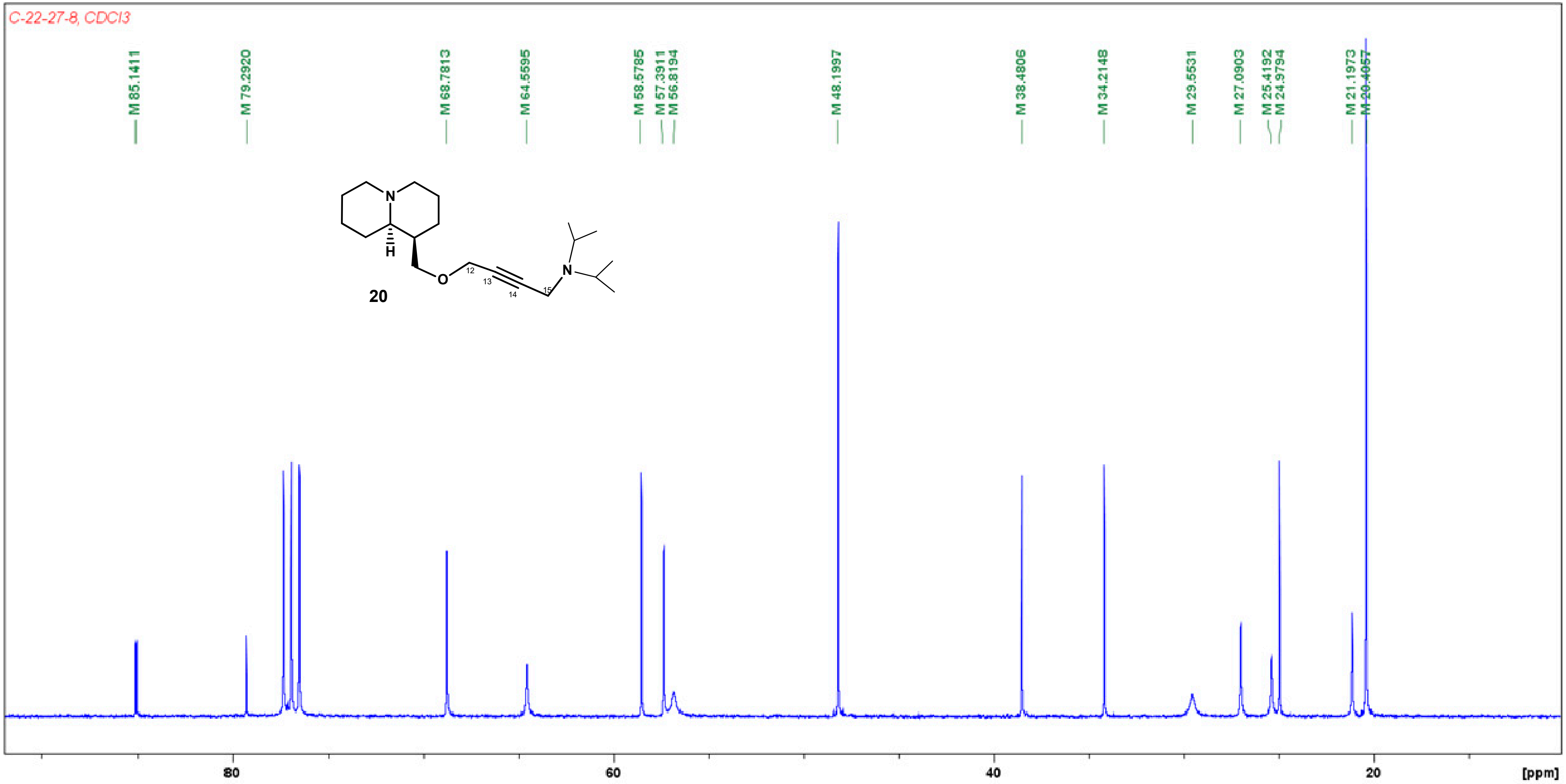

Figure S29. ^{13}C NMR spectrum of N,N-diisopropyl-4-(((1*R*,9a*R*)-octahydro-1*H*-quinolizin-1-yl)methoxy)but-2-yn-1-amine (**20**) ($CDCl_3$, 125 MHz).

Figure S30. HR-MS spectrum of N,N-diisopropyl-4-(((1*R*,9a*R*)-octahydro-1H-quinolizin-1-yl)methoxy)but-2-yn-1-amine (**20**)

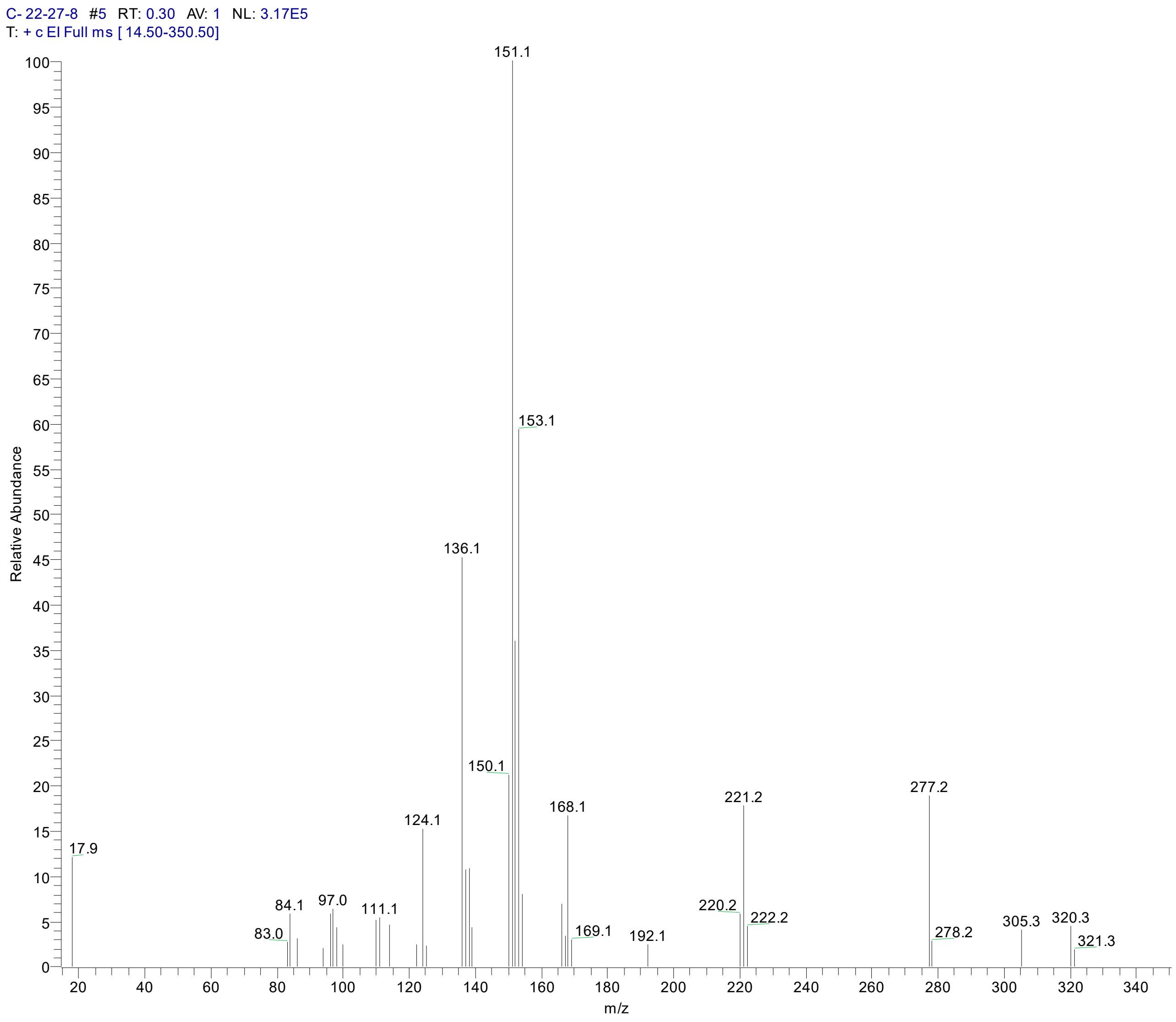

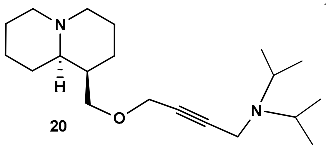

20

Tsource=80˚C
Tprobe=220˚C

Calculated
m/z= 320.2822
(C20 H36 O1 N2)$^{+\cdot}$

Found

m/z= 320.2819

Элементный состав
1 C20 H36 O1 N2

RDB	Ошибка (ppm)
4.0	-0.985

Интервал значений чисел атомов элементов:

Isotope	min	max
12 C	17	25
1 H	25	45
16 O	0	3
14 N	0	4

m/z	Intensity	Relative
17.9	38192.0	12.06
83.0	8338.0	2.63
84.1	18427.0	5.82
86.1	9643.0	3.04
96.0	18427.0	5.82
97.0	19892.0	6.28
98.1	13431.0	4.24
100.1	7320.0	2.31
110.1	16104.0	5.08
111.1	16963.0	5.36
114.1	14322.0	4.52
122.0	7447.0	2.35
124.1	47804.0	15.09
136.1	142936.0	45.13
137.1	33641.0	10.62
138.1	34118.0	10.77
139.1	13526.0	4.27
150.1	67028.0	21.16
151.1	316712.0	100.00
152.1	113718.0	35.91
153.1	187971.0	59.35
154.1	25239.0	7.97
166.1	21833.0	6.89
167.1	10598.0	3.35
168.1	52833.0	16.68
169.1	9070.0	2.86
192.1	7670.0	2.42
220.2	18364.0	5.80
221.2	56143.0	17.73
222.2	14131.0	4.46
277.2	59612.0	18.82
278.2	9007.0	2.84
305.3	12699.0	4.01
320.3	14163.0	4.47
321.3	5665.0	1.79

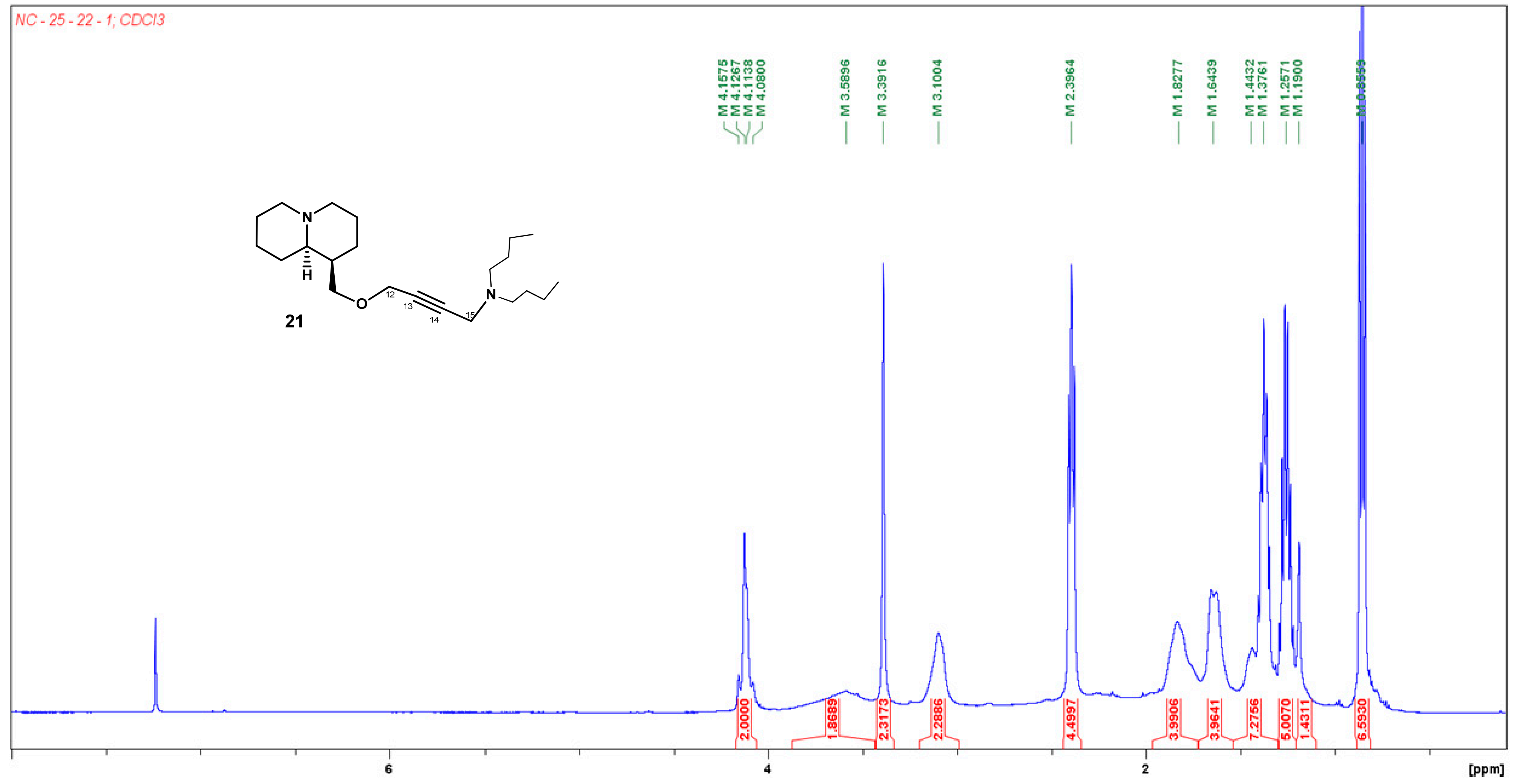

Figure S31. ^{1}H NMR spectrum of N,N-dibutyl-4-(((1*R*,9a*R*)-octahydro-1*H*-quinolizin-1-yl)methoxy)but-2-yn-1-amine (**21**) ($CDCl_3$, 500 MHz).

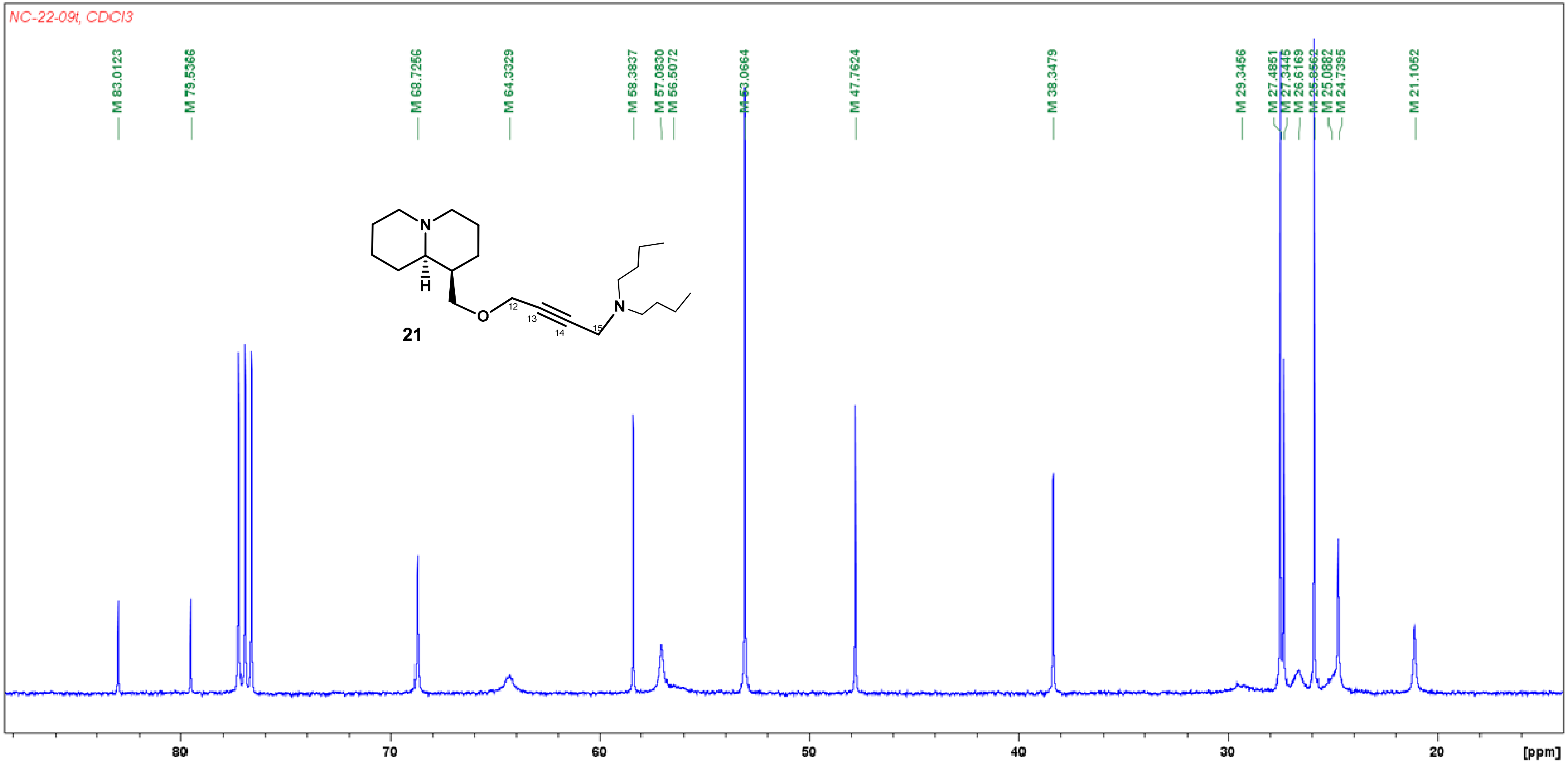

Figure S32. ^{13}C NMR spectrum of N,N-dibutyl-4-(((1*R*,9a*R*)-octahydro-1*H*-quinolizin-1-yl)methoxy)but-2-yn-1-amine (**21**) ($CDCl_3$, 125 MHz).

Figure S33. HR-MS spectrum of N,N-dibutyl-4-(((1*R*,9a*R*)-octahydro-1*H*-quinolizin-1-yl)methoxy)but-2-yn-1-amine (**21**).

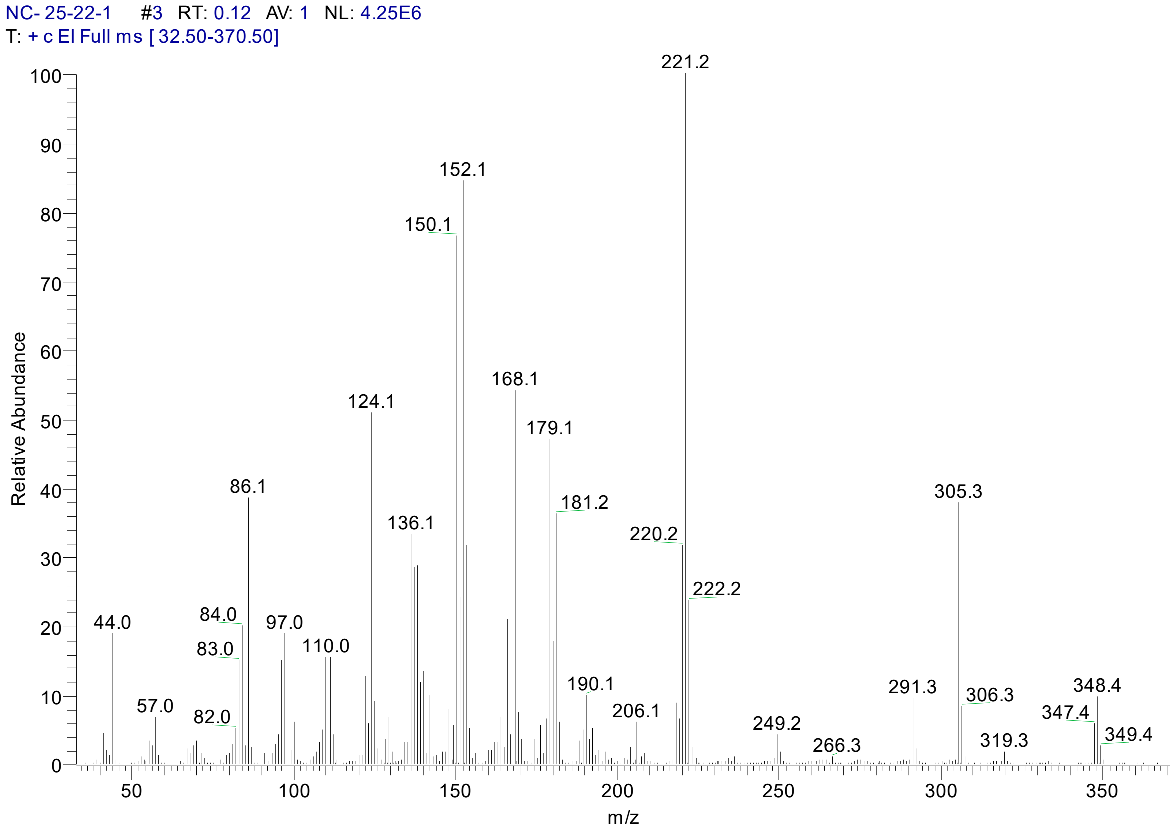

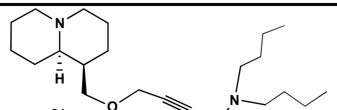

21

Tsource=100˚C

Calculated: m/z= 347.3057
(C22 H39 O1 N2)$^{+\cdot}$
Found: m/z= 347.3055

**1**	**C22 H39 O1 N2**
**RDB**	**(ppm)**
**4.5**	**-0.549**

Isotope	min	max
12 C	16	26
1 H	30	45
16 O	0	3
14 N	0	4

m/z	Intensity	Relative
44.0	805284.0	18.97
83.0	633873.0	14.93
84.0	853752.0	20.11
86.1	1635752.0	38.53
96.0	640941.0	15.10
97.0	801857.0	18.89
98.0	780959.0	18.39
110.0	654741.0	15.42
111.1	652813.0	15.38
124.1	2157421.0	50.81
136.1	1414220.0	33.31
137.1	1211109.0	28.53
138.1	1214628.0	28.61

m/z	Intensity	Relative
150.1	3248708.0	76.52
151.1	1023572.0	24.11
152.1	3593428.0	84.64
153.1	1343997.0	31.66
166.1	886798.0	20.89
168.1	2296827.0	54.10
179.1	1997546.0	47.05
180.1	751034.0	17.69
181.2	1542305.0	36.33
220.2	1349230.0	31.78
221.2	4245661.0	100.00
222.2	1006590.0	23.71
234.2	29496.0	0.69
236.2	40022.0	0.94
248.2	33933.0	0.80
249.2	181417.0	4.27
250.2	68968.0	1.62
262.2	21969.0	0.52
263.2	22306.0	0.53
264.2	21694.0	0.51
266.3	39012.0	0.92
274.3	24233.0	0.57
275.3	21021.0	0.50
288.3	28119.0	0.66
290.3	20286.0	0.48
291.3	405273.0	9.55
292.3	87816.0	2.07
302.3	21571.0	0.51
304.3	19674.0	0.46
305.3	1607510.0	37.86
306.3	355184.0	8.37
307.3	40359.0	0.95
319.3	77199.0	1.82
347.4	244724.0	5.76
348.4	412831.0	9.72
349.4	111959.0	2.64
350.4	20684.0	0.49

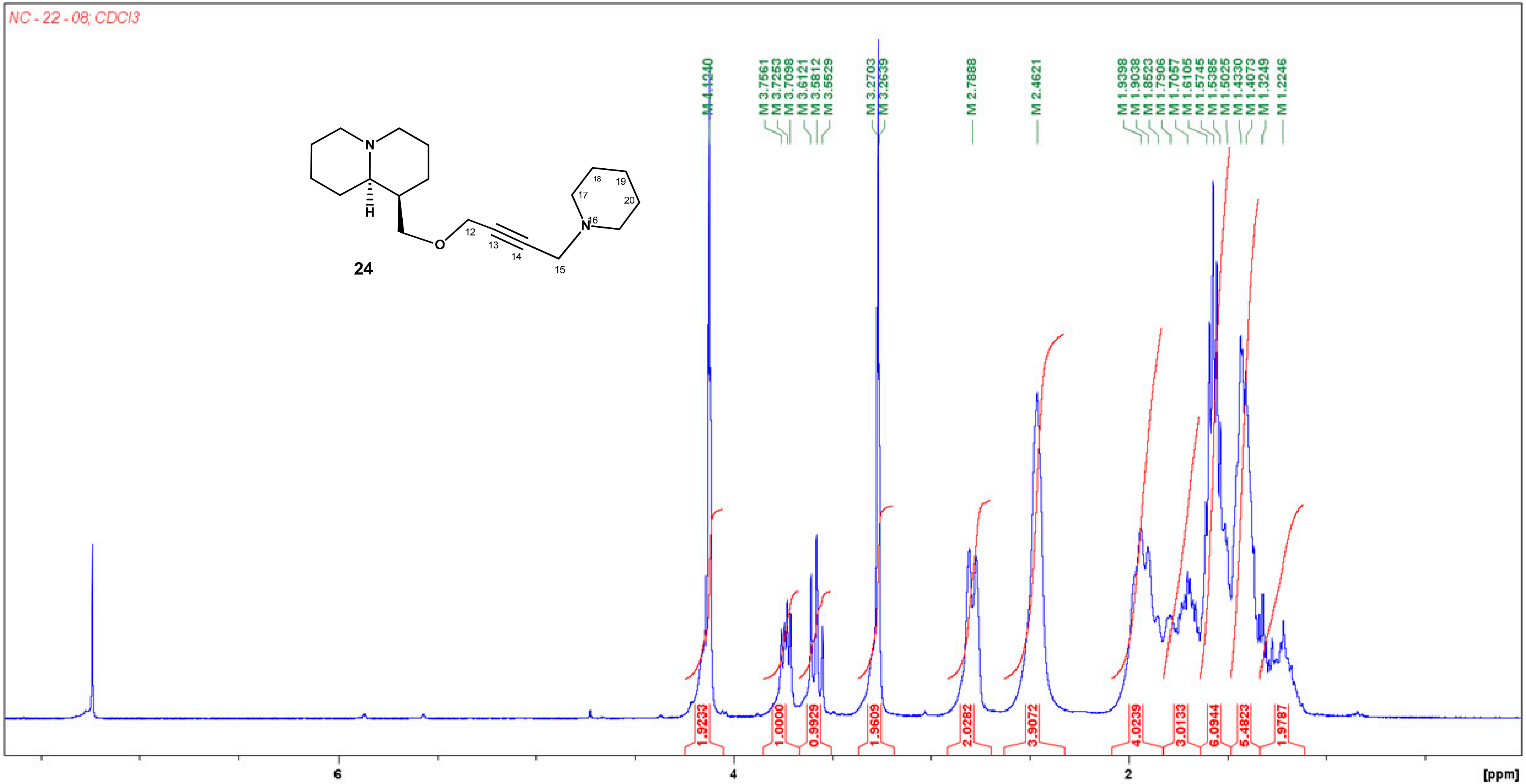

Figure S34. ^{1}H NMR spectrum of (1*R*,9a*R*)-1-((4-(piperidin-1-yl)but-2-ynyloxy)methyl)octahydro-1*H*-quinolizine (**24**) ($CDCl_3$, 300MHz).

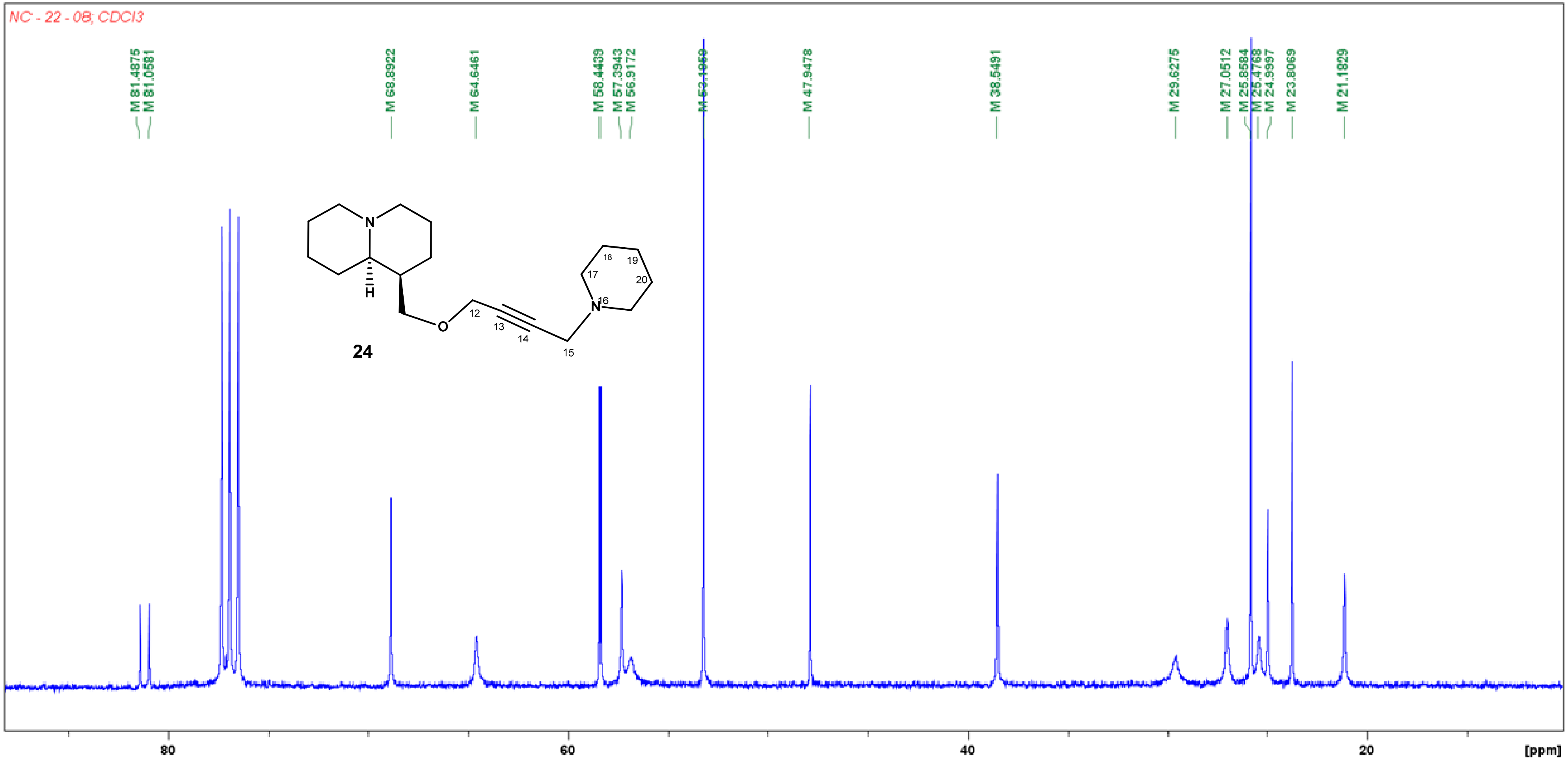

Figure S35. ^{13}C NMR spectrum of (1*R*,9a*R*)-1-((4-(piperidin-1-yl)but-2-ynyloxy)methyl)octahydro-1*H*-quinolizine (**24**) ($CDCl_3$, 100 MHz).

Figure S36. HR-MS spectrum of (1*R*,9a*R*)-1-((4-(piperidin-1-yl)but-2-ynyloxy)methyl)octahydro-1*H*-quinolizine (**24**).

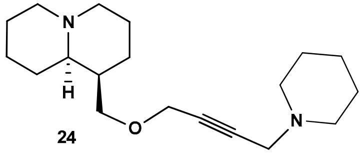

24

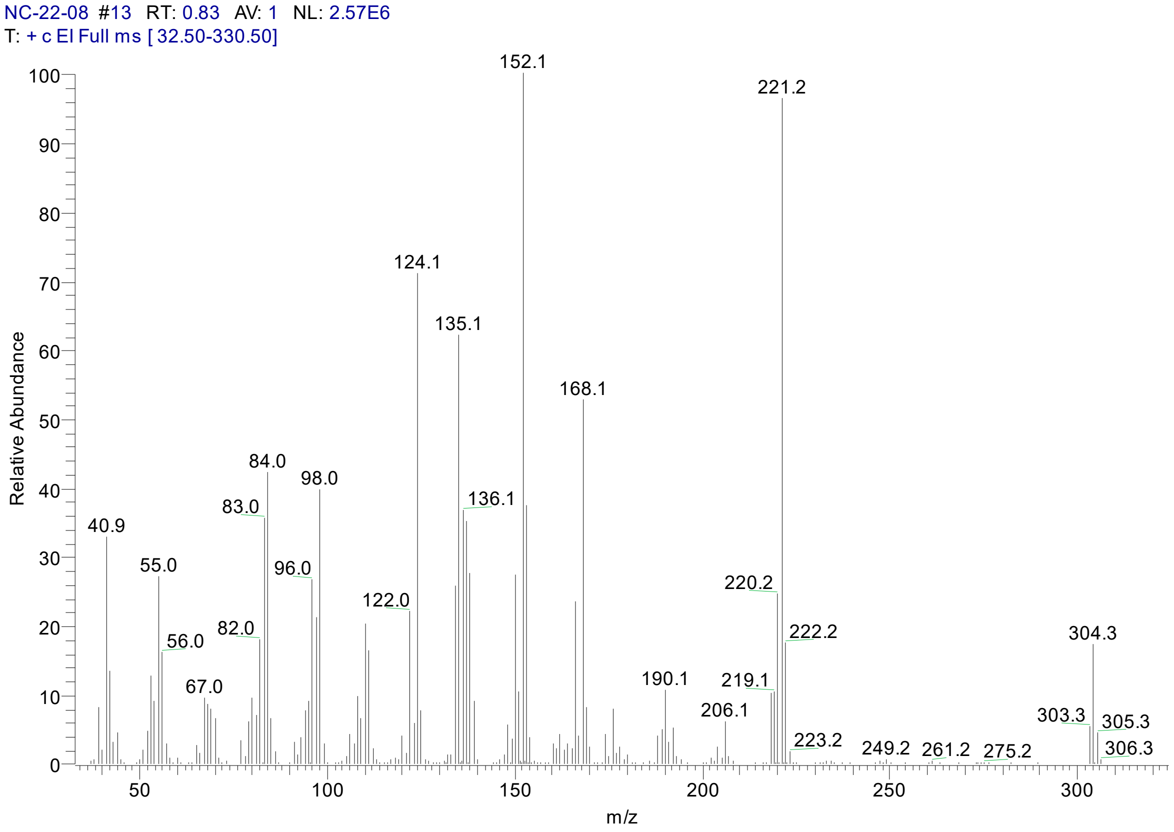

Tsource=80˚C
Tprobe=230˚C
Calculated:
m/z= 304.2509

Found:
Измеренное значение
m/z= 304.2507
1 C19 H32 O1 N2

RDB Ошибка (ppm
5 -0.708

Isotope	min	max
12 C	15	22
1 H	25	40
16 O	0	3
14 N	0	4

m/z	Intensity	Relative
38.9	205598.0	8.01
40.9	844549.0	32.92
41.9	342832.0	13.36
53.0	323582.0	12.61
54.0	234123.0	9.13
55.0	695861.0	27.12
56.0	412678.0	16.09
67.0	245103.0	9.55
68.0	219176.0	8.54
69.0	201156.0	7.84
80.0	244768.0	9.54
82.0	462827.0	18.04
83.0	913194.0	35.59
84.0	1083646.0	42.24
94.0	197747.0	7.71
96.0	681501.0	26.56
97.0	545553.0	21.26

m/z	Intensity	Relative
98.0	1015951.0	39.60
108.0	250886.0	9.78
110.0	519626.0	20.25
111.0	421730.0	16.44
122.0	566059.0	22.06
124.1	1825411.0	71.15
134.0	658424.0	25.66
135.1	1593075.0	62.10
136.1	940210.0	36.65
137.1	899811.0	35.07
138.1	707595.0	27.58
139.1	230547.0	8.99
150.1	699940.0	27.28
151.1	264297.0	10.30
152.1	2565527.0	100.00
153.1	958873.0	37.38
166.1	599027.0	23.35
168.1	1352246.0	52.71
169.1	208532.0	8.13
174.1	111111.0	4.33
176.1	200513.0	7.82
188.1	100689.0	3.92
189.1	125806.0	4.90
190.1	273265.0	10.65
191.1	77752.0	3.03
192.1	133992.0	5.22
204.1	59173.0	2.31
206.1	152795.0	5.96
207.1	27016.0	1.05
218.1	259072.0	10.10
219.1	265330.0	10.34
220.2	632692.0	24.66
221.2	2476850.0	96.54
221.3	11259.0	0.44
222.2	449836.0	17.53
223.2	43416.0	1.69
303.3	136032.0	5.30
304.3	445617.0	17.37
305.3	111725.0	4.35
306.3	13466.0	0.52

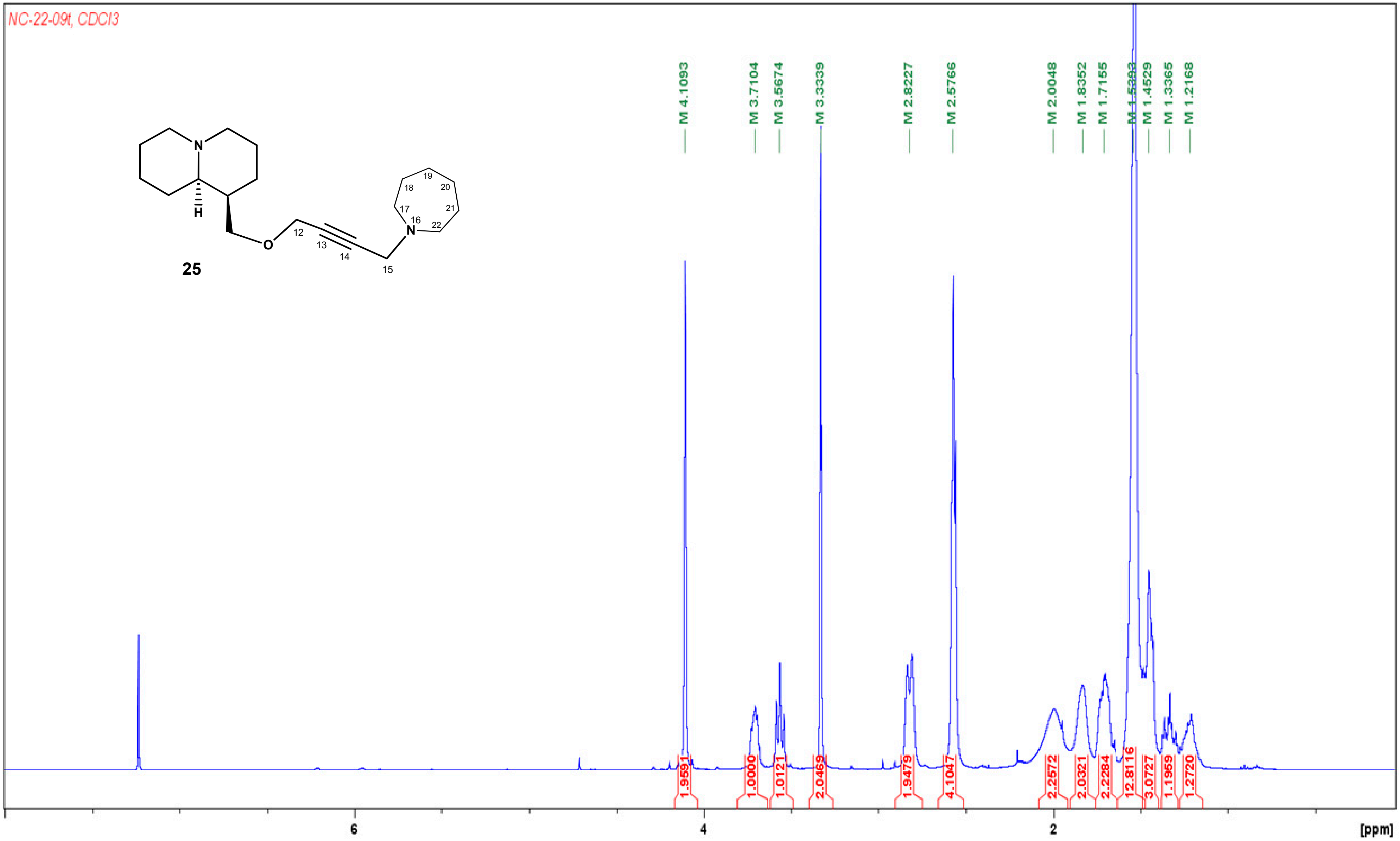

Figure S37. ^{1}H NMR spectrum of (1*R*,9a*R*)-1-((4-(azepan-1-yl)but-2-ynyloxy)methyl)octahydro-1*H*-quinolizine (**25**) ($CDCl_3$, 300 MHz).

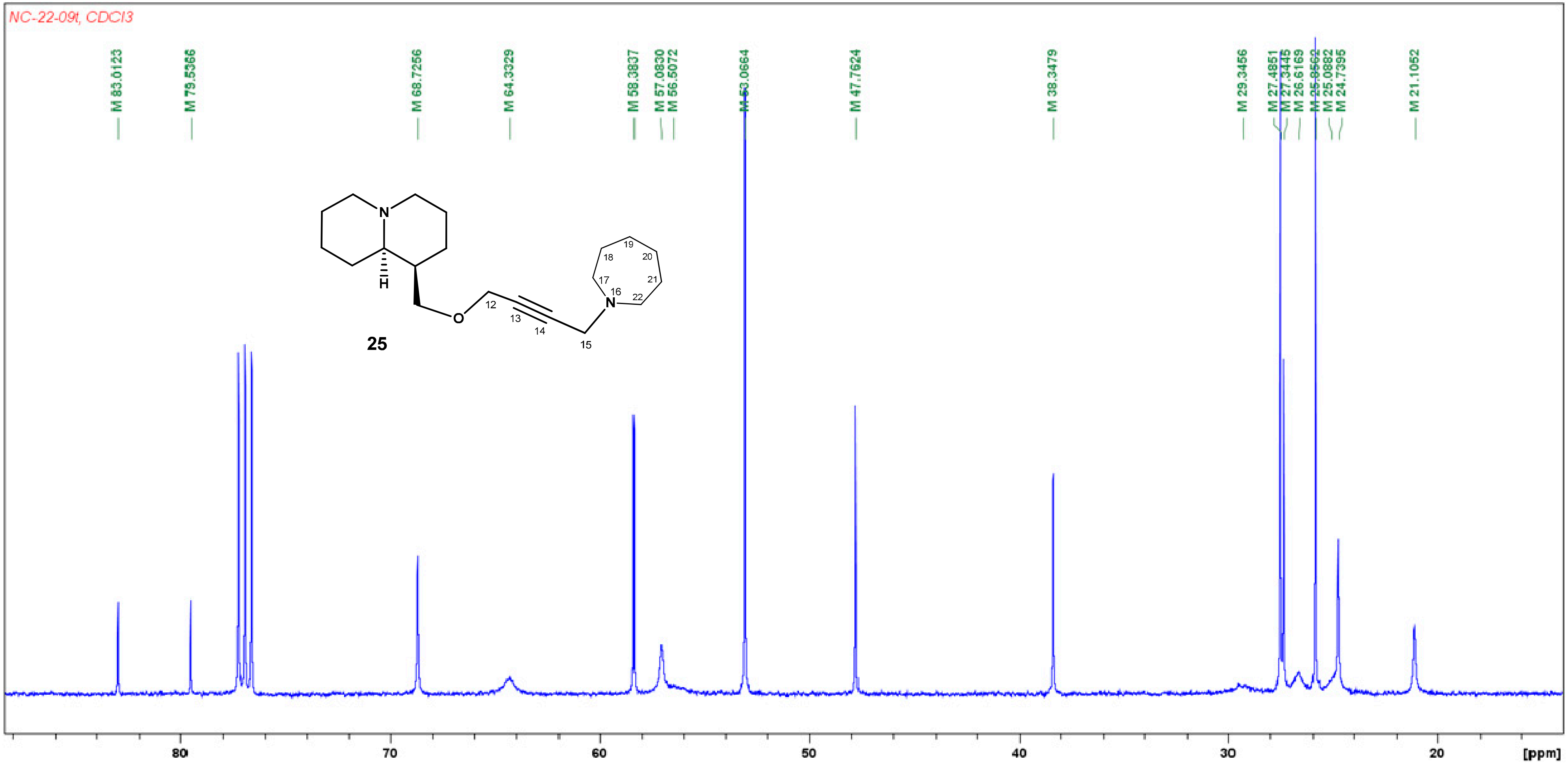

Figure S38. ^{13}C NMR spectrum of (1*R*,9a*R*)-1-((4-(azepan-1-yl)but-2-ynyloxy)methyl)octahydro-1*H*-quinolizine (**25**) ($CDCl_3$, 100 MHz).

Figure S39. HR-MS spectrum of (1*R*,9a*R*)-1-((4-(azepan-1-yl)but-2-ynyloxy)methyl)octahydro-1*H*-quinolizine (**25**).

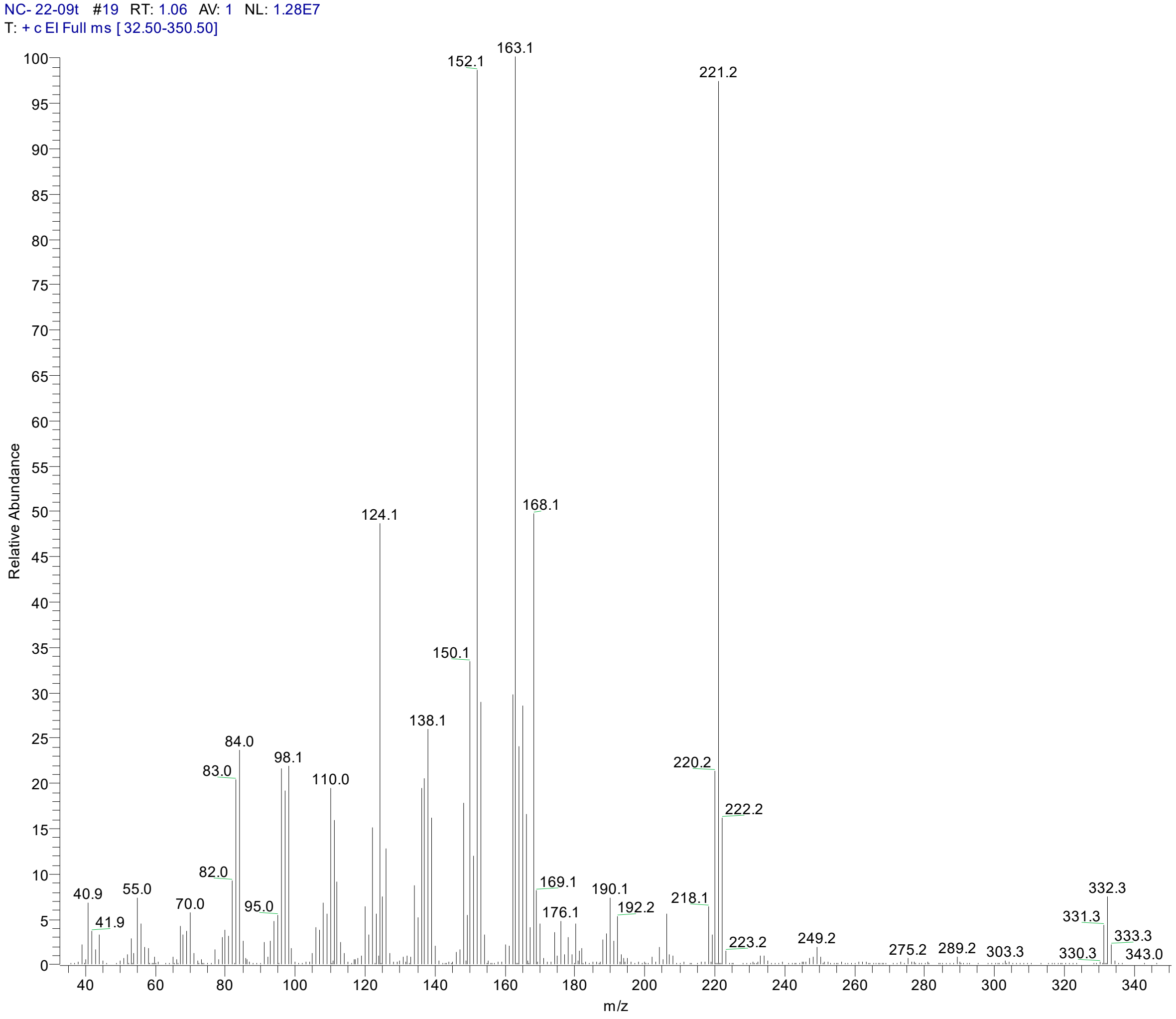

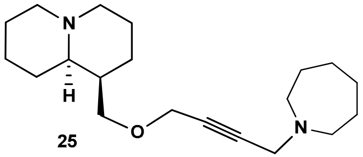

25

Tsource=90˚C

Tprobe=220˚C

Calculated: **m/z= 332.2822**

(C21 H36 O1 N2) $^{+\cdot}$

Found: **m/z= 332.2816**

**1**	**C21 H36 O1 N2**	
	**RDB**	**(ppm)**
	**5.0**	**-1.853**

Isotope	min	max
12 C	16	25
1 H	25	45
16 O	0	3
14 N	0	4

m/z	Intensity	Relative
83.0	2602643.0	20.33
84.0	3021274.0	23.60
96.0	2758544.0	21.54
97.0	2441780.0	19.07
98.1	2786065.0	21.76
110.0	2480155.0	19.37
111.1	2019661.0	15.77
122.1	1915158.0	14.96
124.1	6217055.0	48.55
126.1	1631652.0	12.74
136.1	2471007.0	19.30
137.1	2617605.0	20.44

m/z	Intensity	Relative
138.1	3308966.0	25.84
139.1	2062454.0	16.11
148.1	2273552.0	17.76
150.1	4263289.0	33.29
151.1	1518932.0	11.86
152.1	12630528.0	98.64
153.1	3700308.0	28.90
162.1	3808145.0	29.74
163.1	12804608.0	100.00
164.1	3064455.0	23.93
165.1	3645576.0	28.47
166.1	2120752.0	16.56
168.1	6351637.0	49.60
174.1	435065.0	3.40
180.1	570035.0	4.45
182.1	210633.0	1.64
188.1	343509.0	2.68
189.1	427313.0	3.34
190.1	924166.0	7.22
191.1	329322.0	2.57
192.2	668801.0	5.22
206.1	706865.0	5.52
218.1	814702.0	6.36
219.2	417002.0	3.26
220.2	2722495.0	21.26
221.2	12460800.0	97.31
222.2	2054391.0	16.04
223.2	181019.0	1.41
249.2	228386.0	1.78
331.3	550654.0	4.30
332.3	946260.0	7.39
333.3	270637.0	2.11

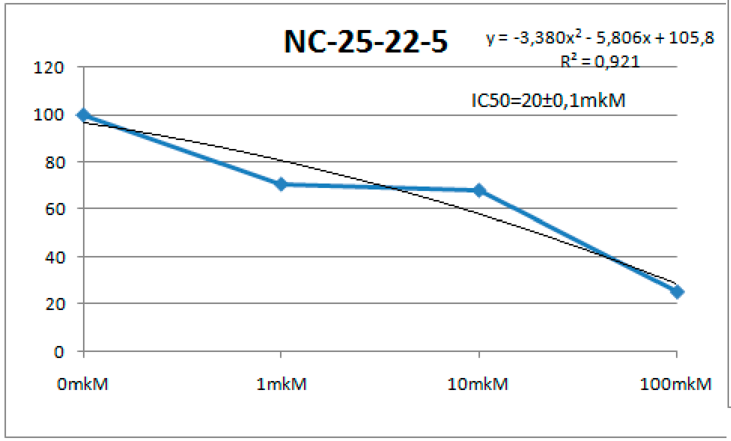

Compound **5**: $IC_{50} = 20.1 \pm 0.1$ μM

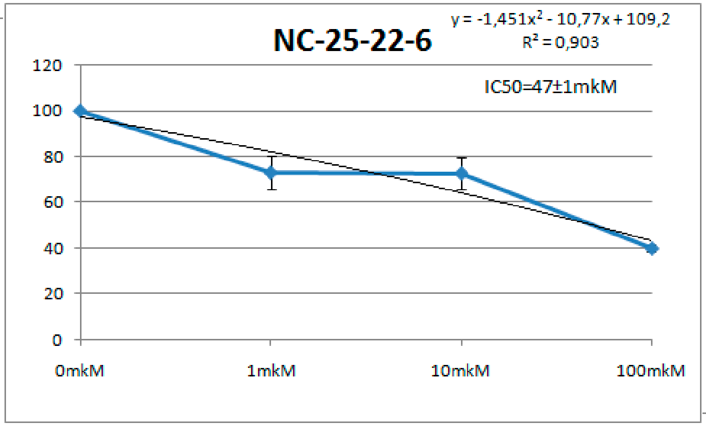

Compound **7**: $IC_{50} = 47.0 \pm 1.1$ μM

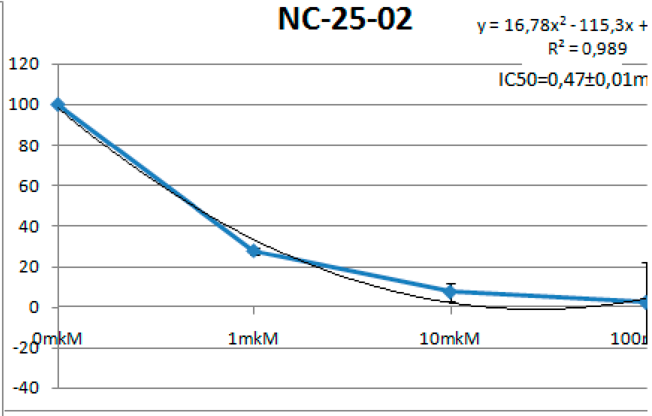

Compound **11**: $IC_{50} = 0.47 \pm 0.01$ μM

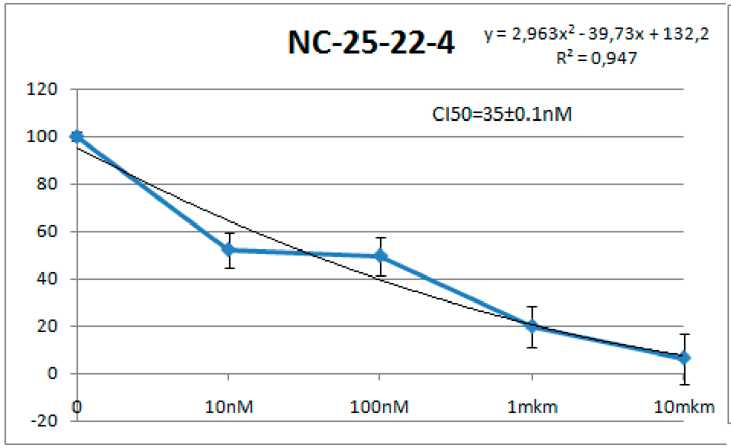

Compound **12**: $IC_{50} = 0.035 \pm 0.0001$ μM

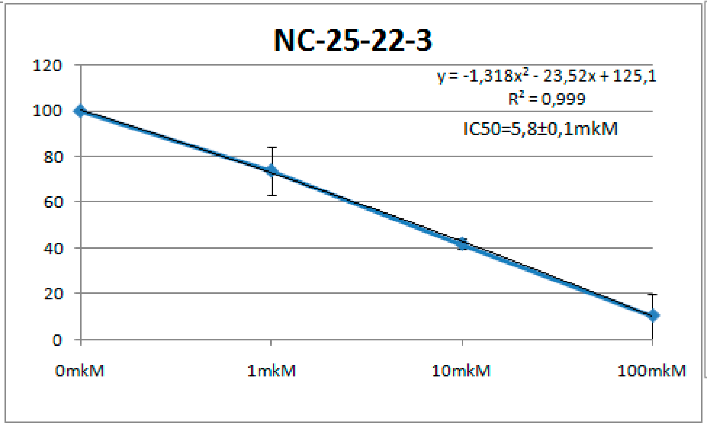

Compound **13**: $IC_{50} = 5.8 \pm 0.1$ μM

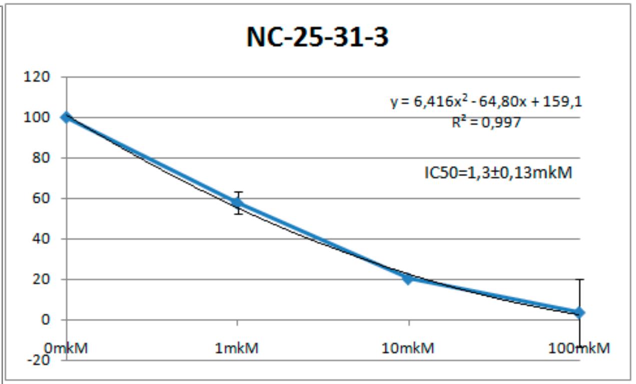

Compound **15**: $IC_{50} = 1.3 \pm 0.13$ μM

Figure S40A–F: The curves for AChE inhibition by compounds **5**, **7**, **11**, **12**, **13**, **15.**

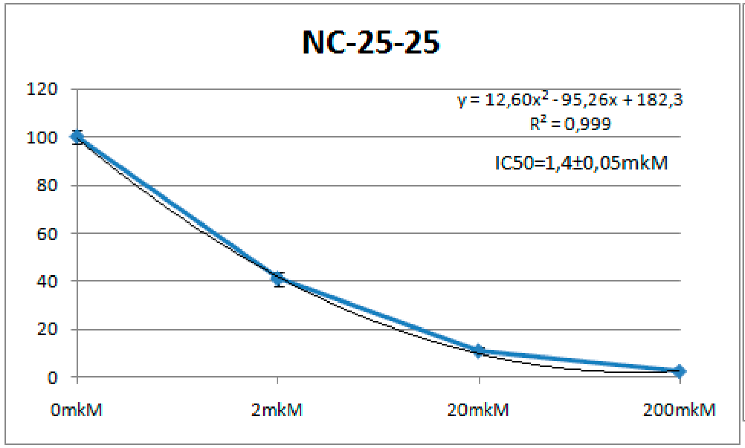

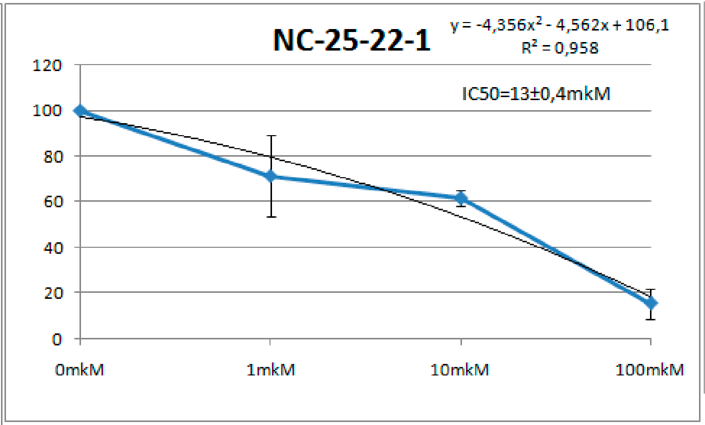

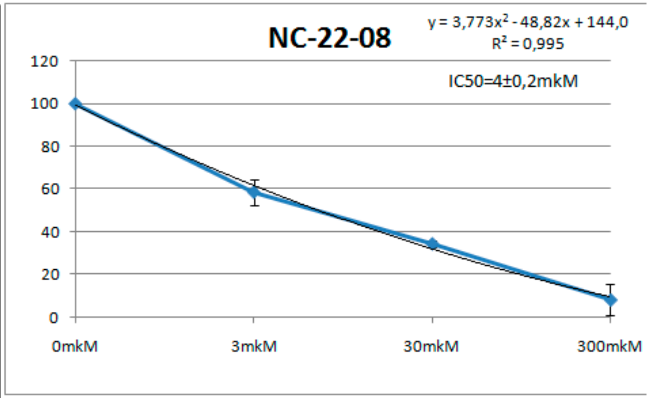

Compound **16**: IC_{50} = 1.4±0.05 µM

Compound **21**: IC_{50} = 13±0.4 µM

Compound **24**: IC_{50} = 4.0±0.2 µM

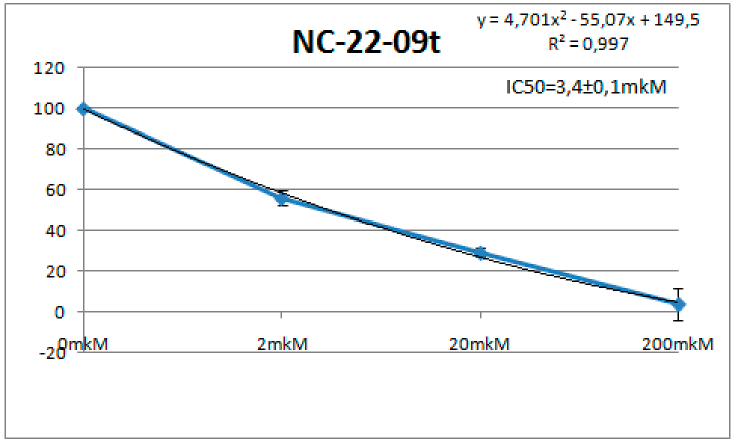

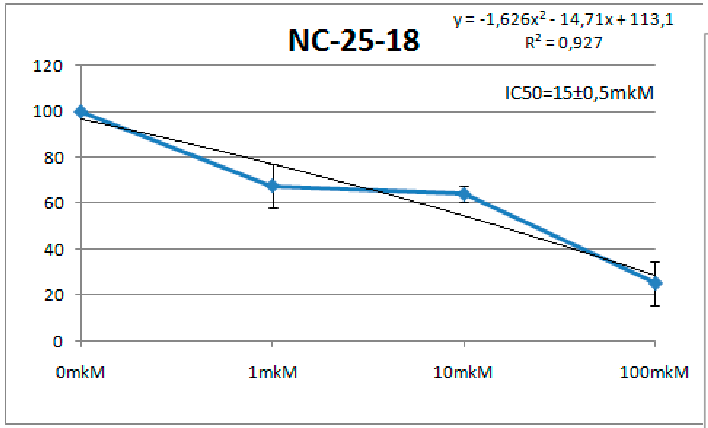

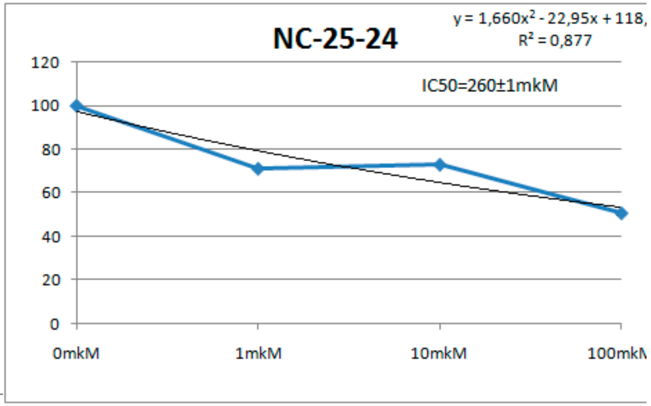

Compound **25**: IC_{50} = 3.4±0.1 µM

Compound **3**: IC_{50} = 15.0±0.5 µM

Compound **4**: IC_{50} = 260.0±1.1 µM

Figure S40G–L: The curves for AChE inhibition by compounds **16**, **21**, **24**, **25**, **3**, **4.**
